# Supplementary material for: Can moral reasoning be modeled in an experiment?
Source: PLoS One. 2021 Jun 10;16(6):e0252721. doi: 10.1371/journal.pone.0252721 (PMC8191952; doi:10.1371/journal.pone.0252721)
Supplement: S1 Appendix — https://doi.org/10.6084/m9.figshare.14703495.v1. (DOCX) [file pone.0252721.s001.docx]

S1 APPENDIX

**Ethical Story Modeling Experiment Report Form**

First and last name of the subject Personal code of the student Year of study

………………………………………………….. ………………………………………….. ………………….

Order numbers of ethical stories generated by random number generator, with the signs of their indicator values:

(a) IAO order number….. (…. …. ….) (b) IAO order number….. (…. …. ….)

(c) KCR order number….. (…. …. ….) (d) KCR order number….. (…. …. ….)

The order number of the modeled ethical story…..

Signs of indicators of morality The ethical story

I (…. …. ….)…………………………………………………………………………………………………………………………….. ………………………………………………………………………………………………………………………………………………A (…. …. ….)…………………………………………………………………………………………………………………………….. ………………………………………………………………………………………………………………………………………………O (…. …. ….)…………………………………………………………………………………………………………………………….. ………………………………………………………………………………………………………………………………………………

The overall moral evaluation of the ethical story is: [ ]

(b) The order number of the modeled ethical story…..

Signs of indicators of morality The ethical story

I (…. …. ….)…………………………………………………………………………………………………………………………….. ………………………………………………………………………………………………………………………………………………A (…. …. ….)…………………………………………………………………………………………………………………………….. ………………………………………………………………………………………………………………………………………………O (…. …. ….)…………………………………………………………………………………………………………………………….. ………………………………………………………………………………………………………………………………………………

The overall moral evaluation of the ethical story is: [ ]

(c) The order number of the modeled ethical story…..

Signs of indicators of morality The ethical story

K (…. …. ….)…………………………………………………………………………………………………………………………….. ………………………………………………………………………………………………………………………………………………C (…. …. ….)…………………………………………………………………………………………………………………………….. ………………………………………………………………………………………………………………………………………………R (…. …. ….)…………………………………………………………………………………………………………………………….. ………………………………………………………………………………………………………………………………………………

The overall moral evaluation of ethical story is: [ ]

(d) The order number of the modeled ethical story…..

Signs of indicators of morality The ethical story

K (…. …. ….)…………………………………………………………………………………………………………………………….. ………………………………………………………………………………………………………………………………………………C (…. …. ….)…………………………………………………………………………………………………………………………….. ………………………………………………………………………………………………………………………………………………R (…. …. ….)…………………………………………………………………………………………………………………………….. ………………………………………………………………………………………………………………………………………………

The overall moral evaluation of the ethical story is: [ ]
