# Supplementary material for: Can moral reasoning be modeled in an experiment?
Source: PLoS One. 2021 Jun 10;16(6):e0252721. doi: 10.1371/journal.pone.0252721 (PMC8191952; doi:10.1371/journal.pone.0252721)
Supplement: S2 Appendix — https://doi.org/10.6084/m9.figshare.14703504.v1. (DOCX) [file pone.0252721.s002.docx]

S2 APPENDIX- CASE STUDY EXAMPLE

**Ethical Story Modeling Experiment Report Form**

First and last name of the subject Personal code of the student Year of study

………………………………………………….. ………………………………………….. ………………….

Order numbers of ethical stories generated by random number generator, with the signs of their indicator values:

(a) IAO order number….. (…. …. ….) (b) IAO order number….. (…. …. ….)

(c) KCR order number….. (…. …. ….) (d) KCR order number….. (…. …. ….)

The order number of the modeled ethical story 2

Signs of indicators of morality The ethical story

I (+) When Jack got his driving license, he decided that he would never drive a car after drinking alcohol.

A (-) Once, when he was drinking with his friends, he picked up a call from his mother asking him to go pick her up at the station.

O (+) When he came to the garage to get the car, he noticed that his father had already gone to get his mother.

The overall moral evaluation of the ethical story is: [ - ]

(b) The order number of the modeled ethical story 4

Signs of indicators of morality The ethical story

I (+) Radek read about someone in the newspaper who needed money for a difficult operation and wanted to help him.

A (+) He took the money from the saved funds and transferred it to the indicated account.

O (-) However, it soon turned out that he had supported a fraudster who needed money for gambling.

The overall moral evaluation of the ethical story is: [ + ]

(c) The order number of the modeled ethical story 38

Signs of indicators of morality The ethical story

K (-)Isidore, a candidate for psychology, did not know about confidentiality in counseling under the psychologist's code of ethics.

C (-) Isidore is against keeping secrets because, according to him, everyone has a right to information.

R (Ø) He has not completed his psychological studies, as it is forbidden for a psychologist to pass on such information to third parties.

The overall moral evaluation of ethical story is: [ - ]

(d) The order number of the modeled ethical story 47

Signs of indicators of morality The ethical story

K (+)Monika knows that the Netherlands, as the first European country, permits euthanasia by law.

C (-)She approves of the law because it legally allows people to get rid of terminally ill relatives.

R (Ø) Considering that such a law has not been enacted in our country, it was not possible to euthanize her relatives.

The overall moral evaluation of the ethical story is: [ - ]
