# Supplementary material for: Can moral reasoning be modeled in an experiment?
Source: PLoS One. 2021 Jun 10;16(6):e0252721. doi: 10.1371/journal.pone.0252721 (PMC8191952; doi:10.1371/journal.pone.0252721)
Supplement: Attachment — Submitted filename: PONE-D-20-37673_reviewed WHITEpdf.pdf [file pone.0252721.s003.pdf]

PLOS ONE

Can moral reasoning be modeled in an experiment?

--Manuscript Draft--

**Manuscript Number:**	PONE-D-20-37673
**Article Type:**	Research Article
**Full Title:**	Can moral reasoning be modeled in an experiment?
**Short Title:**	The experiment on moral reasoning
**Corresponding Author:**	Piotr Janusz Mamcarz, Doctor<br>The John Paul II Catholic University of Lublin<br>Lublin, POLAND
**Keywords:**	moral reasoning; decision making; experiment; moral indicators
**Abstract:**	A review of the literature on moral issues indicates that none of the empirical approaches to moral reasoning proposes experimental approach which controls in one research procedure such object-related experimental variables like: motivation, acceptance of moral norms and consequences of human behavior in moral situations.<br>A unique element of proposed experimental method is a multi-stage model determining morality indicators. In the two-phase design experiment, 147 educational psychology students were asked to create model ethical stories and then make an overall assessment of each of these stories.<br>As a result, a base of ethical stories was created with empirical moral indicators (positive, negative, neutral). The patterns in the moral evaluation of ethical stories were determined by identifying three processes (selection, differentiation and integration).<br>The final result is a confirmed design of the experiment and a set of formulas that can be used in education and research on morality reasoning.<br>Key words: cognitive schemas of ethical-dilemma-stories; modeling of ethical stories; moral reasoning experiment; morality indicators; overall moral evaluation of ethical stories; synthetic approach to intelligence.
**Order of Authors:**	Ján Grác
	Adam, Biela
	Piotr Janusz Mamcarz, Doctor
	Dorota Kornas-Biela
**Additional Information:**	
**Question**	**Response**
**Financial Disclosure**<br><br>Enter a financial disclosure statement that describes the sources of funding for the work included in this submission. Review the submission guidelines for detailed requirements. View published research articles from *PLOS ONE* for specific examples.<br><br>This statement is required for submission and **will appear in the published article** if the submission is accepted. Please make sure it is accurate.	NO

Powered by Editorial Manager® and ProduXion Manager® from Aries Systems Corporation

**Unfunded studies**<br>Enter: *The author(s) received no specific funding for this work.*<br><br>**Funded studies**<br>Enter a statement with the following details:<br>• Initials of the authors who received each award<br>• Grant numbers awarded to each author<br>• The full name of each funder<br>• URL of each funder website<br>• Did the sponsors or funders play any role in the study design, data collection and analysis, decision to publish, or preparation of the manuscript?<br>• **NO** - Include this sentence at the end of your statement: *The funders had no role in study design, data collection and analysis, decision to publish, or preparation of the manuscript.*<br>• **YES** - Specify the role(s) played.<br><br>* typeset	
**Competing Interests**<br><br>Use the instructions below to enter a competing interest statement for this submission. On behalf of all authors, disclose any competing interests that could be perceived to bias this work—acknowledging all financial support and any other relevant financial or non-financial competing interests.<br><br>This statement **will appear in the published article** if the submission is accepted. Please make sure it is accurate. View published research articles from *PLOS ONE* for specific examples.	NO

Powered by Editorial Manager® and ProduXion Manager® from Aries Systems Corporation

**NO authors have competing interests**<br><br>Enter: *The authors have declared that no competing interests exist.*<br><br>**Authors with competing interests**<br><br>Enter competing interest details beginning with this statement:<br><br>*I have read the journal's policy and the authors of this manuscript have the following competing interests: [insert competing interests here]*<br><br>* typeset	
**Ethics Statement**<br><br>Enter an ethics statement for this submission. This statement is required if the study involved:<br><br>• Human participants<br>• Human specimens or tissue<br>• Vertebrate animals or cephalopods<br>• Vertebrate embryos or tissues<br>• Field research<br><br>Write "N/A" if the submission does not require an ethics statement.<br><br>General guidance is provided below. Consult the submission guidelines for detailed instructions. **Make sure that all information entered here is included in the Methods section of the manuscript.**	The Deparment of Psychology Ethical Board (Trnava University). This reasearch was approved by this committee. The data were analyzed anonymously but the oral consent was obtained before the subject research.

Powered by Editorial Manager® and ProduXion Manager® from Aries Systems Corporation

**Format for specific study types**<br><br>**Human Subject Research (involving human participants and/or tissue)**<br>• Give the name of the institutional review board or ethics committee that approved the study<br>• Include the approval number and/or a statement indicating approval of this research<br>• Indicate the form of consent obtained (written/oral) or the reason that consent was not obtained (e.g. the data were analyzed anonymously)<br><br>**Animal Research (involving vertebrate animals, embryos or tissues)**<br>• Provide the name of the Institutional Animal Care and Use Committee (IACUC) or other relevant ethics board that reviewed the study protocol, and indicate whether they approved this research or granted a formal waiver of ethical approval<br>• Include an approval number if one was obtained<br>• If the study involved *non-human primates*, add *additional details* about animal welfare and steps taken to ameliorate suffering<br>• If anesthesia, euthanasia, or any kind of animal sacrifice is part of the study, include briefly which substances and/or methods were applied<br><br>**Field Research**<br><br>Include the following details if this study involves the collection of plant, animal, or other materials from a natural setting:<br>• Field permit number<br>• Name of the institution or relevant body that granted permission	
**Data Availability**<br><br>Authors are required to make all data underlying the findings described fully available, without restriction, and from the time of publication. PLOS allows rare exceptions to address legal and ethical concerns. See the PLOS Data Policy and FAQ for detailed information.	No - some restrictions will apply

A Data Availability Statement describing where the data can be found is required at submission. Your answers to this question constitute the Data Availability Statement and **will be published in the article**, if accepted.<br><br>**Important:** Stating 'data available on request from the author' is not sufficient. If your data are only available upon request, select 'No' for the first question and explain your exceptional situation in the text box.<br><br>Do the authors confirm that all data underlying the findings described in their manuscript are fully available without restriction?	
**Describe where the data may be found in full sentences. If you are copying our sample text, replace any instances of XXX with the appropriate details.**<br><br>• If the data are **held or will be held in a public repository**, include URLs, accession numbers or DOIs. If this information will only be available after acceptance, indicate this by ticking the box below. For example: *All XXX files are available from the XXX database (accession number(s) XXX, XXX.).*<br>• If the data are all contained **within the manuscript and/or Supporting Information files**, enter the following: *All relevant data are within the manuscript and its Supporting Information files.*<br>• If neither of these applies but you are able to provide **details of access elsewhere**, with or without limitations, please do so. For example:<br><br>*Data cannot be shared publicly because of [XXX]. Data are available from the XXX Institutional Data Access / Ethics Committee (contact via XXX) for researchers who meet the criteria for access to confidential data.*<br><br>*The data underlying the results presented in the study are available from (include the name of the third party*	The data underlying the results presented in the study can be access directly on the Trnava University because the materials are in paper form. If there will be request that data can be digitised.

and contact information or URL).<br>• This text is appropriate if the data are owned by a third party and authors do not have permission to share the data.<br><br>* typeset	
Additional data availability information:	

Powered by Editorial Manager® and ProduXion Manager® from Aries Systems Corporation

Manuscript Click here to access/download;Manuscript;Manuscript body PLOS.doc

Can moral reasoning be modeled in an experiment?

Ján Grác

Department of Psychology

Trnava University

Hornopotočná 23, 918 43 Trnava, Slovakia

e-mail: grac.j.z.2@pobox.sk

Adam Biela

Department of Social Psychology

Maria Sklodowska-Curie University

plac Marii Curie-Skłodowskiej 5, 20-400, Lublin, Poland

e-mail: adam@biela.pl

ORCID ID: 0000-0001-5730-7693

Piotr Mamcarz - Corresponding author

Department of Emotion and Motivation Psychology

The John Paul II Catholic University of Lublin

Al. Racławickie 14, 20-950 Lublin, Poland

e-mail: pmamcarz@kul.pl

Phone. +48 509 630 598 Fax +48 81 445 33 40

ORCID ID: 0000-0001-6307-6326

Dorota Kornas-Biela

Department of Psychopedagogy

The John Paul II Catholic University of Lublin

Al. Racławickie 14, 20-950 Lublin, Poland

e-mail: dorota@biela.pl

ORCID ID: 0000-0001-6002-1901

Can moral reasoning be modeled in an experiment?

Abstract

A review of ~~the~~ literature on moral issues indicates that none of the empirical approaches to moral reasoning proposes
an
experimental approach which controls in one research procedure such object-related experimental variables like: motivation, acceptance of moral norms and consequences of human behavior in moral situations.

One sentence paragraphs are to be avoided.

the present

A unique element of proposed experimental method is a multi-stage model determining morality indicators.
Employing a
~~In the~~ two-phase design experiment, 147 educational psychology students were asked to create model ethical stories and then make an overall assessment of each of these stories. Does each student assess every story, only their own, or…?

the model determines these indicators, or is constructed from them?

As a result, a base of ethical stories was created with empirical moral indicators (positive, negative, neutral). The patterns in the moral evaluation of ethical stories were determined by identifying three processes (selection, differentiation and integration). The final result is a confirmed design of the experiment and a set of formulas that can be used in education and research on morality reasoning.

Key words: cognitive schemas of ethical-dilemma-stories; modeling of ethical stories; moral reasoning experiment; morality indicators; overall moral evaluation of ethical stories; synthetic approach to intelligence.

INTRODUCTION

Empirical studies on moral reasoning are well known in the psychological and educational literature (Rest, 1975; Candee, 1976; Czyżowska, Niemczyński, 2006; Joyce, 2006; Williams & Gantt, 2012; Alfano, 2019). Most of them are inspired by the two early

concepts on structure and choice in moral reasoning given by Piaget and Kohlberg. More recent studies on moral thinking are related with the concept of *moral intelligence* and its developments (see: Kornas-Biela, Schwarz, Biela-Wołońciej, 2018; Kornas-Biela, 2019).

Piaget (1932, 1957) began his research on moral development using the clinical-experimental approach. This approach was based on short dialogues with individual small children in the context of a moral issue situation. The dialogue with a child, called an *indagation*, aimed to ascertain his or her individual understanding of moral concepts. Piaget proposed a model of stages of moral reasoning which states as one of its premises that qualitative changes in the structure cause the appearance of a new stage at specific points in time. It could be said that he began a methodological revolution, which shifted psychologists' orientation from analyzing an individual`s behavior or choice to the underlying form and structure of moral reasoning.

Are these points in time, or periods in the more or less normal biological development of human beings?

Another approach by Kohlberg (1969, 1971) is based on hypothetically constructed moral dilemmas and aims to test moral decision-making in youth and adults. Kohlberg traced the development of moral structures through a series of six stages which form an invariant and culturally universal sequence. Moral structures are measured by requesting individuals to reason about cases in which two or more conventional values are in conflict. The subject reacts to a moral dilemma, indicating what ought to be done and justifying this course of action. The interviewer tries to elicit and probe the subject`s views without interjecting or suggesting any thinking different from the subject`s own spontaneous thinking. The subjects' behavior is interpreted in terms of levels of moral reasoning. Recently, still new authors in psychological investigations of moral reasoning frequently employ moral dilemmas (e.g. Yiym & Song, 2017; Crockett, 2013; Paciello et al., 2013; Cushman, Young & Hauser, 2006;

Greene et al., 2009; Greene, Morelli, Lowenberg, Nystrom & Cohen, 2008; Haidt, 2007; Moll & de Oliveira-Souza, 2007).

Restrictions experienced in the measurement of the structure of moral reasoning has inspired researchers to search for some standardized, objectively scorable tools of moral judgment. A significant contribution in this regard was made by Rest (1973). Studying how people choose important issues in moral dilemmas, he and his colleagues invented a procedure called the Defining Issues Test (DIT) which requires a subject to read a hypothetical moral dilemma and then select those statements that are the most important issues in making a decision about the case from among 12 statements. The research he conducted, however, still confirmed that difficulties exist in the assessment of moral judgment with an objective test (Rest, 1975). That is why Wilmoth and McFarlang (1977) carried out a comparative analysis of psychometric values of the psychometric tools which measure moral reasoning.

Is this from Rest, 1973, or 75?

Insights from the *Defining Issues Test* on moral reasoning competencies was taken into some applied fields (e.g. in service-learning e-tutors by Chien et al., 2016; educational intervention in community pharmacists by Roche & Thoma, 2017). However, the question still remains as to whether is it sufficient and appropriate to study moral judgment using a standardized test with structured responses.

The problem of ethical valuation of moral behavior

Some theories of moral development (cf. Kohlberg, 1971; Gibbs, 2003) focused on moral reasoning describe the way in which a person understands and interprets social

Can you write this more simply, perhaps as multiple sentences?

situations and directs themselves when it comes to solving moral dilemmas. The relationship

Maybe this is a better first sentence for this paragraph>

between moral reasoning and moral action is complex. Kohlberg represented the position that moral judgment is a necessary but not always sufficient condition for moral action (Kohlberg & Candee, 1984). More recent studies show that competence of moral reasoning, understood in terms of the Kohlberg's theory as a measure of moral development, correlates negatively with bullying at school in youngsters (von Grundherr et al., 2017). In turn, Fang et al. (2017) found that the highest level of moral reasoning is associated with increased

task in?

ventral striatal activity at rest and during task in students.

However, moral evaluation is dilemmatic primarily because of the one-dimensionality in which it continues to be formulated in moral judgment literature. In this study, dilemmas will be presented as a multidimensional reality, which allows us to take a new research approach to moral dilemmas.

A motivating contribution to understanding moral reasoning was introduced by British analytical philosophy, developed by the fertile minds of analytical philosophers such as G.E. Moore, A. N. Whitehead and B. Russell. They incorporated a multidimensional

Was this somehow lacking in theories, prior?

approach in the analysis of moral assessment. For example, Moore (2005) claims that a human action (act) can be evaluated as morally good or bad only in the context of a given situation and the effect of the person's free choice.

Moore's analysis of psychological factors, which defines the intentions contained in the motives of the act and the feelings associated with it (i.e. experienced feelings), is a prerequisite for understanding moral judgment. In a review and synthesis of his analysis, Moore states that nothing can be inherently good or bad if does not contain some emotion. That is why Moore's ethical system is named *emotivism*.

According to Moore, a substantial value in the process of moral evaluation is the motivation of the act. A general psychological predisposition to right proceedings corresponds to good motives, although right proceedings are not always an effect of these motives. Before acting, a subject may have good intentions, but he or she is not able to predict the results of their decision. This is due to both the cognitive limitation of the human being and the risky nature of the decisive situation in which the individual is placed, where hypothetical states of reality constitute the set of uncertain events, for which we can only determine degrees of probability.

Ellemers et al. (2019) presented a monumental metaanalysis of the psychology of morality empirical studies published from 1940 through 2017. Analogically to Moore's analysis, these authors distinguished the moral studies from the non-moral ones according to the criterion of considering in the research the problem of what is right (morally good) vs. wrong (morally bad) way to behave. In the first stage of their metaanalysis they distinguished five relatively independent areas of morality research: moral reasoning, moral behavior, moral judgment, moral self-view, moral emotions. In the next stage, relationships between these areas were shown and types of measurement scales, which were used by the authors of the analyzed studies. This metaanalysis enabled to draw the two conclusions: (1) different life experiences and social roles can have a long-term impact on the way people reason about morality and the moral principles they prioritize; (2) more immediate situational cues also affect moral reasoning and moral decisions. These conclusions on moral reasoning complement philosophical analyzes as well as evolutionary approaches, highlighting the objective value of survival morality by following specific principles or guidelines (Shou and Song, 2017).

The text would benefit greatly with a more direct engagement with foundational resources. Many works are referenced for substantiation of the authors' position, but few of these are directly used in development of the position and so in the clarification of the position. For instance, Rest 73 and 75 - what happens between these two works that may be important to the story that you are telling, because you reference these first 73 at the beginning of a discussion, and then the other at the end and it is not clear where one begins and the other leaves off. Facile engagements with core resources wil do a few things for you. One, it makes the paper more interesting. Two, it gives you are chance to set up where in the field this present work sits, and why it is important. Right now, this is not clear. Then, at the end of the introduction, there is this shift to analytic philosophy from (broadly developmental) psychology. This introduction needs to be recomposed.

What are the methodological conclusions from this meta-analysis regarding the empirical research on morality? Its authors (see: Ellemers et al., 2019) clearly indicate that studies on morality have largely neglected to examine how moral reasoning relates to actions in such experimental contexts, such as perpetrated harm or moral norms and intentions.

This is not clear from the preceding discussion. And, why "see: Ellemers" here?

More recent metaanalysis studies on moral reasoning have allowed us to state that this research is focused on the following three points: (1) operationalization of moral reasoning abilities by constructing the psychometric tests and measuring their validity as well as reliability (Choi et al., 2019; Patil et al., 2020); (2) inventing experimental approaches on moral reasoning: laboratory experiment (Sudić & Ćirić, 2019); on-line experiment (Adrian & Gehrlein, 2020); and moral reasoning thought experiment (Rhim, Lee G. & Lee, J., 2020); and (3) applied research on moral reasoning in some particular professional domains where morality and ethics is considered to be an important part of professional competencies, such as health care professionals (pharmacists, e.g. Lezley-Anne, Gillen & Hall, 2017; Göttgens-Jansen et al., 2019; nurses, e.g. Kamali, Yousefy, & Yamani, 2019), businessmen (Nathan, 2014); sportsmen (Calmeiro, Stoll, & Davis, 2015), and computer scientists (Rhim, Lee G. & Lee, J., 2020).

this is not a "point"

Summarizing the above research findings one can state the following outcomes, among others: (a) moral reasoning is a multi-faceted phenomenon where its mutually linked factors constitute the moral existence of the individual human person; (b) there is a need to distinguish and differentiate the following components of moral reasoning processes for both theoretical and practical purposes: cognitive, volitional, emotional, motivational (intentional) and behavioral processes – which the literature refers to as indicators of morality; (c) psychology is still far from determining the external validity of its psychometric

You mean the literature so far cited? Other? Anything in particular? Should we have some idea of which, by now?

and experimental instruments for studying moral reasoning in 'natural experiment' circumstances with a higher ecological validity.

In the context of the above statements, one can pose the question: Is it possible to design an experiment on moral reasoning in circumstances of higher ecological validity, and if so, in what way?

Unfortunately, none of the above approaches to moral reasoning propose any form of research which controls for object-related experimental variables, such as those related to motivation, acceptance of moral norms, and consequences of human behavior in moral situations – in a single research procedure. We believe that it is possible, and we demonstrate this in natural experiment conditions, where subjects had as a task to define their real-life moral issue as a situation characterized by moral norms involved with the social-moral environment of their professional or educational endeavor. Such arrangements guarantee appropriate circumstances for significantly higher ecological validity of the experimental procedure.

You should prove this to be the case somehow, perhaps with a table suggesting the difference between your approach and all of those reviewed in passing discussion, above. If we could see such a table, then we could easily understand why your effort at "controlling for object related exp vars" is novel and important.

Moreover, we can assume that moral reasoning is a process where the subject

Why should we assume this?

conducts a moral evaluation of a human event based upon some premises (Bucciarelli,

How are premises and event related?

Khemlani, & Johnson-Laird, 2008). The premises for him or her are the indicators of morality which are analyzed in the particular situations, and then synthetized as an overall moral assessment of their activity (or non-activity) under the given social environment. Thus, a quite intriguing research question is: What kind of strategies do people use in the process of integrating indicators of morality in a particular moral situation into an overall moral evaluation of the human activity that is assessed?

Are human activity and events co-extensive? Are their morally significant events that are not human activity?

This quite novel approach to the evaluation of moral reasoning processes is proposed by J. Grác (2008, 2015), who has made a conceptual contribution towards the psychological

analysis of the interiorization processes of moral norms. It is worthy of note that Grác in his original Slovak work (2008) distinguishes between the terms: *mravnost'*, *moralnost'* and *etika*. In the *Introduction to the Polish edition* of Grác's book, Biela (2015) emphasizes that these three terms only exist in discriminative usage in two contemporary languages, i.e. Czech and Slovak, and the term *mravnost'* is no longer used in Slavic languages other than Czech and Slovak. This certainly means that psychologically, the users of these two languages operate with more developed cognitive and behavioral structures for the moral domain than the users of other European languages.

Certainly? This is not clear. And moreover, you do not explain what these terms mean, and why they may be important for others.

What is the psychological novelty of Grác's conception of morality, and what is its methodological significance for an experimental approach to moral reasoning processes? Its greatest novelty is in extending the conditions of assessment of human performance in the defined situation as morally good or bad. In order to define the criteria enabling such an evaluation, Grác distinguished two parallel analytical dimensions of human performance: (1) moral behavior, i.e. the external revealing of moral decision making (external dimension); (2) the internal experience related to the moral behavior, i.e. intentional focus on the moral norm, motivation (internal dimension). In this way, the psychological understanding of moral norms as factors regulating human performance is enhanced by experiential and behavioral structures, creating a basis for systematizing norms as moral, executive, and legal. Executive and legal norms regulate only externally revealed human performance. The characteristic of moral norms is that they regulate both human behavior associated with internal experiences focused on a particular moral norm as well as these internal experiences themselves (i.e. internal experience not tied with revealed behavior (Grác, 2008).

This is not clear. Where is this norm located? How does it exert its influence?.

In this context, we can evaluate an individuals' ethical conduct in a specific situation as being a morally good action or a morally bad one. However, *morally good* vs. *morally bad*

Now, this use of "moral" is sensible, as a distinction from what may be cnsidered the "ethical" or social, here your "external" However, it is not clear that Grac is originally responsible for such a distinction, and still, the three special terms have not been clarified.

designates human behavior in a concrete situation, independent of whether the specific moral norm, e.g. hard work, courage, honesty, respect, trustworthiness, dedication, loyalty, has been actualized or not. Grác (2008) analysis posits that a person who accepts a particular moral norm but does not meet it could not be evaluated as performing a morally good action. And, similarly, a person could not be evaluated as having performed a morally good action if that person behaved in a manner consistent with the specific moral norm yet he or she did not accept this norm. Only the conjunction of the positive internal experience of the moral norm with the positive fulfillment of this norm in the situation provides the necessary and sufficient conditions for the positive ethical evaluation of a person acting in a given situation.

The mental processes involved in ethical evaluation of moral performance in everyday life situations and, particularly, in extreme situations are not passive ones on the part of the human subject; they involve active interaction with the social and natural environment.

These processes are truly creative in their nature, as they transform the human life environment into a valuable system of values, called an axiological system. These processes do not present a simple or easy reality for psychological research, or for the experimental approach in particular.

MATERIALS AND METHODS

Moral reasoning implies two sets of mental processes related to moral norms. The first set is related to the mental interiorization of moral norms, and the second set deals with the regulative function of moral norms in an individual's personal life, social interactions, and the natural environment. These mental processes create real-life moral

As your study relies on the work of Grac, the preceding discussion should set this theory out in greater detail. Rather, now the methods section begins by assuming that this is an adequate theory, with which the reader is adequately acquainted. However, the introductory pages are deficient in this regard.

situations, where moral norms interact with executive and legislative norms. Grác (2008, 2015) presents the internormative extensional relations between the executive, moral, and legislative norms using Venn diagrams (Venn, 1884). See Figure 1.

Mental process "create" "real-life moral situations"?

Figure 1. The internormative extensional relations between the executive, moral and legislative norms.

The Venn diagram illustrated in Figure 1 demonstrates that if the logical point of view is taken into consideration, we can see the relations between the classes of norms: executive (E), moral (M), and legislative (L); thus we can dislocate the extensional connections into the seven separate subclasses of norms. If we denote the general class of norm as CN, and the symbol ∩ denotes logical conjunction, the mutual relations presented in Figure 1 can be expressed in Formula (1), respectively:

$$CN = \{E, M, L: E \cap M, L \cap M, E \cap M \cap L\} \qquad (1)$$

If this is to say that everything is moral, then I might agree.

As moral norms will be the main point of reference in our experimental approach, we will now pay more attention to the subclasses from Formula (1) which contain only such norms. The first such subclass, symbolized as $\dot{M}$, denotes the moral norms which belong only to the universe of moral norms but which do not belong to executive or legislative norms. As such, and where *x* symbolizes any moral norm, and ε is a predicator denoting *belonging to*, this subclass may be strictly defined as in Formula (2):

$$\dot{M} = \{x: x \in M \cap \{x \notin E \cap x \notin L\}\} \qquad (2)$$

The second two subclasses are the conjunctions of moral norms with legislative norms: M∩L and moral norms with executive norms: E∩M. The appropriate formal explications can be viewed in Formulas (3) and (4), respectively:

$$M \cap L = \{x: x \in M \cap L\} \qquad (3)$$

$$M \cap E = \{x: x \in M \cap E\} \qquad (4)$$

There is also a subclass of moral norms in Formula (1) which is a conjunction of the three norms, executive, moral, and legislative: $E \cap M \cap L$, which is expressed in Formula (5) as:

$$E \cap M \cap L = \{x: x \in E \cap M \cap L\} \qquad (5)$$

The moral norms presented above will be employed in our experiment in the context of moral situation referred to as *ethical stories* or briefly, as *moralities.* This method has already been employed in psychological research on moral issues, e.g. by Piaget (1976) and Kohlberg (1976) to diagnose the moral development of children and youth.

What did they do that you do, using "this method" exactly? Limited to "youth" of what age? How does your study differ?

It is worth reviewing the strong and weak aspects of using *ethical stories* in psychological research. We propose conducting a short SWOT analysis of *moralities* in empirical research to confront this method with personal experience or observations of real life moral situations. The results of this analysis are collected in Table 1.

Table 1. The outcome of SWOT comparative analysis outcomes of direct observation of real life moral situation and ethical stories (moralities).

Methods / Evaluation	Personal experience or direct observation of a moral situation	Ethical stories or moralities
Strong sides of the method	1. Acknowledges the rich context of a moral situation.	1. Enables grasping *hic et nunc* the ethical essence of the moral

	2. Enables employing an idiographic analysis of ethical evaluations of human behavior.	situation.<br>2. Enables employing nomothetic oriented research in ethical evaluation of human behavior.
Weak sides of the method	In many moral situations the moral motives and intentions are hidden to the external observer.	Abstracts from the entire context of the moral situation.

From the outcomes of SWOT analysis we learn that ethical stories are more easily employed in nomothetic empirical analysis on moral situations. It must be stressed clearly, however, that the subject of our research will be the cognitive aspect of ethical evaluation related to the concept of *morally good performance in moralities* analysis. Cognitive schemas of ethical stories offer the keys to understanding the concept of internalized behavior.

This last paragraph is very rough. Some discussion of the analysis may be beneficial.

Cognitive schemas of ethical stories

Did you have some passage from Kant in mind, here, or…?

Let us review the main concepts related to ethical stories. Indicators of moral context relate to analysis of cognitive schemas of ethical stories. One indicator is a *moral requirement* or *demand* to do something in a defined situation, which is usually expressed by employing the functor of assertive judgement *should* that was systematically elaborated by Immanuel Kant as a *categorical imperative*. Without this indicator, none of the moral norms defined in Formulas (1)-(5) could exist at all. If we state that the symbols *CI* denote Categorical Imperative (i.e. the experience in a defined situation that *I should do something*

This doesn't sound much like Kant's CI.

Does Kant base his CI in personhood?

which I understand is morally good for some person or persons), we can conclude that the norm regulating my behavior is moral. This moral condition can be expressed as an alternative connection of the particular subsets of moral norms defined in the appropriate Formulas (2)-(5):

$$x \in CI \Rightarrow \{(x \in M \cap x \notin E \cap x \notin L) \vee (x \in M \cap L \vee x \in M \cap E) \vee (x \in E \cap M \cap L)\} \quad (6)$$

Another indicator is the object or moral regulation, which is understood as a content or domain regulated by the particular moral norms indicated in (6). Morally good performance assumes a specific object within which a particular moral norm has been actualized. Good examples could be the ethical codes of various professions. It is precisely

"precisely stated" - You might cite and perhaps quote relevant examples.

stated in such codes that particular content-oriented professional activities are extracted from the remaining professional activities to be regulated by moral norms.

Also belonging to the indicators of morality are what causes a moral norm to become the given norm of the content of a the concrete ethical story, that is, what specifically distinguishes the given norm (specified in Formula (6)) from any other outside normative moral activity of a human being (as regulated by legislative – L, or executive – E norm, as seen in Figure 1 and in Formula (1)). Such particular indicators could be, e.g. *intention* (motivation, plane, thought, idea, design), *concordance* (acceptation, assent, agreement), *application* (adoption, appliance, adhibition), *outcome* (result, consequence, effect, coherence, consistency), *knowledge* (cognition, learning, recognition, study), realization (implementation, accomplishment, fruition, completion), etc.

In our analysis we will denote the particular indicators of morality by the first letters of their names, as follows: intention – I; concordance – C; application – A; outcome – O; knowledge – K; realization – R. The above indicators, as we shall later see, have unequal regulative strength, which means that in a given ethical story one indicator could be

exchanged by another one or, in some cases, might not even be taken into consideration at all. Examples of indicators of weaker regulative status could be: Knowledge (K) or Outcome (O).

Why would these be “weaker”?

After reviewing the literature on indicators of morality (Moore, 1908; Nikolaičeva,

Though there is some semblance of a review, there is no systematic exposure of “indicators”

1976; Piaget, 1976; Kohlberg, 1976; Grác, 2008, 2015), we decided to apply the following six indicators for our experimental design: intention – I; concordance – C; application – A;

Are these lifted from those resources, or are they products of your assay, or…?

outcome – E; knowledge – K; and realization – R. Realizing that moral reasoning related to ethical evaluation of human behavior in real-life situations is definitely a complicated mental process, we have decided to restructure the above indicators into complementary experimental units, in the two cognitive schemes expressed formally as:

$$IAR = I \cap A \cap O \qquad (7)$$

$$KCA = K \cap C \cap R \qquad (8)$$

The issue is: what values may the indicators in the Formulas (7) and (8) take? It is not a problem to apply the mathematical symbols (+) and (-) to denote, by analogy, an evaluation of human behavior in the ethical story as morally good or bad, respectively. However, these two bipolar values are not sufficient for moral evaluation of ethical stories, because in these stories we can meet with human behavior which, for various reasons, is beyond moral evaluation. That is why we have introduced a third neutral value that may be assigned to each indicator employed in our experiment. The symbol Ø taken from Boolean algebra is applied here. This value will denote that in terms of the specific indicator, the analyzed human performance is outside or beyond the specific aspect of moral evaluation. Moreover, it has to be emphasized here that in the cognitive schemas constructed in this experiment, the denotation of each indicator of morality is precisely defined not only by

marking it as (+), (-) or Ø, but also by giving a particular interpretation to it in respect to the specific moral dimensions in these schemas. We term these specific interpretations as *definiens*, which will be precisely defined for the particular schemas (see Chart 2 and Chart 3). Next we present the cognitive schemas of IAO and KCR as the matrices for this experiment on moral reasoning.

Advice: recompose the first 15 pages as 8 or 10, and better introduce Grac and this approach in the same.

IAO cognitive schemas

Indicators for the moral assessment and their definiens, i.e. criteria for their evaluation are defined in Table 2.

Table 2. The indicators of morality aspects and their definiens, i.e. criteria for their ethical assessment in the IAO schemas.

Indicators of morality aspects	Criteria for their ethical assessment		
**I**NTENTION– the behavior intention, incentive and the moral norm are:	(+) in accord	(-) in discord	(∅) neither
**A**PPLICATION – the form applied in this particular case in relation to the moral norm:	(+) corresponds	(-) does not correspond	(∅) neither
**O**UTCOME **–** consequence accompanying, possible outcome is:	(+) positive	(-) negative	(∅) neither

These indicators are specific conditions under which any moral norm works in a certain life situation. The criteria are definientia (sing. *definiens*) of the moral norm effect, expressing the fact that each of the perpendicularly identical symbols (+ - Ø) has a different notional content – depending on its combination with the indicators.

The first indicator of the IAO schema is Intention – I. It is understood as a motive or intent of the individual's ethical behavior. As an individual cannot be forced to be moral, the axiological principle in ethics assumes his or her deliberation and free decision-making in the moral norm interactions. For this reason, this indicator is regarded as exceptionally important. But the indicator of the person's motive or intention cannot be absolutized since the individual's conscious relationship to a moral norm is an inevitable but not sufficient precondition for an ethics-based assessment of the person's behavior. How the concrete behavior will be evaluated from the perspective of intention and motivation depends on the criterion definientia belonging to this indicator. If, for example, the motive or intention of the behavioral event is in accord with the moral norm, then the result of the partial evaluation of behavior is positive (+). If it is in discord, the result is negative (-). The symbol Ø (*nullus*) signals that neither of the previous assessments are valid. Usually, this refers to activities which, due to their content, are regarded as beyond moral, but also to those where the motive of behavior is regulated by a moral norm, but the person is not aware of it.

Application – A, the second indicator of the IAR scheme, answers the question of whether the intention which is moral is or is not obtained by moral means. If the means of the intent's actualization corresponds with the moral norm, the result is positive (+). If the person tried to achieve the intended good by unethical means, their behavior is evaluated negatively (-). This means that the application indicator always assumes re-activation of the original intent and thus there is also the possibility of a greater or smaller change in the

application process; consequently, this resultant motive may, but also may not, be in accordance with the original motive. The fact that every application implies not only motivation but also its actualization gives this indicator, according to the IAO scheme, a crucial position in the evaluation of the individual's ethical behavior.

The third indicator of the IAO scheme is Outcome – O, as a possible or an accompanying outcome. The indicator in question is related to the assessed behavioral event. The concept of relation itself can be understood as causal or non-causal. It could be causal in the case where the intention indicator was the cause of the actualized intention and the consequence indicator was the impact of it. Since the function of the actualized intention was already adopted in the IAO scheme by the intentional indicator (as mentioned above), it is not possible – in the framework of the same scheme – to assign it simultaneously to the third indicator. Therefore, the Outcome indicator can be understood in the given scheme exclusively as non-causal, i.e. as a consequential circumstance which is related to cause and effect, but which cannot be identified with it. From the above-mentioned analysis, it follows that the assessment of the person's behavior by means of three signs (+ - Ø) applies only to the evaluation of the accompanying consequence *per se* and does not influence the overall evaluation of the individual's behavior in the given moral situation.

For the above reasons, the ethical evaluation processes in a moral situation within the cognitive scheme of IAO could be formulated as follows (9):

$$\bigwedge_{I,A,O} \bigvee_{\{x:x\in\{(+),(-),\emptyset\}\}} = \begin{Bmatrix} (I=(+) \vee I=(-) \vee I=\emptyset) \cap (\mathrm{A}=(+) \vee \mathrm{A}=(-) \vee \mathrm{A}=\emptyset) \cap \\ (\mathrm{O}=(+) \vee \mathrm{O}=(-) \vee \mathrm{O}=\emptyset) \end{Bmatrix}$$

KCR cognitive schemas

Indicators of moral assessment related to the cognitive schemas KCR and the criteria for their evaluation are presented (so called *definientia*) in Table 3.

Table 3. The indicators of morality aspects and their definiens, i.e. criteria for their ethical assessment in KCR schemas.

Indicators of morality aspects	Criteria for their ethical assessment		
**K**nowledge – as a cognitive component to the specific moral norm	(+) exists	(-) doesn't exist	(∅) neither
**C**oncordance – acceptance of the particular moral norm	(+) is accepted	(-) is not accepted	(∅) neither
**R**ealizing **–** the moral norm has actually been implemented	(+) fulfilled	(-) not fulfilled	(∅) neither

The first indicator of morality in the KCR schema is Knowledge about moral norms, which regulates human behavior in a given situation. This is really moral knowledge about the specific norm, which can exist as a socially accepted norm within public opinion, regulating interpersonal relations. However, the acting person may or may not have knowledge about this opinion. Thus, the cognition of the moral norm can be very differentiated. The norm can exist as a socially accepted ethical obligation which a concrete individual might not know about. There could also be cases where an individual has a positive attitude toward the defined requirement and accepts it as his or her own moral norm, independently of whether such norm objectively exists as a social standard or not.

The symbol Ø can be used to value this indicator, if is really unclear as to whether to assign a value (+) or (-).

The second indicator in KCR is Concordance (C) of the person interacting in a given moral situation. Concordance means the actor's personal attitude to the particular moral norm regulating human performance in this situation. According to Grác (2008, 2015), using the signs: (+) or (-) in valuing the indicator C is relatively univocal, but some problems concerning understanding may require the sign Ø. This sign should be employed if the evaluating person has neither a positive nor negative attitude towards the given moral norm or when this person does not have his or her own understanding of the moral requirements related to the norm in question.

The last indicator Realizing – R refers to whether and how the requirements of the moral norm regulating human behavior are fulfilled. A positive (+) value of this indicator signifies that implementation of the moral norm is fulfilled. This indicator receives a negative value if implementation of the moral norm is not fulfilled in a situation where it is supposed to be. Ethical evaluation faces greater difficulty when considering whether to assign the value Ø. This case is particularly difficult to assign due to the fact that there is no analogy between a neural attitude towards a moral norm (as in the case of indicator C above) and the neutrality of acting in a concrete situation, when we are dealing with human performance.

In concluding this stage of our analysis, we can state that ethical evaluation processes in a moral-content-situation within the cognitive scheme of KCR can be formulated as follows (10):

$$\bigwedge_{K,C,R} \bigvee_{\{x:x\in\{(+),(-),\emptyset\}\}} = \begin{Bmatrix} (K = (+) \vee K = (-) \vee R = \emptyset) \cap (\mathrm{C} = (+) \vee \mathrm{C} = (-) \vee \mathrm{C} = \emptyset) \cap \\ (\mathrm{R} = (+) \vee \mathrm{R} = (-) \vee \mathrm{R} = \emptyset) \end{Bmatrix}$$

Control of independent variables in evaluating cognitive schemas of ethical story experiments

The main methodological requirement for any experimental condition is to control the independent variables which make an impact on the dependent variable. In our experiment on moral thinking, the main dependent variable to be explained is the ethical evaluation of human behavior in terms of moral values. It is assumed that moral reasoning is a structured mental process like any other human thinking structured towards problem solving. Since John Dewey's *How we think* (Dewey, 1910), psychologists working on problem solving have distinguished the phases of thinking which lead human thinking to reach the goal of solving a problem. Similar thinking takes place in a real-life moral situation where a

Do we have a lot of time to consider right and wrong in most real-life moral situations? Some? All?

human being considers whether and which action should be taken in order to behave in a morally acceptable way, in accordance with his or her conscience – based on a moral norm.

Our experiment will be based on the evaluation of ethical stories (moralities), using the defined structured indicators of morality, called cognitive schemas of the ethical-

the novelty of the experimental design may be discussed during discussion after the results are interpreted, and when the contribution to the field may be assessed, typically right before the concluding section.

dilemma-stories, described above as IAO and KCR.

The first novelty of this experimental approach is the two-phase design : (1) modeling of ethic-dilemma stories based on logically structured cognitive schemas, and (2) overall ethical evaluation of the modeled morality schemas.

Assessing the effect of any moral norm in conditions of an abstract situation given by the schemas IAO and KCR is one methodological challenge; another is grasping these conditions in a person's actual behavior. This can be done by means of modeling written

stories which present moral dilemmas. These stories enable the observer to examine intrinsically experienced behavior, e.g. their motivation or intention, which is usually hidden from a second party's retrospection.

We use the term *modeling* for the creation of moral dilemma stories. This involves the assignment of arbitrary content of actually displayed behavior to the respective value indicators of the schema, so that this allocation may accurately correspond to the criterion definientia of the indicators (I,A,O,K,C,R) of morality. Educational psychology students were the subjects of this natural experiment. They were asked to create model ethical stories concerning moral situations of young students of their age, according to carefully-prepared instructions informing them how to do this task.

This is page 28.

Control of independent variables in the experiment

Three question arose in the definition of *modeling*. The first of these is formulated as follows: how many *independent variables*, that is, different configurations of signs, are offered for story modeling by the schemas IAO and KCR, respectively?

In order to answer this question for IAO schemas we have to strictly consider the three indicators of morality where each of them, formally speaking, may take three possible values: (+), (-), Ø. Thus, to calculate all configurations of each indicator combinatorically, where generally its number is *n,* where the number of values that each of the indicators can take may be generally symbolized by *r*, we could apply the formula for variations with repetition n^r. This means that variations with *n* elements of the rth class are all possible configurations of *n* indicators of morality and the *r* values which each indicator can take. In our case, where the number of indicators *n*=3 in the IAO schemas, and the number of values

which each of them can take is r=3, the number of all configurations is simply attained using Formula (11) as follows:

$$\{N(IAO){:}\,N(IM) = 3\ \cap N(VIM) = 3\} \Rightarrow \{N(IAO) = 3^3 = 27\} \qquad (11)$$

where:

N(IAO) – the total number of independent variable configurations related to the indicators of morality and their values in the IAO schemas;

N(IM) – the number of indicators of morality in the IAO schemas;

N(VIM) – the number of values that can be taken by each of the indicators of morality in the IAO schemas.

And, similarly, for the KCR schemas, where the number of indicators is also *n*=3, and the number of values which each of them can take is *r*=3 as well, we can reach the number of all configurations simply by using Formula (12) as follows:

$$\{N(KCR){:}\,N(IM) = 3\ \cap N(VIM) = 3\} \Rightarrow \{N(KCR) = 3^3 = 27\} \qquad (12)$$

this is interesting, simple, but why is it coming so late? It seems that this series of rather simple statements should/could have been set out in a matter of a couple of pages, if necessary at all. The main concern here is that discussion is spread out, and loses focus. Is this an experimental report, interpreting results? Or, is this something else?

where:

N(KCR) – the total number of independent variable configurations related with the indicators of morality and their values in the KCR schemas;

N(IM) and N(VIM) – are as in Formula (11).

Is this a new section? Subsection?

The elementary experimental fields of moral evaluation: ethical stories

The second question is whether the same story content, which is modeled according to certain specific criteria of evaluation, can be *modified* in accord with further randomly applied evaluation criteria of the IAO or KCR schemas. In an effort to answer this question, we can alternately replace each indicator of morality by one of the criterion values: (+), (-), Ø. If we take as an example the description of three 18-years-old youths who solved the

problem of moving to a new place to live in different ways, we obtain the IAO and KCR schema matrices with nine boxes as a part of 27 total number of combinatorial boxes, accounted separately for the IAO and KCR cognitive schemas, as presented in Formulas (11) and (12), respectively. For the schema matrixed in our experiment the total number of rows i=3, and the number of columns is c=3. If any line is denoted as i and any column as j, then any field in these matrices can be expressed as an experimental field EF_{ij} of partial moral evaluation of human performance in ethical story. An example of such EF_{ij} are shown in Table 4 for the IAO schemas.

Table 4. Matrix of IAO schemas illustrating the elementary experimental fields of moral evaluation in ethical stories.

columns / rows	c=1	c=2	c=3
i=1	**I (+)** John found a new flat to create better conditions for his siblings to study.	**A (-)** He did it regardless of the disapproval of his mother, who felt almost mortally hurt by his decision.	**O (∅)** After having moved to his new flat John found out that the signal for his cell phone was the same quality as in the previous location.
i=2	**I (-)** Paul found a new flat to have easier access to	**A (∅)** While moving to the new flat, he decided to travel by	**O (+)** At the new location he met a neighbor who

	drug dealers.	train instead by bus.	began to exert a positive influence on him.
i=3	**I (∅)** Peter found a new flat because he wanted to live alone.	**A (+)** By mutual consent with his parents he left their home.	**O (-)** While moving to the new flat he slipped and broke his leg.

These are outcomes with some moral significance, and clever, but are they moral/ethical stories?

As in our case, the IAO scheme is a square matrix, the values in the first line can be written symbolically: $i_1\ c_1$ = I (+), $i_1\ c_2$ = A (-), $i_1\ c_3$ = O (∅) and at the same time they can be checked by reading them verbally in the first row of the chart. The square matrix enables us to read the same story plot variably, i.e. according to an arbitrary configuration of boxes in the lines, without losing the inner logic (meaning). For example, if we select only positive criteria, then the story plot can be expressed in the following notation: $i_1\ c_1$ = I (+), $i_3\ c_2$ = A (+), $i_2\ c_3$ = O (+). When we read it in the chart in this transcript, we find out that the story plot did not lose its inner coherence. It is possible to symbolically record only the minus or only the null configuration in the same way. However, any random two-element combinations are also possible, e.g. combination of two negative and one positive value. In that case, the notation is as follows: $i_2\ c_1$ = I (-) , $i_1\ c_2$ = A (-), $i_2\ c_3$ = O (+).

Based on the above, the IAO schemas make it possible not only to create stories differing in their content, but they also offer the opportunity to modify the already-modeled stories.

this part about manipulating stories through manipulation of the matrix is interesting.

In a similar way, we will now give an example of experimental fields EF_{ij} of the schemas KCR shown in the square matrix presented in Table 5.

Table 5. Matrix of the KCR schemas illustrating the elementary experimental fields (EF_{ij}) of moral evaluation in ethical stories.

columns / lines	*c*=1	*c*=2	*c*=3
i=1	**K (+)** Igor knew that cruelty to animals is immoral.	**C (-)** He was not opposed to ill-treatment of animals.	**R (∅)** However, he has never even been in a situation where he watched cruelty inflicted on animals by others.
i=2	**K (-)** Andrew did not know that cruelty to animals is immoral.	**C (∅)** In view of the abuse of animals, he was unclear and of a contradictory position.	**R (+)** Yesterday, when his colleagues mistreated a dog, he protected it.
i=3	**K (∅)** Emil was not sure if cruelty to animals is moral or immoral.	**C (+)** But personally he did not agree with the abuse of animals.	**R (-)** However, yesterday, when colleagues mistreated a dog, he was

			also cruel to it.

From Table 5 we can see that KCR schemas, similar to IAO schemas, also have a variable structure of stimuli constituted by the content of combinatorial sequences of elementary experimental fields (EF_{ij}) at a level equal to IAO. These experimental fields are indicators of morality of each individual ethical story presented in both the IAO and KCR schemas. The experimental control of independent variables in this experiment deals with combinatorial control of the particular scheme in terms of the formal values (+), (-) and $\varnothing$ in IAO and KCR, respectively. Moreover, in order to avoid contradictions between the concrete schemas, on the one hand, and to maintain individual and personal coherence of the ethical story content, on the other, the subjects were the heroes of each of the 27 IAO schemas and 27 KCR schemas, where each schema actor was assigned a different name in the modeled ethical stories.

Experimental design

In our experiment the stimuli are the individual schemas, which consist of three indicators of morality ordered in the two following sequences: IAO and KCR, where each of the indicators takes only one of three values in its matrix of sequences (see Table 4 and Table 5). The valuated indicators of morality constitute a concrete cognitive schema as a complex sequential stimulus which consists of three particular experimental fields as the objects of ethical individual story modeling in the first phase of the experiment.

Thus, each individual IAO schema can be defined as a well-ordered set of three:

$$Si(\mathrm{IAO}) \stackrel{\mathrm{def}}{=} \langle I, A, O \rangle \qquad (13),$$

where: S_i(IAO) - any of the *n* (*n*=27 in our experiment) IAO schemas; I={(+),(-),∅}**;** A={(+),(-),∅}; O={(+),(-),∅} **–** denote the sets of values which can take particular indicators of morality in the IAO schemas. This indicates that the order of the indicators constituting the IAO schema should not be changed.

In a similar way, each individual KCR schema can be defined as a well-ordered set of three:

$$Si(KCR) \stackrel{\text{def}}{=} \langle K, C, R \rangle \qquad (14),$$

where: S_i(KCR) - any of n (n=27 in our experiment) KCR schemas; K{(+),(-),∅}; C={(+),(-),∅}; R={(+),(-),∅} – denote the sets of values which can take the particular indicators of morality of the KCR schemas.

The methodological analysis conducted allows us to formulate an experimental design to present cognitive schemas for ethical story modeling by educational psychology students. These ethical stories involve moral issues from the students' own community. This design is included in Table 6 as a matrix of formal schemas numbered from 1 to 54 and presented in parallel columns in such a way that numbers for schemas IAO and KCR are located on either side of the formal valuation structures of the indicators of morality.

Table 6. The experimental design: the structure of valuation of indicators in particular IAO and KCR cognitive schemas for experimental modeling of ethical stories by educational psychology students.

IAO schemas	Modeling formal patterns	KCR schemas	IAO schemas	Modeling formal patterns	KCR schemas	IAO schemas	Modeling formal patterns	KCR schemas

1	(+ + +)	28	10	(∅ **- -)**	37	19	(- ∅ **+)**	46
2	(+ - +)	29	11	(- - ∅**)**	38	20	(+ - ∅**)**	47
3	(- + +)	30	12	(∅∅∅**)**	39	21	(+ ∅ **-)**	48
4	(+ + -)	31	13	(- - +)	40	22	(∅ **-** ∅**)**	49
5	(+ ∅ **+)**	32	14	(- + -)	41	23	(∅∅ **-)**	50
6	(∅ **+ +)**	33	15	(+ - -)	42	24	(- ∅∅**)**	51
7	(+ + ∅**)**	34	16	(∅ **+ -)**	43	25	(∅∅ **+)**	52
8	(- - -)	35	17	(+ - ∅**)**	44	26	(∅ **+** ∅**)**	53
9	(- ∅ **-)**	36	18	(∅ **- +)**	45	27	(+ ∅∅**)**	54

The order number of formal valuations of the IAO schema is always on the left side of the appropriate column for modeling formal patterns in Table 6 and the order number for the KCR schema is on the right side of this column, respectively. The signs of moral evaluation should be read from left to right both for IAO and KCR schemas.

From Table 6 we learn that both cognitive schema enable the planned experiment on moral thinking to control for variability of stimuli structure via evaluated indicators of morality of human performance modeled in quasi-natural experimental conditions during the students' classes.

Subjects, experiment, modeling instructions and procedure, and overall moral evaluation of ethical stories

The Subjects

One hundred thirty-four third-year educational psychology students of a modal age of 20 years participated in our experiment on the modeling of ethical stories as part of a regular course on educational psychology at the Faculty of Philosophy of Trnava University in Trnava (Slovakia) over the years 1998-2000. 80% of the subjects were female students and 20% were males. After participating in theoretical lectures on cognitive schemas of ethical stories and evaluation of related indicators of morality, the students of the seminar on educational psychology were presented with the following task in accordance with these short instructions:

Experiment instructions and procedure

this is the purpose of the paper, to set out the results of this exercise. But, it appears in any detail on page 30… this is a problem.

As you have learned today from the lecture on education psychology, each schema of an ethical story has its own order number in our experimental matrix and a concrete valuation of its three indicators of morality. Now you will receive data about two IAO schemas and two KCR schemas. Your task will be to creatively model the thinking and performance of a 17-year-old individual, different in each of the projected stories, in accordance with the given values of the individual indicators of morality according to the patterns of potential schemas, the order numbers of which will be generated by random number generator.

After this instruction each individual subject was given the *Ethical Story Modeling Experiment Report Form* (see the form with this protocol in the Appendix) containing four order numbers associated with indicator values for potential ethical stories generated from Chart 6 by a random number generator. The subject was instructed about where and what

the modeling schema of the concrete story should be in accordance to the experimental design, and that the hero of each story should be an individual person known by his or her name.

Once the modeling phase of the experiment was completed, in the next phase the experimenter asked the subject to conduct an overall evaluation of each of the constructed ethical stories in his or her protocol as a whole by assigning a global symbol in square brackets [ ] to denote their overall assessment of moral values of human performance as: morally good [+], morally bad [-] or morally neutral [∅].

The experimental modeling procedure took place in a seminar room where the subjects could model the educational stories in quiet and comfortable conditions. They conducted their experimental modeling individually, sitting separately at seminar tables in small groups. The average time for modeling was about 45 minutes. After completing the story modeling and conducting the overall evaluation of each of the constructed ethical stories, the students gave the completed *Protocol for Experimental Modeling of Ethical Stories* and their completed *Moral Evaluation* back to the experimenter (see: APPENDIX 1).

RESULTS

The research findings from this moral reasoning experiment are presented according to the two phases of this experiment: (1) the results of modeling ethical stories, and (2) the results of the overall moral evaluation of the modeled ethical stories (see: the example of the *Protocol of Experimental Modeling of Ethical Stories* and its completed *Moral Evaluation* back to the experimenter (see: APPENDIX 2) .

The modeling phase

The total number of protocols received with completed modeling was 536. This means that the main result of our findings from the modeling phase of our experiment on moral reasoning is a systematized empirical data base of ethical stories, which is a kind of empirical description of characteristics of denotative meaning of the indicators of morality understood in their positive, negative, or neutral meaning in a context of cognitive schemas of ethical stories. This base is systematized in accordance with the order numbers of the 54 IAO and KCR cognitive schemas, sequenced and presented as a matrix in Table 6. One way of presenting this data base to list the modal examples of modeling created on the base of this matrix. The full list of modal models can be found in the original Slovakian version of the report by Grác (2008, pp 119-125) and the Polish version by Grác (2015, pp.140-148).

This data base can be a source for qualitative analysis on the structuring of moral reasoning using indicators of morality. One of the opportunities for research using this data base points is to use the schemas for research on overall assessment of the moral value of the ethical stories. This is what was done in the second phase of this research project.

The moral evaluation phase

We will now show what kind of cognitive strategies the subjects developed in their overall moral evaluations, based on analyzing the values of indicators of morality as premises in inferring the conclusions in the IAO and then KCR schemas.

Strategies used to complete overall moral evaluations of the IAO schemas of ethical stories

The first main finding concerning the strategies used by students to conduct overall moral evaluations of the IAO schemas of ethical stories is that the indicator of outcomes (O) of ethical stories did not have any impact on the overall evaluation of these stories. This

means that for the subjects of the study, this indicator did not determine their moral evaluation of ethical stories, but only offered a situational context for these stories.

The students' overall moral evaluations of the IAO ethical stories allowed us to identify three regularities which describe their moral reasoning in analyzing the values of the indicators of morality in these stories. The first regularity can be called the *principle of univocal values of intention and application indicators*. The matrix which indicates the overall moral evaluations which fit this regularity is shown in Table 7.

Table 7. Global moral evaluations of the IAO schemas in accordance with the principle of univocal values of intention and application indicators

Order number of the schema	IAO schema	Overall moral evaluation	Order number of the schema	IAO schema	Overall moral evaluation
1	(+ + +)	[+]	12	(∅∅∅)	[∅]
4	(+ + -)	[+]	13	(- - +)	[-]
7	(+ + ∅)	[+]	23	(∅∅ -)	[∅]
8	(- - -)	[-]	25	(∅∅ +)	[∅]
11	(- - ∅)	[-]			

The analysis of Table 7 allows us to formulate *a principle of univocal values of intention and application indicators* as follows:

$$(I = A) \Rightarrow (ES = I = A) \qquad (15),$$

where: ES denotes the overall moral evaluation of ethical story; I and A denote the appropriate signs of moral values of the indicators in IAO schemas.

The second regularity of the overall evaluation of the IAO schemas of ethical stories is called the *principle of preferring application (A) indicator over intention indicator (I)*. Table 8 shows the matrix indicating the overall moral evaluations which fit this principle.

Table 8. Overall moral evaluations of IAO schemas in accordance with the principle of preferring application (A) indicator over intention indicator (I).

Order number of the schema	IAO schema	Overall moral evaluation	Order number of the schema	IAO schema	Overall moral evaluation
2	(+ - +)	[-]	16	**(∅ + -)**	[+]
3	(- + +)	[+]	17	(- + **∅)**	[+]
6	**(∅ + +)**	[+]	18	**(∅ - +)**	[-]
10	**(∅ - -)**	[-]	20	(+ - **∅)**	[-]
14	(- + -)	[+]	22	**(∅ - ∅)**	[-]
15	(+ - -)	[-]	26	**(∅ + ∅)**	[+]

From Table 8 we can learn that *the principle of preferring application (A) indicator over intention indicator (I)* may be formally expressed as:

$$(I \neq A) \Rightarrow (ES = A) \quad (16),$$

where the symbols denote the same as in (15).

The third regularity says that if the intention indicator is non-zero, and the sign of the application indicator is zero, the concluded overall moral evaluation of the ethical story is formally expressed by the sign of the intention indicator, independently of the sign of the outcome indicator. This regularity is called as the *principle of preferring the intention indicator (I) over the non-zero application indicator (A)*. The designates of this principle are shown in the matrix presented in Table 9.

Table 9. Overall moral evaluations of IAO schemas in accordance with the principle of preferring the intention indicator (I) over application (A) indicator.

Order number of the schema	IAO schema	Concluded moral evaluation	Order number of the schema	IAO schema	Concluded moral evaluation
5	(+∅ +)	[+]	21	(+∅ **-)**	[+]
9	(-∅ **-)**	[-]	24	(-∅**∅)**	[-]
19	(-∅ **+)**	[-]	27	(+∅**∅)**	[+]

Analysis of the patterns of indicator signs allows us to formulate in a formal way the principle of preferring the intention indicator (I) over the application indicator (A) as follows:

$$[(I \neq \varnothing) \cap (\mathbf{A} = \varnothing)] \Rightarrow (ES = I) \quad (17).$$

Strategies used to complete overall moral evaluations of the KCR schemas of ethical stories

The first main finding concluding the moral evaluation of the ethical stories within the KCR schemas is that only concordance (C) and realization (R) indicators of morality played a decisive role in the overall moral evaluations of the stories. Familiarity with the heroes of the stories of moral norms (K) appeared only to play a contextual role in the ethical stories, with no impact on completing the moral evaluation process.

The students' assessment data allowed us to identify the following four strategies in concluding the moral evaluations of the KCR schemas of ethical stories:

1. If the sign of the concordance indicator (C) is univocal with the sign of the realization indicator (R), the sign of the concluded moral evaluation of the ethical story is equal to the signs of both indicators in the premises of this conclusion, which can be formally expressed as:

$$(C = R) \Rightarrow (ES = C = R) \qquad (18),$$

where: ES – denotes the same as in the above formulas, and C, R denote the symbols of the KCR schemas.

2. If the sign of the concordance indicator (C) contradicts the sign of realization indicator (R), the overall moral evaluation is expressed with the sign ∅**.** As a formal expression it is stated as:

$$(I \neq R) \Rightarrow (ES = \varnothing) \qquad (19)$$

3. If the sign of the concordance indicator (C) is neutral (∅),the overall concluded moral evaluation of the ethical story is neutral as well, which one can formulate as:

$$(C = \varnothing) \Rightarrow (ES = \varnothing) \qquad (20)$$

4. If the sign of the realization indicator (R) is neutral (∅),the overall moral evaluation of the ethical story takes its value from the non-zero evaluation of the concordance indicator (C). In a formal way this regularity may be expressed as:

$$(R = \varnothing) \Rightarrow (ES = C) \qquad (21)$$

DISCUSSION

In this section we will attempt to respond to the question of which mental processes are involved in both the modeling phase of our experiment and the overall evaluation of the ethical story. Grác (2008, 2015) assumes that in modeling ethical stories, semantic operations are involved to create sense-oriented structures in accordance with the sequences determined by the formal signs of the indicators of morality which constitute the cognitive schemas of potential stories (moralities). We can only add that these semantic mental operations must be focused both on securing particular sequences for the appropriate moral valuation required by the cognitive schemas obtained in the experiment, and, at the same time, on maintaining the thematic coherence of the content of the whole ethical story.

We consider here the role in moral reasoning played by the particular indicators of morality (see: Moore, 1980) known from the literature. In the overall evaluation phase of the experiment, moral reasoning required more syntactic and formal logic cognitive operations. Grác distinguishes three processes that enabled the subjects to formulate the regularities in concluding the overall moral evaluation of ethical stories: selection, differentiation and integration.

The selection process deals with highlighting the indicators of morality that do not influence moral evaluation of ethical stories, but only create a situational context in these

stories. These indicators appeared to be: the outcome indicator (O) in the IAO schemas, and the knowledge indicator (K) in the KCR schemas. This means that these two indicators are not considered as premises in completing overall moral evaluation of ethical stories. The result of the differentiation process is in distinguishing among the indicators of morality which play a dominant role in competing the overall moral evaluation of moral stories, and those which are only connected with this evaluation. The dominant positions were taken by: the application indicator (A) in the IAO schemas and the concordance indicator (C) in the KCR schemas.

is this substantiated in research, or is this novel?

The main mental process related to conducting an overall moral evaluation is integration, which allows a generalizing value to be assigned for each group of three indicators in the IAO and KCR schemes. As a result of this process, seven regularities were identified that describe the generalized patterns of indicator sign in the premises and the inferred overall conclusion for the each group of ethical stories. The relationship discovered between each of the particular groups of indicator signs as premises and the overall moral evaluations of the moralities constitutes a psychological connection for moral inference in our experiment on moral evaluations of ethical stories. Formally, this relationship can be expressed in the formal logic syllogism *modus ponendo ponens* as follows:

$$[(p \Rightarrow q) \cap p] \Rightarrow q \qquad (22),$$

where the symbols of inference connection ($p \Rightarrow q$) denote the particular regularities formulated in (17)-(21) as the first premise for conducting the moral evaluation, where p denotes the conditional sentence concerning the particular group of indicators signs of moral valuation and q denotes the result of the overall moral evaluation of ethical story.

The above ~~discussion~~ statement corresponds with A. Biela's (1986) commentaries on R.J. Sternberg's (1984) triarchic theory of human intelligence and his positive response to these

comments (Sternberg, 1986). Starting from the statement that higher levels of moral development are related to higher levels of cognitive development, Biela suggested that it is possible to include moral/axiological level to a theory of human intelligence without any decrease of its empirical testability. The presented experiment on moral reasoning evidently supports this suggestion.

this is interesting. Advice, cute much of your current lit review in the introductory pages, and tell us why this is important, about Sternberg etc here. That may better ground the apparent purpose of this paper, which is interpreting what are effectively structured survey results.

Concluding Remarks

In conclusion, we describe the novelty of the two phase experiment conducted on moral reasoning, which included a moral story modeling phase and an overall moral evaluation phase developed by the same subjects, educational psychology students.

The moral story modeling phase developed by the students gave them the chance to learn what moral reasoning about youth behavior is in an attractive way, and what role the indicators of morality of their experimentally modeled ethical stories, play in overall moral assessment of human performance. The modeling phase of the experiment enabled the subjects to take a large step towards the process of internalization of moral norms regulating human behavior in social situations. In addition, this process was of a multidimensional nature and a structured character, leading to the overall moral evaluation of human action.

In this experiment, students were able to create models of complex matter, using the indicators of moral evaluation of their own youth social performance. Thus, we may say that this experiment took place in closer proximity to real-life thinking processes than any other research method previously used in experimental psychology.

Financial Support / Funding Statement: This research received no specific grant from any funding agency, commercial or not-for-profit sectors.

Conflicts of Interest: None

References

Alfano, M. (2019). Moral reasoning is the process of asking moral questions and answering them. Behavioral and Brain Sciences, 42, E147. doi:10.1017/S0140525X18002534

Biela A. (1986). Toward a synthetic approach to intelligence. Behavioral and Brain Sciences, 9(3),578-579.

Biela A. (2015). Przedmowa do wydania polskiego. In J. Grac, Psychologiczne analizy procesów interioryzacji norm moralnych. Lublin: Towarzystwo Naukowe KUL.

Bucciarelli, M., Khemlani, S., & Johnson-Laird, P. N. (2008). The psychology of moral reasoning. Judgment and Decision Making.

Calmeiro, L., Stoll, S. K., & Davis, P. (2018). Moral Reasoning in Sport: Validation of the Portuguese Version of the RSBH Value-Judgement Inventory in Adolescents. Sport Science Review, 24(5–6), 285–304. https://doi.org/10.1515/ssr-2015-0020

Candee D. (1976). Structure and Choice in Moral Reasoning. Journal of Personality and Social Psychology, Vol. 34, No. 6, 1293-1301.

Chien, C. F., Liao, C. J., Walters, B. G., & Lee, C. Y. (2016). Measuring the Moral Reasoning Competencies of Service-Learning e-Tutors. Educational Technology & Society, 19 (3), 269–281.

Choi, Y. J., Han, H., Dawson, K. J., Thoma, S. J., & Glenn, A. L. (2019). Measuring moral reasoning using moral dilemmas: evaluating reliability, validity, and differential item functioning of the behavioural defining issues test (bDIT). European Journal of Developmental Psychology, 16(5), 622–631. https://doi.org/10.1080/17405629.2019.1614907

Crockett, M. J. (2013). Models of morality. Trends in Cognitive Sciences, 17(8), 363–366.

Cushman, F., Young, L., & Hauser, M. (2006). The role of conscious reasoning and intuition in

moral judgment: Testing three principles of harm. Psychological Science, 17(12), 1082–1089.

Czyżowska D., Niemczyński A. (2006) Universality of Socio-moral Development: a cross-sectional study in Poland, Journal of Moral Education, 25:4, 441-453.

Dewey J. (1910). How We Think. Boston: D. C. Heath & Co., Publishers.

Ellemers, N., van der Toorn, ., Paunov, Y. & van Leeuwen, T. (2019). The Psychology of Morality: A Review and Analysis of Empirical Studies Published from 1940 through 2017, Personality and Social Psychology Review, 23 (4), 332-366.

Fang, Z., Jung, W. H., Korczykowski, M., Luo,L., Kristin Prehn,K., Xu, S., Detre,J., A., Kable, J. W., Robertson, D. C., & Rao, H. (2017). Post-conventional moral reasoning is associated with increased ventral striatal activity at rest and during task. Scientific Reports, 7 (1) DOI: 10.1038/s41598-017-07115-w

Gehrlein, J., Crede, A. K., & Adrian, N. (2020). The impact of markets on moral reasoning: Evidence from an online experiment. Journal of Behavioral and Experimental Economics , 87, 101577. https://doi.org/10.1016/j.socec.2020.101577

Gibbs, J. C. (2006). Should Kohlberg's cognitive developmental approach to morality be replaced with a more pragmatic approach? Comment on Krebs and Denton. Psychological Review, Vol. 113(3), July, 666-671.

Grac J. (2008). Psychologia mravnosti v teoretickych a empirickych analizach. Trnava: Filozoficka fakulta Trnavskej univerzity.

Grac J. (2015). Psychologiczne analizy procesów interioryzacji norm moralnych. Lublin: Towarzystwo Naukowe KUL.

Greene, J. D., Morelli, S. A., Lowenberg, K., Nystrom, L. E., & Cohen, J. D. (2008). Cognitive

load selectively interferes with utilitarian moral judgment. Cognition, 107(3), 1144–1154.

Greene, J. D., Cushman, F. A., Stewart, L. E., Lowenberg, K., Nystrom, L. E., & Cohen, J. D. (2009). Pushing moral buttons: The interaction between personal force and intention inmoral judgment. Cognition, 111(3), 364–371.

Haidt, J. (2007). The new synthesis in moral psychology. Science (New York, N.Y.), 316(5827), 998–1002.

Joyce R. (2006). Is Human Morality Innate? In P. Carruthers, S. Laurence, S. Stich (Eds.), Innate Mind. Volume 2: Culture and Cognitive (257-279). New York: Oxford University Press.

Kamali, F., Yousefy, A., & Yamani, N. (2019). Design and psychometric properties of an instrument to assess metacognition in moral reasoning in medicine. Nursing Open, 6(4), 1331–1345. https://doi.org/10.1002/nop2.331

Kohlberg, L. (1969). Stage and Sequence: The Cognitive Developmental Approach to Socialization. In D. Goslin, (Ed.), Handbook of Socialization Theory and Research (347-480). Chicago, IL: Rand McNally.

Kohlberg, Lawrence (1971). From Is to Ought: How to Commit the Naturalistic Fallacy and Get Away with It in the Study of Moral Development. New York: Academic Press.

Kohlberg, L. (1976). Moral stages and moralization: The cognitive developmental approach. In T. Lickona (Ed.),Moral development and behavior: Theory, research and social issues (31-53). New York, NY: Holt Rinehart and Winston.

Kohlberg, L., Candee, D. (1984). The relationship of moral judgment to moral action. In L. Kohlberg (Ed.), Essays in moral development: Vol. 2. The psychology of moral development (pp. 498 –581). New York: Harper & Row.

Kornas-Biela, D. (2019). Inteligencja moralna jako zadanie wychowawcze dla rodziców. In: L. Bakiera (Ed.). Rodzicielstwo. Ujęcie interdyscyplinarne. Uniwersytet im. Adama Mickiewicza w Poznaniu, Poznań : Wydawnictwo Naukowe UAM.

Kornas-Biela D., M. Schwarz, A. Biela-Wołońciej (2018). Moral Intelligence in Job Analysis. In: European Questionnaire for Job Analysis (EQJA). Theoretical and Methodological Bases (s. 157-171). Series: Polish Studies in Economics. Berlin: Peter Lang GmbH, Internationaler Verlag der Wissenschaften. ISBN:978-3-631-73015-7. DOI: https://doi.org/10.3726/b11542.

Hanna, L. A., Gillen, J., & Hall, M. (2017). An investigation of final year pharmacy students' moral reasoning ability, and their views on professionalism and fitness to practice panel determinations: A questionnaire study. Currents in Pharmacy Teaching and Learning, 9(4), 652–659. https://doi.org/10.1016/j.cptl.2017.03.016

Moll, J., & de Oliveira-Souza, R. (2007). Moral judgments, emotions and the utilitarian brain. Trends in Cognitive Sciences, 11, 319–321.

Moore G. E. (2005). Ethics. Oxford, Oxford University Press.

Nikolaicev, B. O. (1976). Osoznavaemoe i neosoznavaemoe v nravstvennom povedenii licnosti. Moskva: Izdavatelstvo Moskovskogo universiteta.

Paciello M., Fida R., 2, Carlo Tramontano C., Cole E., Cerniglia L. (2013). Moral dilemma in adolescence: The role of values, prosocial moral reasoning and moral disengagement in helping decision making. European Journal of Developmental Psychology, Vol. 10, No. 2, 190-205.

Patil, I., Zucchelli, M. M., Kool, W., Campbell, S., Fornasier, F., Calò, M., … Cushman, F. (2020). Reasoning Supports Utilitarian Resolutions to Moral Dilemmas Across Diverse

Measures. Journal of Personality and Social Psychology, (January). https://doi.org/10.1037/pspp0000281

Piaget, J. (1932). The Moral Judgment of the Child. The Free Press, New York.

Piaget J (1957). The construction of reality in the child. New York: Basic Books.

Piaget J. (1976) Piaget's Theory. In B. Inhelder, H. H. Chipman, C. Zwingmann (Eds.), Piaget and His School. A Reader in Developmental Psychology (11-23). New York, Berlin, Heidelberg: Springer-Verlag New York Inc.

Rest J. R. (1973). The hierarchical nature of stages of moral judgment. Journal of Personality, 41 (1), 86-109.

Rest J. R. (1975). Recent Research on an Objective Test of Moral Judgment: How the Important Issues of a Moral Dilemma Are Defined. In D. J. DePalma, M. Foley (Eds.), Moral Development. Current Theory and Research (75-93). Hillsdale, New Jersey: Lawrence Erlbaum Associates, Publishers.

Rhim, J., Lee, G. bbeum, & Lee, J. H. (2020). Human moral reasoning types in autonomous vehicle moral dilemma: A cross-cultural comparison of Korea and Canada. Computers in Human Behavior, 102(August 2019), 39–56. https://doi.org/10.1016/j.chb.2019.08.010

Roche, C., Thoma S. (2017). Insights From the Defining Issues Test on Moral Reasoning Competencies Development in Community Pharmacists. American Journal of Pharmaceutical Education; 81 (8) Article 5913, 21-32.

Shou, Y., & Song, F. (2017). Decisions in moral dilemmas: The influence of subjective beliefs in outcome probabilities. Judgment and Decision Making.

Sternberg, R.J. (1984).Toward a triarchic theory of human intelligence, Behavioral and Brain Sciences, 7(2), 269-315.

Sternberg, R.J. (1986). Alternatives to the triarchic theory of intelligence, Behavioral and Brain Sciences, 9(3), 581-583.

Sudić, M., Valerjev, P., & Ćirić, J. (2019). Deontic moral reasoning task: Is moral reasoning special? Psihologijske Teme, 28(3), 483–506. https://doi.org/10.31820/pt.28.3.2

Yiym S., Song F. (2017). Decisions in moral dilemmas: The influence of subjective beliefs in outcome probabilities. Judgment and Decision Making, Vol. 12, No. 5, September, 481-490.

von Grundherr, M., Geisler, A., Manuel Stoiber, M. & Schäfer, M. (2017). School Bullying and Moral Reasoning Competence, Social Development 26 (2), 278-294.

Williams R. N., Gantt E. E. (2012) . Felt moral obligation and the moral judgment–moral action gap: toward a phenomenology of moral life. Journal of Moral Education Vol. 41, No. 4, December, 417–435.

Wilmoth G. H., McFarlang S. G. (1977). A Comparison of Four Measures of Moral Reasoning. Journal of Personality Assessment, 41, 4, 396-401.

Figure Click here to access/download;Figure;Figures.docx

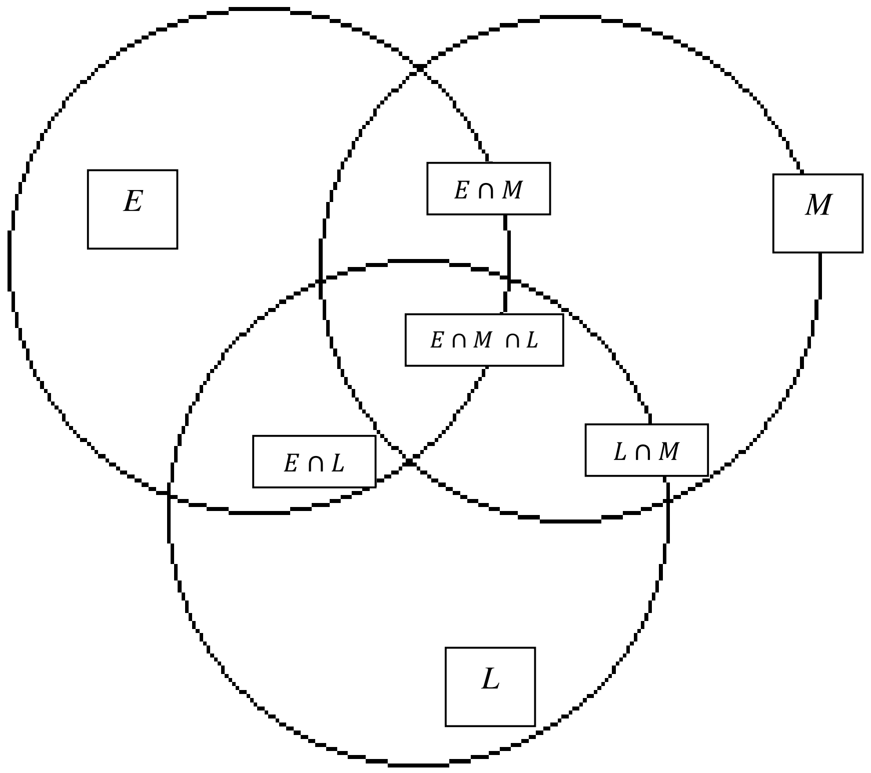

Figure 1. The internormative extensional relations between the executive, moral and legislative norms.

Click here to access/download
Supporting Information
APPENDIX 1.docx
