# Supplementary material for: Can moral reasoning be modeled in an experiment?
Source: PLoS One. 2021 Jun 10;16(6):e0252721. doi: 10.1371/journal.pone.0252721 (PMC8191952; doi:10.1371/journal.pone.0252721)
Supplement: Attachment — Submitted filename: PONE-D-20-37673_R1 WHITE EDITED.pdf [file pone.0252721.s005.pdf]

PLOS ONE

Can moral reasoning be modeled in an experiment?

--Manuscript Draft--

**Manuscript Number:**	PONE-D-20-37673R1
**Article Type:**	Research Article
**Full Title:**	Can moral reasoning be modeled in an experiment?
**Short Title:**	The experiment on moral reasoning
**Corresponding Author:**	Piotr Janusz Mamcarz, Doctor<br>The John Paul II Catholic University of Lublin<br>Lublin, POLAND
**Keywords:**	moral reasoning; decision making; experiment; moral indicators
**Abstract:**	A review of the literature on moral issues indicates that none of the empirical approaches to moral reasoning proposes experimental approach which controls in one research procedure such object-related experimental variables like: motivation, acceptance of moral norms and consequences of human behavior in moral situations. A unique element of proposed experimental method is a multi-stage model determining morality indicators. In the two-phase design experiment, 147 educational psychology students were asked to create model ethical stories and then make an overall assessment of each of these stories.<br>As a result, a base of ethical stories was created with empirical moral indicators (positive, negative, neutral). The patterns in the moral evaluation of ethical stories were determined by identifying three processes (selection, differentiation and integration). The final result is a confirmed design of the experiment and a set of formulas that can be used in education and research on morality reasoning.<br>Key words: cognitive schemas of ethical-dilemma-stories; modeling of ethical stories; moral reasoning experiment; morality indicators; overall moral evaluation of ethical stories; synthetic approach to intelligence.
**Order of Authors:**	Ján Grác
	Adam, Biela
	Piotr Janusz Mamcarz, Doctor
	Dorota Kornas-Biela
**Response to Reviewers:**	ANSWERS FOR THE REVIEWERS<br><br>For Reviewer #1<br><br>Your comments were, indeed, a well-chosen remedy for the first part of the article. I treated them as good indicators for improving the internal consistency of this part of the study in terms of its substantive coherence. Hence, at your suggestion, Part One has generally been reorganized as follows:<br>1.The main goal of the paper is stated at the very beginning, i.e. the presentation of Jan Grác's (2008) experimental approach to the study of moral reasoning with the possibility of controlling some indicators of morality as independent variables. This methodological approach is presented first in the context of the classic approaches to moral reasoning, and then in the latest contemporary research, with particular emphasis on the possibility of experimental exploration of this research field.<br>2.After pointing to some contemporary authors known in the literature on the subject, I turned my attention to Piaget and Kohlberg as the classic authors of research on moral reasoning. At the same time, I indicated the relationship between their concepts and methodologies and Grác's proposed methodology.<br>3.At the same time, a significant number of paragraphs have been completely removed from the text of the article as being inconsistent with the main goal of our analysis.<br>4.I consider the meta-analysis to be an essential part of the introduction. However, I have limited myself (in accordance with your recommendation) to relations with those items which directly concern experimental research on moral reasoning.<br>5.After these preliminary analyses, J. Grác's theoretical and methodological statements

Powered by Editorial Manager® and ProduXion Manager® from Aries Systems Corporation

on moral reasoning are presented in more detail (pp.10-14 of the reedited manuscript).
6.Regarding your comments on Kant's CI, I have decided to refer to his fundamental work in this area for his own definition (E. Kant (1966). Groundwork of the Metaphysics of Morals, in I. Kant, Practical Philosophy, translated by Mary Gregor, Cambridge; Cambridge University Press, Chapter 4, page 421).
7.Regarding the “no systematic exposure of indicators,” the corresponding paragraph in the text has been reformulated.
8.The use of the Venn diagram in the analysis of the range relations between three different names of standards (moral, legal, executive) is justified by the necessity to strictly distinguish them in research practice. In this spirit, an appropriate paragraph explaining this issue was added to the text.

For Reviewer #2

1.As the first remarks of Reviewer # 2 are in harmony with the remarks of Reviewer # 1, we respond that the introductory part has been reworked towards making it more coherent and shorter.
2.Thanks for your attention regarding mixing the use of symbols in formalization formulas. In the end, I decided to standardize the symbolism in accordance with the notation of set theory, as these formalizations basically relate to the range relations between set names designates (e.g. moral, legal, executive norms in a Venn diagram, or in moral evaluation, which is also based on set operations).
3.You are right about the ambiguity of the experimental procedure presented in the first version of our manuscript and of the tested persons. Your objections on this matter are of vital importance. Hence, our work required clarification of such issues as: 1) the studied groups of students; 2) specification of which part of the experiment they participated in; and 3) presentation of the results of research on the strategies used by the respondents to evaluate ethical stories in the second part of the experiment. For this reason, the authors of this study decided to select two other groups of young people applying to study psychology for the second part of the experiment, i.e. for overall evaluation of the ethical stories (modeled in the first part of the experiment) as subjects. They had just completed secondary school and were taking their entrance exams for psychology at the University of Trnava. These subjects are called Subject Group 2.Their number is - 450 (N=450). The relevant fragments entered in the manuscript are marked in red.
4.Your comments related to the Formula (1). I decided to precise the notion of this formula as follows:

$$CN = \{E, M, L, (E \cap M), (L \cap M), (E \cap L) (E \cap M \cap L)\}$$

I am operating with the symbol $\dot{M}$ (and also with M) which denotes the subclass of moral norms that do not belong conjunctively either to executive norms or to legislative ones. This symbol refers to the moral norms which I have called pure or context-free moral norms. I have marked these items in red. The symbol M in our analysis denotes the universal class of moral norms.
5.Thank you very much for the very insightful remarks on formal defects in the records of some symbols (as in Formula 7 and formulations concerning the third regularity of general moral evaluation according to the third rule of the IAO scheme). Appropriate adjustments have been made.

For Reviewer # 3

1.Your first critical remark concerns whether the IAO / KCR schemes used in the experiment are a kind of limiting template imposed on ethical stories. You also gave interesting and inspiring comparisons of our experiments with other paradigms of research on moral reasoning. An example may be the classic "trolley problem" with references to indicators of morality in moral schemes of the IAO / KCR. Thank you for this suggestion, as the classic "trolley problem" could be positioned within the moral framework of our experiment in future research.

It is true that in every experiment the research scheme, often called a paradigm, and in our experiment a cognitive schema (IAO and KCR), limits the freedom of the experimenter and the subjects. This is the downside to all experiments by definition. However, this limitation ensures in our experiment both the possibility for the experimenter to control the independent variables (IAO morality indicators in one

Powered by Editorial Manager® and ProduXion Manager® from Aries Systems Corporation

	scheme and KCR indicators in the other scheme) as well as the precision of this control by significantly reducing the contaminating influence of uncontrolled factors on the dependent variable – which is the general moral evaluation of the ethical story. As you noticed in your reporting part of the review, the design of the experiment provides for precise control within one cognitive schema, combinatorically defined 27 values of three indicators of morality. Due to the bounded rationality of the human mind, more indicators of morality cannot be included in one framework. The selection of only two morality indicators for experimental control gives the possibility of precise control of 9 values of these variables (+, -, O), but the two-indicator moral schemas seemed to have too little context. Therefore, it was decided to choose Aristotle's "golden mean" in our experiment.<br>2.We have simply avoided the claim that our experiment is “natural.” Your arguments convince us.<br>3.What characterizes executive norms? They are unambiguous regulators of the behavior manifested in specific situations, e.g. in contact with nature, with other people, with oneself. They are oriented towards the hic et nunc point of view, which is the optimum performance of certain activities. For example, in a sport, a sports record can be quickly replaced by another sports record (ski jumping hill record marked in green to be visible to the jumpers). Another clear feature of this type of normative regulation is that although they improve human activities and accompany various mental experiences (e.g. attitudes, motives), the content of these experiences themselves is not subject to assessment by these norms. Thus, it can be said that neither the views nor the feelings accompanying the performance of these activities are subject to regulation within the framework of moral norms.<br>4.Your critical remark concerns the use of the term "moral norm" in two different senses: (a) as a virtue (e.g., courage) in the sense of Aristotle's list of virtues; and (b) as a normative rule or moral principle. We would like to clarify that in the first sense we are referring to moral content by basically discussing some items from the subject literature in the introductory part of the article. In its revised version, many of these items have been abandoned and therefore are not included in the text. However, we cannot take advantage of your suggestion to replace the meaning (b) of the term with the meaning (a), because it is fundamental to Grác’s concept and necessary in the conceptual-methodological system of our entire experiment.<br>5.We agree with the comments on G.E. Moor, and therefore we have excluded these text fragments from the revised manuscript as unrelated to the merit of this article.<br>6.In our revised version of the article you can find the appropriate formulation of Categorical Imperative based on Kant’s (E. Kant (1966) Groundwork of the Metaphysics of Morals, in I. Kant, Practical Philosophy, translated by Mary Gregor, Cambridge; Cambridge University Press, Chapter 4, page 421).<br>7.As far as the concepts “mravnost” and “moralnost” are concerned, a special short paragraph has been prepared which synthetically explores the novelty of these concepts.
**Additional Information:**	
**Question**	**Response**
**Financial Disclosure**<br><br>Enter a financial disclosure statement that describes the sources of funding for the work included in this submission. Review the submission guidelines for detailed requirements. View published research articles from *PLOS ONE* for specific examples.<br><br>This statement is required for submission and **will appear in the published article** if the submission is accepted. Please make sure it is accurate.	This research received no specific grant from any funding agency, commercial or not-for-profit sectors.

**Unfunded studies**<br>Enter: *The author(s) received no specific funding for this work.*<br><br>**Funded studies**<br>Enter a statement with the following details:<br>• Initials of the authors who received each award<br>• Grant numbers awarded to each author<br>• The full name of each funder<br>• URL of each funder website<br>• Did the sponsors or funders play any role in the study design, data collection and analysis, decision to publish, or preparation of the manuscript?<br>• **NO** - Include this sentence at the end of your statement: *The funders had no role in study design, data collection and analysis, decision to publish, or preparation of the manuscript.*<br>• **YES** - Specify the role(s) played.<br><br>* typeset	
**Competing Interests**<br><br>Use the instructions below to enter a competing interest statement for this submission. On behalf of all authors, disclose any competing interests that could be perceived to bias this work—acknowledging all financial support and any other relevant financial or non-financial competing interests.<br><br>This statement **will appear in the published article** if the submission is accepted. Please make sure it is accurate. View published research articles from *PLOS ONE* for specific examples.	None

**NO authors have competing interests**<br><br>Enter: *The authors have declared that no competing interests exist.*<br><br>**Authors with competing interests**<br><br>Enter competing interest details beginning with this statement:<br><br>*I have read the journal's policy and the authors of this manuscript have the following competing interests: [insert competing interests here]*<br><br>* typeset	
**Ethics Statement**<br><br>Enter an ethics statement for this submission. This statement is required if the study involved:<br><br>• Human participants<br>• Human specimens or tissue<br>• Vertebrate animals or cephalopods<br>• Vertebrate embryos or tissues<br>• Field research<br><br>Write "N/A" if the submission does not require an ethics statement.<br><br>General guidance is provided below. Consult the submission guidelines for detailed instructions. **Make sure that all information entered here is included in the Methods section of the manuscript.**	The Deparment of Psychology Ethical Board (Trnava University). This reasearch was approved by this committee. The data were analyzed anonymously but the oral consent was obtained before the subject research.

Powered by Editorial Manager® and ProduXion Manager® from Aries Systems Corporation

**Format for specific study types**<br><br>**Human Subject Research (involving human participants and/or tissue)**<br>• Give the name of the institutional review board or ethics committee that approved the study<br>• Include the approval number and/or a statement indicating approval of this research<br>• Indicate the form of consent obtained (written/oral) or the reason that consent was not obtained (e.g. the data were analyzed anonymously)<br><br>**Animal Research (involving vertebrate animals, embryos or tissues)**<br>• Provide the name of the Institutional Animal Care and Use Committee (IACUC) or other relevant ethics board that reviewed the study protocol, and indicate whether they approved this research or granted a formal waiver of ethical approval<br>• Include an approval number if one was obtained<br>• If the study involved *non-human primates*, add *additional details* about animal welfare and steps taken to ameliorate suffering<br>• If anesthesia, euthanasia, or any kind of animal sacrifice is part of the study, include briefly which substances and/or methods were applied<br><br>**Field Research**<br><br>Include the following details if this study involves the collection of plant, animal, or other materials from a natural setting:<br>• Field permit number<br>• Name of the institution or relevant body that granted permission	
**Data Availability**<br><br>Authors are required to make all data underlying the findings described fully available, without restriction, and from the time of publication. PLOS allows rare exceptions to address legal and ethical concerns. See the PLOS Data Policy and FAQ for detailed information.	No - some restrictions will apply

A Data Availability Statement describing where the data can be found is required at submission. Your answers to this question constitute the Data Availability Statement and **will be published in the article**, if accepted.<br><br>**Important:** Stating 'data available on request from the author' is not sufficient. If your data are only available upon request, select 'No' for the first question and explain your exceptional situation in the text box.<br><br>Do the authors confirm that all data underlying the findings described in their manuscript are fully available without restriction?	
**Describe where the data may be found in full sentences. If you are copying our sample text, replace any instances of XXX with the appropriate details.**<br><br>• If the data are **held or will be held in a public repository**, include URLs, accession numbers or DOIs. If this information will only be available after acceptance, indicate this by ticking the box below. For example: *All XXX files are available from the XXX database (accession number(s) XXX, XXX.).*<br>• If the data are all contained **within the manuscript and/or Supporting Information files**, enter the following: *All relevant data are within the manuscript and its Supporting Information files.*<br>• If neither of these applies but you are able to provide **details of access elsewhere**, with or without limitations, please do so. For example:<br><br>*Data cannot be shared publicly because of [XXX]. Data are available from the XXX Institutional Data Access / Ethics Committee (contact via XXX) for researchers who meet the criteria for access to confidential data.*<br><br>*The data underlying the results presented in the study are available from (include the name of the third party*	The data underlying the results presented in the study is available from the Trnava University Psychology Department Archives in paper form (it was not digitalized). Researchers can access the data by contacting PhDr. Marián Špajdel, PhD who has direct access to the data at mspajdel@gmail.com and marian.spajdel@truni.sk.

and contact information or URL).<br>• This text is appropriate if the data are owned by a third party and authors do not have permission to share the data.<br><br>* typeset	
Additional data availability information:	

Manuscript Click here to access/download;Manuscript;Manuscript.doc

Can moral reasoning be modeled in an experiment?

Ján Grác

Department of Psychology

Trnava University

Hornopotočná 23, 918 43 Trnava, Slovakia

e-mail: grac.j.z.2@pobox.sk

Adam Biela

Department of Social Psychology

Maria Sklodowska-Curie University

ul Głęboka 44, 20-612 Lublin, Poland

e-mail: adam@biela.pl

ORCID ID: 0000-0001-5730-7693

Piotr Mamcarz - Corresponding author

Department of Emotion and Motivation Psychology

The John Paul II Catholic University of Lublin

Al. Racławickie 14, 20-950 Lublin, Poland

e-mail: pmamcarz@kul.pl

Phone. +48 509 630 598 Fax +48 81 445 33 40

ORCID ID: 0000-0001-6307-6326

Dorota Kornas-Biela

Department of Psychopedagogy

The John Paul II Catholic University of Lublin

Al. Racławickie 14, 20-950 Lublin, Poland

e-mail: dorota@biela.pl

ORCID ID: 0000-0001-6002-1901

Can moral reasoning be modeled in an experiment?

Abstract

A review of the literature on moral issues indicates that none of the empirical approaches to moral reasoning proposes an experimental approach which controls for such object-related experimental variables as: motivation, acceptance of moral norms and consequences of human behavior in moral situations in a single research procedure.

A unique element of the proposed experimental method is a multi-stage model determining morality indicators. In the two-phase design experiment, 147 educational psychology students were asked to create model ethical stories and then conduct an overall assessment of each of these stories.

As a result, a base of ethical stories was created with empirical moral indicators (positive, negative, neutral). The patterns in the moral evaluation of ethical stories were determined by identifying three processes (selection, differentiation and integration). The final result is a confirmed design of the experiment and a set of formulas that can be used in education and research on morality reasoning.

Key words: moral reasoning; decision making; experiment; moral indicators

INTRODUCTION

In our study, we present an experimental approach to the study of moral reasoning which allows for the control of some indicators of morality as independent variables. A research proposal for this new approach was first presented by the Slovak educational psychologist Jan Grác (2008). This proposal will be presented first in the context of the classic approaches to moral reasoning, and then in the latest contemporary research, with particular emphasis on the opportunities for experimental exploration of this research field.

Empirical studies on moral reasoning are well known in the psychological and education al literature (Rest, 1975; Czyżowska, Niemczyński, 2006; Bucciarelli, Khemlani, & Johnson-Laird, 2008; Alfano, 2019; Ellemers et al., 2019). Most of them are inspired by the two early concepts on structure and choice in moral reasoning offered by Piaget (1932, 1957) and Kohlberg (1969, 1971).

Classic approaches to moral reasoning

Piaget (1932, 1957) began his research on moral development using the clinical-experimental approach. This approach was based on short dialogues with individual small children in the context of a moral issue situation. The dialogue with a child, called an *indagation*, aimed to ascertain his or her individual understanding of moral concepts. Piaget proposed a model of stages of moral reasoning which states as one of its premises that qualitative changes in the structure cause the appearance of a new stage at specific points in time. It could be said that he began a methodological revolution, which shifted psychologists' orientation from analyzing an individual`s behavior or choice to the underlying form and structure of moral reasoning. Piaget's research approach was a kind of

Do you mean to say all of the value or part of the value?

transposition of the maieutic method of Socratic dialogues into the situation of a conversation with a young child on moral issues. He created a non-quantitative but qualitative and creative method of scientific exploration of moral thinking in young children. The creative value of Piaget's clinical experiment was used by Grác (2008, 2015) to create a phase of the experiment called modeling ethical stories by students.

Another approach by Kohlberg (1969, 1971) is based on hypothetically constructed moral dilemmas; it aims to test moral decision-making in youth and adults. Kohlberg traced the development of moral structures through a series of six stages which form an invariant and culturally universal sequence (Gibbs, 2006). Moral structures are measured by requesting individuals to reason about cases in which two or more conventional values are in conflict. The subject reacts to a moral dilemma, indicating what ought to be done and justifying this course of action. The interviewer tries to elicit and probe the subject`s views without interjecting or suggesting any thinking different from the subject`s own spontaneous thinking. The subjects’ behavior is interpreted in terms of competence levels of moral reasoning (Roche and Thoma, 2017).

Recently, new authors in psychological investigations of moral reasoning have frequently employed moral dilemmas (e.g. Shou & Song, 2017; Crockett, 2013; Paciello et al., 2013).

One sentence? Then look back to 1973?

A significant contribution in this regard was made by Rest (1973), who invented a procedure called the Defining Issues Test (DIT) which requires a subject to read a hypothetical moral dilemma and then select those statements that are the most important issues in making a decision about the case from among 12 statements. The research he conducted, however, confirmed that difficulties still exist in the assessment of moral judgment with an objective test (Rest, 1975; Wilmoth and McFarlang, 1977). Thus, the

This paper is about school bullying… is this paper also “analyzing the logical foundations of Kohlberg’s research paradigm”?

question remains as to whether it is sufficient and appropriate to study moral reasoning using only a standardized test with structured responses (Fang, 2017).

Analyzing the logical foundations of Kohlberg's research paradigm (e.g., Candee, 1976; Kohlberg & Candee, 1984; von Grundherr et al., 2017) on hypothetical moral dilemmas, one can draw the conclusion that research on moral evaluation is itself dilemmatic, primarily because of the one-dimensionality in which the dilemmas in moral judgement literature continue to be formulated. In his research approach, Kohlberg could not apply the experimental procedure in a strict manner so that the moral norms present in the tests of his dilemmas would be controlled as independent variables having a hypothetical influence on the moral reasoning contained in the psychometric tests. In his research on moral reasoning, he chose to expose the subjects to standardized tests of moral dilemmas, where the stimuli and test conditions had to remain constant by definition (*ex defintione*). In our experimental study based on Grác’s methodology (2008, 2015), ethical stories will be presented as a multidimensional reality, which allows us to take a new research approach to moral reasoning. In this study, the indicators of morality as an important context of moral evaluation will be treated as independent variables, the influence of which are strictly controlled by the experimenter.

We now proceed to review the results of meta-analysis of the literature on moral reasoning, with particular emphasis on the experimental methodology of these approaches. Ellemers et al. (2019) presented a monumental meta-analysis of empirical studies on the psychology of morality published from 1940 through 2017.

“THE” literature as in ALL of it or… some important pieces?

Meta-analysis outcomes on moral reasoning research approaches

this paper? are you the authors?

Which authors?

This may be true. But, in this paper, can you make their reasoning clear, for the reader?

What are the methodological conclusions from this meta-analysis regarding experimental research on morality? The authors (see: Ellemers et al., 2019) clearly indicate that studies on morality have largely neglected to examine how moral reasoning relates to actions in experimental contexts, such as moral norms and intentions.

Our more recent meta-analysis of moral reasoning studies has allowed us to state that prior research has focused on the following three points: (1) operationalization of moral reasoning abilities by constructing the psychometric tests and measuring their validity as well as reliability (Choi et al., 2019; Patil et al., 2020); (2) inventing experimental approaches on moral reasoning: laboratory experiment (Sudić & Ćirić, 2019), on-line experiment (Adrian & Gehrlein, 2020), and moral reasoning thought experiment (Rhim, Lee G. & Lee, J., 2020); and (3) applied research on moral reasoning in some particular professional domains where morality and ethics is considered to be an important part of professional competencies, such as health care professionals (pharmacists, e.g. Hanna, Gillen & Hall, 2017; Göttgens-Jansen et al., 2019; nurses, e.g., Kamali, Yousefy, & Yamani, 2019), businessmen (Forte, 2006); sportsmen (Calmeiro, Stoll, & Davis, 2015), and computer scientists (Rhim, Lee G. & Lee, J., 2020).

How are these related, such that they belong together as a class?

Only one point of our meta-analysis outcomes, i.e. (2), signals an interest in experimental research on moral reasoning, where all the above-mentioned authors present the results of research that include the paradigm of moral dilemmas. Thus, Sudić & Ćirić (2019) designed the laboratory experiment of 3x2x2 within groups, where the independent variables were: a) content of norm (moral, conventional, or abstract), b) rule type (obligation, or permission), and c) induced dilemma (punishment dilemma, or reward dilemma). Three dependent variables were measured in the experimental task: response time, accuracy and final confidence of performance. One of the main experimental findings

i do not see this in the concluding remarks on page 500 of this paper… do you mean to reference a different passage? Could you please indicate the page? And, though it is true that in the concluding remarks, these authors use the word "easier", but the case that is made is a bit more complex than this…

is that moral rules are easier to process than conventional ones, and conventional rules are easier than abstract ones. However, the authors indicate in their concluding remarks that they are unsure as to what degree conventional and abstract rules contaminated the moral ones. Moreover, this approach is limited because the 'correct' response defined by this experiment is only logical consistency, and it does not take into account real life moral reasoning. In conclusion, this experiment can be treated as a good initial exploration of the problematic multidimensional field of moral reasoning, which is difficult to study using the method of a laboratory experiment.

Authors of a second study (Gehrlein et al., 2020) presented an online experiment investigating the influence of the market environment on moral reasoning. A control group of respondents was asked to make a choice regarding moral dilemmas, while the study participants were exposed to either a market or non-market environment, and after that were asked to solve moral dilemma experimental tasks. While in principle online experiments are not objectionable, our main objection to this research is the fact that it does not consider morality indices as independent variables at all.

The third experimental approach to moral reasoning presented in our meta-analysis is a very intriguing thought experiment by Rhim, Lee & Lee (2020), who studied human moral reasoning types in an autonomous vehicle moral dilemma, in a cross-cultural comparison of Korean and Canadian subjects. These authors enrich our knowledge about the possibilities of simulation studies in the field of artificial moral intelligence regarding descriptive models of drivers' moral reasoning in problematic road traffic situations (e.g. Tunnel Problem (Millar, 2014); MIT Moral Machines (e.g., Awad et al., 2019). The study of moral reasoning in road traffic situations is not exhausted by dilemmas, however. It would be useful to consider the behavior of real drivers to a greater extent, to take into account

moral indicators as factors influencing complex moral thinking processes. It is these more complex processes subject to manipulation in Grác's experiments, which are featured in our study.

Summarizing the above research findings one can state the following outcomes, among others: (a) moral reasoning is a multi-faceted phenomenon where its mutually linked factors constitute the moral existence of the individual human person; (b) there is a need to distinguish and differentiate the following components of moral reasoning processes for both theoretical and practical purposes: cognitive, volitional, emotional, motivational (intentional) and behavioral processes – which the literature refers to as indicators of morality; (c) psychology is still far from determining the external validity of its psychometric and experimental instruments for studying moral reasoning in natural experiment circumstances with a higher ecological validity.

In the context of the above statements, one can pose the question: Is it possible to design an experiment on moral reasoning in circumstances of higher ecological validity, and if so, in what way?

Is this the first time that you have used this term, ecological validity? Why is this important, here, and how did the preceding section tell us about it?

J. Grác's theoretical and methodological statements on moral reasoning

Unfortunately, none of the above approaches to moral reasoning propose any form of research which controls for object-related experimental variables, such as those related to motivation, acceptance of moral norms, and consequences of human behavior in moral situations – in a single research procedure. Grác states that this is possible, and we will demonstrate this in our experiment conditions, where subjects had as a task to define their real-life moral issue as a situation characterized by moral norms involved with the social-moral environment of their professional or educational endeavor. Such arrangements

guarantee appropriate circumstances for significantly higher ecological validity of the experimental procedure.

Moreover, Grác (2008) assumes that moral reasoning is a process where the subject conducts a moral evaluation of a human event based upon some premises. The premises for him or her are the indicators of morality which are analyzed in the particular situations, and then synthetized as an overall moral assessment of their activity (or non-activity) under the given social environment. Thus, a quite intriguing research question is: What kind of strategies do people use in the process of integrating indicators of morality in a particular moral situation into an overall moral evaluation of the human activity that is assessed?

This novel approach to the evaluation of moral reasoning processes is proposed by J. Grác (2008, 2015), who has made a conceptual contribution towards the psychological analysis of the interiorization processes of moral norms. It is worthy of note that Grác in his original Slovak work (2008) distinguishes between the terms: *mravnost'*, and *moralnost'*. In the *Introduction to the Polish edition* of Grác's book, Biela (2015) emphasizes that these two terms only exist in discriminative usage in two contemporary languages, i.e., Czech and Slovak, and the term *mravnost'* is no longer used in Slavic languages other than Czech and Slovak. This certainly means that psychologically, the users of these two languages operate with more developed cognitive and behavioral structures for the moral domain than the users of other European languages. What differentiates the meanings of these two terms?

Grác (2015, p 71) states that the term "mravnost'" denotes a morally good performance as the highest ethical level of evaluation. It can be said that this is so because this term indicates the connection of morally good behavior with the accompanying positive psychological experiences, whereby the moral norm regulating both compounds of the conjunctive relationship: morally good behavior and the accompanying morally good

experience – indicate a deep interiorization of the moral norm constituting the psychological basis of this conjunction. In turn, the term "moralnost’" according to Grác means the externally observed manifestations of morally good behavior without accompanying psychological experiences. Hence, not all performances observed as morally good are simultaneously “mravne” (i.e., moral), but only those where good performance is associated with morally good psychological experiences. Therefore, “mravnost’” is interpreted by Grác as an immanent psychological phenomenon.

What is the psychological novelty of Grác’s conception of morality, and what is its methodological significance for an experimental approach to moral reasoning processes? Its greatest novelty is in extending the conditions of assessment of human performance in the defined situation as morally good or bad. In order to define the criteria enabling such an evaluation, Grác distinguished two parallel analytical dimensions of human performance: (1) moral behavior, i.e. the external revealing of moral decision making (external dimension); (2) the internal experience related to the moral behavior, i.e. intentional focus on the moral norm, motivation (internal dimension). In this way, the psychological understanding of moral norms as factors regulating human performance is enhanced by experiential and behavioral structures, creating a basis for systematizing norms as moral, executive, and legal. Executive and legal norms regulate only externally revealed human performance. The characteristic of moral norms is that they regulate both human behavior associated with internal experiences focused on a particular moral norm as well as these internal experiences themselves (i.e. internal experience not tied with revealed behavior (Grác, 2008).

In this context, we can evaluate an individuals’ ethical conduct in a specific situation as being a morally good action or a morally bad one. However, *morally good* vs. *morally bad* designates human behavior in a concrete situation, independent of whether the specific

moral norm, e.g., hard work, courage, honesty, respect, trustworthiness, dedication, loyalty, has been actualized or not. Grác (2008) analysis posits that a person who accepts a particular moral norm but does not meet it could not be evaluated as performing a morally good action. And, similarly, a person could not be evaluated as having performed a morally good action if that person behaved in a manner consistent with the specific moral norm yet he or she did not accept this norm. Only the conjunction of the positive internal experience of the moral norm with the positive fulfillment of this norm in the situation provides the necessary and sufficient conditions for the positive ethical evaluation of a person acting in a given situation.

The mental processes involved in ethical evaluation of moral performance in everyday life situations and, particularly, in extreme situations are not passive ones on the part of the human subject; they involve active interaction with the social and natural environment.

These processes are truly creative in their nature, as they transform the human life environment into a valuable system of values, called an axiological system. These processes do not present a simple or easy reality for psychological research, or for the experimental approach in particular (Cushman et al., 2006).

Grác (2015) states that moral reasoning implies two sets of mental processes related to moral norms. The first set is related to the mental interiorization of moral norms, and the second set deals with the regulative function of moral norms in an individual's personal life, social interactions, and the natural environment. These mental processes create real-life moral situations, where moral norms interact with executive and legislative norms. Grác (2008, 2015) presents the internormative extensional relations between executive, moral, and legislative norms using Venn diagrams (Venn, 1884), what can be seen in Figure 1. As a

Is there something more contemporary applying Venn's diagrams to such or similar relations?

justification of the purposefulness of using Venn diagrams in our analysis we can state that there is the need to prevent inaccuracies resulting from the extensional crossover of the norms under consideration. Such inaccuracies are indicated by Sudić & Ćirić, (2019) who ~~pointed out that they~~ are unsure to what degree conventional and abstract rules contaminated moral rules in their experiment.

This is not clear.

Space for Figure 1

Figure 1. The internormative extensional relations between executive, moral and legislative norms.

MATERIALS AND METHODS

What is this logical point of view"?

The Venn diagram illustrated in Figure 1 demonstrates that if the logical point of view is taken into consideration, we can see the relations between the three universal classes of norms: executive (E), moral (M), and legislative (L); thus, we can dislocate the extensional connections into the seven separate subclasses of norms. Both the executive and legal norms are described in Grác's (2015) classification as beyond moral ones.

What characterizes executive norms? According to Grác (2008) they are unambiguous regulators of the behavior manifested in specific situations, e.g., in contact with nature, with other people, with oneself. They are oriented towards the *hic et nunc* point of view, which is the optimum performance of certain activities. For example, in a sport, a sports record can be quickly replaced by another sports record (ski jumping hill record is marked in green to be visible to the jumpers). Another clear feature of this type of normative regulations is that although they improve human activities and accompany

various mental experiences (e.g., attitudes, motives), the content of these experiences themselves is not subject to assessment by these norms. Thus, it can be said that neither the views nor the feelings accompanying the performance of these activities are subject to regulation within the framework of moral norms.

What do legal norms refer to? Grác (2008) indicates that the subject of their regulation is also the psychological dimension of the behavior manifested, which is characterized by strictly defined effects. The specificity of these terms is that from the point of view of an injunction and prohibition, they strictly and unequivocally demarcate not only the lower limit of the effect (which is still allowed), but also the upper limit of this manifestation (which is already prohibited). The dimension of mental experiences accompanying the behavior, on the basis of a subjective cause, makes it possible to explain the objective effect, which facilitates the legal classification of the committed act as a crime or crime, which allows for the tightening or leniency of the sanction provided for in criminal law.

If we denote the general class of these norm as CN, and the symbol ∩ denotes logical conjunction, the mutual relations presented in Figure 1 can be expressed in Formula (1), respectively:

$$CN = \{E, M, L, (E \cap M), (L \cap M), (E \cap L) (E \cap M \cap L)\} \qquad (1)$$

As moral norms will be the main point of reference in our experimental approach, we will now pay more attention to the subclasses from Formula (1) which contain only such norms. We will define these kinds of moral norms as pure or context-free. The first such subclass, symbolized as $\dot{M}$, denotes the moral norms which belong only to the universe of moral norms but which do not belong to executive or legislative norms. As such, and where *x*

symbolizes any moral norm, and *ε* is a predicator denoting *belonging to*, this subclass may be strictly defined as in Formula (2):

$$\dot{M} = \{x : x \in M \cap \{x \notin E \cap x \notin L\}\} \quad (2)$$

The second two subclasses are the conjunctions of moral norms with legislative norms: M∩L and moral norms with executive norms: E∩M. The appropriate formal explications can be viewed in Formulas (3) and (4), respectively:

$$M \cap L = \{x : x \in M \cap L\} \quad (3)$$

$$M \cap E = \{x : x \in M \cap E\} \quad (4)$$

There is also a subclass of moral norms in Formula (1) which is a conjunction of the three norms, executive, moral, and legislative: E∩M∩L, which is expressed in Formula (5) as:

$$E \cap M \cap L = \{x : x \in E \cap M \cap L\} \quad (5)$$

The moral norms presented above will be employed in our experiment in the context of moral situation referred to as *ethical stories* or briefly, as *moralities.* This method has already been employed in psychological research on moral issues, e.g., by Piaget (1976) and Kohlberg (1976) to diagnose the moral development of children and youth.

It is worth reviewing the strong and weak aspects of using *ethical stories* in psychological research. We propose conducting a short SWOT analysis of *moralities* in empirical research to confront this method with personal experience or observations of real-life moral situations. The results of this analysis are collected in Table 1.

Table 1. The outcome of SWOT comparative analysis outcomes of direct observation of real-life moral situation and ethical stories (moralities).

Methods / Evaluation	Personal experience or direct observation of a moral situation	Ethical stories or moralities
Strong sides of the method	1. Acknowledges the rich context of a moral situation.<br>2. Enables employing an idiographic analysis of ethical evaluations of human behavior.	1. Enables grasping *hic et nunc* the ethical essence of the moral situation.<br>2. Enables employing nomothetic oriented research in ethical evaluation of human behavior.
Weak sides of the method	In many moral situations the moral motives and intentions are hidden to the external observer.	Abstracts from the entire context of the moral situation.

From the outcomes of SWOT analysis, we learn that ethical stories are more easily employed in nomothetic empirical analysis on moral situations. It must be stressed clearly, however, that the subject of our research will be the cognitive aspect of ethical evaluation related to the concept of *morally good performance in moralities* analysis. Cognitive schemas of ethical stories offer the keys to understanding the concept of internalized behavior.

How do you mean “cognitive” here?

Cognitive schemas of ethical stories

Let us review the main concepts related to ethical stories. Indicators of moral context relate to analysis of cognitive schemas of ethical stories. One indicator is a *moral requirement* or *demand* to do something in a defined situation, which is usually expressed by employing the functor of assertive judgement *should* that was systematically elaborated by

Immanuel Kant as one of his three formulations of a *categorical imperative (kategorischer Imperativ),* which is expressed as: "Act only in accordance with that maxim through which you can at the same time will that it become a universal law" (Kant, 1966, 4:421).[1] Without this indicator, none of the moral norms defined in Formulas (1)-(5) could exist at all. If we state that the symbols *CI* denote Categorical Imperative, we can conclude that the norm regulating one's behavior is moral. This moral condition can be expressed as an alternative connection of the particular subsets of moral norms defined in the appropriate Formulas (2)-(5):

Why is this expression important, here?

$$x \in CI \Rightarrow \{[(x \in M) \cap (x \notin E) \cap (x \notin L)] \cup [(x \in M \cap L)] \cup [(x \in M \cap E)] \cup [(x \in E \cap M \cap L)]\} \quad (6)$$

Another indicator of morality is here the object of moral regulation, which is understood as a content or domain regulated by the particular moral norms indicated in (6). Morally good performance assumes a specific object within which a particular moral norm has been actualized. Good examples could be the ethical codes of various professions. It is precisely stated in such codes that particular content-oriented professional activities are extracted from the remaining professional activities to be regulated by moral norms.

Also belonging to the indicators of morality are what causes a moral norm to become the given norm of the content of a the concrete ethical story, that is, what specifically distinguishes the given norm (specified in Formula (6)) from any other outside normative moral activity of a human being (as regulated by legislative – L, or executive – E norm, as seen in Figure 1 and in Formula (1)).

Some of the?

After reviewing the literature on indicators of morality (Moore, 2005; Nikolaičeva, 1976; Piaget, 1976; Kohlberg, 1976), Grác (2008, 2015) decided to apply the following six

[1] According with the Kantian works, the above quoted citation is: (G 4:421), i.e. E. Kant (1966). Groundwork of the Metaphysics of Morals, in I. Kant, Practical Philosophy, translated by Mary Gregor, Cambridge; Cambridge University Press, Chapter 4, page 421.

indicators for the experimental design: intention – I; concordance – C; application – A; outcome – E; knowledge – K; and realization – R. Realizing that moral reasoning related to ethical evaluation of human behavior in real-life situations is definitely a complicated mental process, we have decided to restructure the above indicators into complementary experimental units, in the two cognitive schemes expressed formally as:

$$IAO = I \cap A \cap O \quad (7)$$

How is this related to Kant and to the preceding Venn diagrams that were formalized in logical notation…?

$$KCR = K \cap C \cap R \quad (8)$$

The issue is: what values may the indicators in the Formulas (7) and (8) take? It is not a problem to apply the mathematical symbols (+) and (-) to denote, by analogy, an evaluation of human behavior in the ethical story as morally good or bad, respectively. However, these two bipolar values are not sufficient for moral evaluation of ethical stories, because in these stories we can meet with human behavior which, for various reasons, is beyond moral evaluation. That is why we have introduced a third neutral value that may be assigned to each indicator employed in our experiment. The symbol Ø taken from Boolean algebra is applied here. This value will denote that in terms of the specific indicator, the analyzed human performance is outside or beyond the specific aspect of moral evaluation. Moreover, it has to be emphasized here that in the cognitive schemas constructed in this experiment, the denotation of each indicator of morality is precisely defined not only by marking it as (+), (-) or Ø, but also by giving a particular interpretation to it in respect to the specific moral dimensions in these schemas. These symbols will be called antecedent marks in our analysis. We term these specific interpretations as *definiens*, which will be precisely defined for the particular schemas (see Table 2 and Table 3). Next, we present the cognitive schemas of IAO and KCR as the matrices for this experiment on moral reasoning.

IAO cognitive schemas

Indicators for the moral assessment and their definiens, i.e., criteria for their evaluation are defined in Table 2.

Table 2. The indicators of morality aspects and their definiens, i.e., criteria for their ethical assessment in the IAO schemas.

Indicators of morality aspects	Criteria for their ethical assessment		
**I**NTENTION– the behavior intention, incentive and the moral norm are:	(+) in accord	(-) in discord	(∅) neither
**A**PPLICATION – the form applied in this particular case in relation to the moral norm:	(+) corresponds	(-) does not correspond	(∅) neither
**O**UTCOME **–** consequence accompanying, possible outcome is:	(+) positive	(-) negative	(∅) neither

These indicators are specific conditions under which any moral norm works in a certain life situation. The criteria are definientia (sing. *definiens*) of the moral norm effect, expressing the fact that each of the perpendicularly identical symbols (+ - Ø) has a different notional content – depending on its combination with the indicators.

The first indicator of the IAO schema is Intention – I. It is understood as a motive or intent of the individual’s ethical behavior. As an individual cannot be forced to be moral, the axiological principle in ethics assumes his or her deliberation and free decision-making in the moral norm interactions. For this reason, this indicator is regarded as exceptionally

important. But the indicator of the person's motive or intention cannot be absolutized since the individual's conscious relationship to a moral norm is an inevitable but not sufficient precondition for an ethics-based assessment of the person's behavior. How the concrete behavior will be evaluated from the perspective of intention and motivation depends on the criterion definientia belonging to this indicator. If, for example, the motive or intention of the behavioral event is in accord with the moral norm, then the result of the partial evaluation of behavior is positive (+). If it is in discord, the result is negative (-). The symbol Ø (*nullus*) signals that neither of the previous assessments are valid. Usually, this refers to activities which, due to their content, are regarded as beyond moral, but also to those where the motive of behavior is regulated by a moral norm, but the person is not aware of it.

Application – A, the second indicator of the IAR scheme, answers the question of whether the intention which is moral is or is not obtained by moral means. If the means of the intent's actualization corresponds with the moral norm, the result is positive (+). If the person tried to achieve the intended good by unethical means, their behavior is evaluated negatively (-). This means that the application indicator always assumes re-activation of the original intent and thus there is also the possibility of a greater or smaller change in the application process; consequently, this resultant motive may, but also may not, be in accordance with the original motive. The fact that every application implies not only motivation but also its actualization gives this indicator, according to the IAO scheme, a crucial position in the evaluation of the individual's ethical behavior.

The third indicator of the IAO scheme is Outcome – O, as a possible or an accompanying outcome. The indicator in question is related to the assessed behavioral event. The concept of relation itself can be understood as causal or non-causal. It could be causal in the case where the intention indicator was the cause of the actualized intention

and the consequence indicator was the impact of it. Since the function of the actualized intention was already adopted in the IAO scheme by the intentional indicator (as mentioned above), it is not possible – in the framework of the same scheme – to assign it simultaneously to the third indicator. Therefore, the Outcome indicator can be understood in the given scheme exclusively as non-causal, i.e. as a consequential circumstance which is related to cause and effect, but which cannot be identified with it. From the above-mentioned analysis, it follows that the assessment of the person's behavior by means of three signs (+ - Ø) applies only to the evaluation of the accompanying consequence *per se* and does not influence the overall evaluation of the individual's behavior in the given moral situation.

For the above reasons, the ethical evaluation processes in a moral situation within the cognitive scheme of IAO could be formulated as follows (9):

$$\prod_{I,A,O} \sum_{\{x:x\in\{(+),(-),\emptyset\}\}} = \{[I=(+) \cup I=(-) \cup I=\emptyset] \cap [A=(+) \cup A(-) \cup A=\emptyset)] \cap [O=(+) \cup O=(-) \cup O=\emptyset]\}$$

KCR cognitive schemas

Indicators of moral assessment related to the cognitive schemas KCR and the criteria for their evaluation are presented (so called *definientia*) in Table 3.

Table 3. The indicators of morality aspects and their definiens, i.e. criteria for their ethical assessment in KCR schemas.

Indicators of morality aspects	Criteria for their ethical assessment		
**K**nowledge – as a cognitive component to the	(+)	(-)	(∅)

specific moral norm	exists	doesn't exist	neither
**C**oncordance – acceptance of the particular moral norm	(+) is accepted	(-) is not accepted	(∅) neither
**R**ealizing **–** the moral norm has actually been implemented	(+) fulfilled	(-) not fulfilled	(∅) neither

The first indicator of morality in the KCR schema is Knowledge about moral norms, which regulates human behavior in a given situation. This is really moral knowledge about the specific norm, which can exist as a socially accepted norm within public opinion, regulating interpersonal relations. However, the acting person may or may not have knowledge about this opinion. Thus, the cognition of the moral norm can be very differentiated. The norm can exist as a socially accepted ethical obligation which a concrete individual might not know about. There could also be cases where an individual has a positive attitude toward the defined requirement and accepts it as his or her own moral norm, independently of whether such norm objectively exists as a social standard or not. The symbol Ø can be used to value this indicator, if is really unclear as to whether to assign a value (+) or (-). Or if the social norm is wrong by reader standards?

The second indicator in KCR is Concordance (C) of the person interacting in a given moral situation. Concordance means the actor's personal attitude to the particular moral norm regulating human performance in this situation. According to Grác (2008, 2015), using the signs: (+) or (-) in valuing the indicator C is relatively univocal, but some problems concerning understanding may require the sign Ø. This sign should be employed if the evaluating person has neither a positive nor negative attitude towards the given moral norm

or when this person does not have his or her own understanding of the moral requirements related to the norm in question.

The last indicator Realizing – R refers to whether and how the requirements of the moral norm regulating human behavior are fulfilled. A positive (+) value of this indicator signifies that implementation of the moral norm is fulfilled. This indicator receives a negative value if implementation of the moral norm is not fulfilled in a situation where it is supposed to be. Ethical evaluation faces greater difficulty when considering whether to assign the value Ø. This case is particularly difficult to assign due to the fact that there is no analogy between a neutral attitude towards a moral norm (as in the case of indicator C above) and the neutrality of acting in a concrete situation, when we are dealing with human performance.

In concluding this stage of our analysis, we can state that ethical evaluation processes in a moral-content-situation within the cognitive scheme of KCR can be formulated as follows (10):

$$\prod_{K,R,C} \sum_{\{x:x\in\{(+),(-),\emptyset\}\}} = \{[K=(+) \cup K=(-) \cup K=\emptyset] \cap [R=(+) \cup R(-) \cup R=\emptyset)] \cap [C=(+) \cup C=(-) \cup C=\emptyset]\}$$

What is the significance of this formula for the rest of the paper? How does this formula inform the rest of the work? Can you transcribe this formula in common English, and specify what the reader is supposed to find valuable in this expression? What should a solution to this formula look like? Can you provide an example of an intuitive application?

Control of independent variables in evaluating cognitive schemas of ethical story experiments

The main methodological requirement for any experimental condition is to control the independent variables which make an impact on the dependent variable. In our experiment on moral thinking, the main dependent variable to be explained is the ethical evaluation of human behavior in terms of moral values. It is assumed that moral reasoning is a structured mental process like any other human thinking structured towards problem

What is the relationship between Kant, conscience and moral norms? Does Dewey suggest that conscience is "based on" moral norms" What is the relationship between conscience and norms for Dewey? How is this introduction of Dewey supposed to help to clarify here?

solving. Since John Dewey's *How we think* (Dewey, 1910), psychologists working on problem solving, have distinguished the phases of thinking which lead human thinking to reach the goal of solving a problem. Similar thinking takes place in a real-life moral situation where a human being considers whether and which action should be taken in order to behave in a morally acceptable way, in accordance with his or her conscience – based on a moral norm.

Our experiment will be based on the evaluation of ethical stories (moralities), using the defined structured indicators of morality, called cognitive schemas of the ethical-dilemma-stories, described above as IAO and KCR.

Why is this here? Maybe this belongs in discusion…

The first novelty of this experimental approach is the two-phase design: (1) modeling of ethic-dilemma stories based on logically structured cognitive schemas, and (2) overall ethical evaluation of the modeled morality schemas.

Assessing the effect of any moral norm in conditions of an experiment situation, given by the schemas IAO and KCR, is one methodological challenge; another is grasping these conditions in a person's actual behavior. This can be done by means of modeling written stories which present real-life moral situations. These stories enable the observer to examine intrinsically experienced behavior, e.g., their motivation or intention, which is usually hidden from a second party's retrospection.

We use the term *modeling* for the creation of moral dilemma stories. This involves the assignment of arbitrary content of actually displayed behavior to the respective value indicators of the schema, so that this allocation may accurately correspond to the criterion definientia of the indicators (I,A,O,K,C,R) of morality. Educational psychology students were the subjects of this experiment. They were asked to create model ethical stories concerning moral situations of young students of their age, according to carefully-prepared instructions informing them how to do this task.

Control of independent variables in the experiment

Three question arose in the definition of *modeling*. The first of these is formulated as follows: how many *independent variables*, that is, different configurations of signs, are offered for story modeling by the schemas IAO and KCR, respectively?

In order to answer this question for IAO schemas we have to strictly consider the three indicators of morality where each of them, formally speaking, may take three possible values: (+), (-), Ø. Thus, to calculate all configurations of each indicator combinatorically, where generally its number is *n,* where the number of values that each of the indicators can take may be generally symbolized by *r*, we could apply the formula for variations with repetition n^r. This means that variations with *n* elements of the rth class are all possible configurations of *n* indicators of morality and the *r* values which each indicator can take. In our case, where the number of indicators *n*=3 in the IAO schemas, and the number of values which each of them can take is r=3, the number of all configurations is simply attained using Formula (11) as follows:

$$\{N(IAO){:}\, N(IM) = 3 \;\cap N(VIM) = 3\} \Rightarrow \{N(IAO) = 3^3 = 27\} \qquad (11)$$

where:

N(IAO) – the total number of independent variable configurations related to the indicators of morality and their values in the IAO schemas;

N(IM) – the number of indicators of morality in the IAO schemas;

N(VIM) – the number of values that can be taken by each of the indicators of morality in the IAO schemas.

And, similarly, for the KCR schemas, where the number of indicators is also *n*=3, and the number of values which each of them can take is *r*=3 as well, we can reach the number

of all configurations simply by using Formula (12) being a replication of Formula (11) as follows:

$$\{N(KCR): N(IM) = 3 \cap N(VIM) = 3\} \Rightarrow \{N(KCR) = 3^3 = 27\} \quad (12)$$

where:

N(KCR) – the total number of independent variable configurations related with the indicators of morality and their values in the KCR schemas;

N(IM) and N(VIM) – are as in Formula (11).

The elementary experimental fields of moral evaluation: ethical stories

The second question is whether the same story content, which is modeled according to certain specific criteria of evaluation, can be *modified* in accord with further randomly applied evaluation criteria of the IAO or KCR schemas. In an effort to answer this question, we can alternately replace each indicator of morality by one of the criterion values: (+), (-), Ø. If we take as an example the description of three 18-years-old youths who solved the problem of moving to a new place to live in different ways, we obtain the IAO and KCR schema matrices with nine boxes as a part of 27 total number of combinatorial boxes, accounted separately for the IAO and KCR cognitive schemas, as presented in Formulas (11) and (12), respectively. For the schema matrixed in our experiment the total number of rows *i*=3, and the number of columns is *c*=3. If any line is denoted as *i* and any column as *j*, then any field in these matrices can be expressed as an experimental field EF_{ij} of partial moral evaluation of human performance in ethical story. An example of such EF_{ij} are shown in Table 4 for the IAO schemas.

Table 4. Matrix of IAO schemas illustrating the elementary experimental fields of moral evaluation in ethical stories.

columns / rows	c=1	c=2	c=3
i=1	**I (+)** John found a new flat to create better conditions for his siblings to study.	**A (-)** He did it regardless of the disapproval of his mother, who felt almost mortally hurt by his decision.	**O (∅)** After having moved to his new flat John found out that the signal for his cell phone was the same quality as in the previous location.
i=2	**I (-)** Paul found a new flat to have easier access to drug dealers.	**A (∅)** While moving to the new flat, he decided to travel by train instead by bus.	**O (+)** At the new location he met a neighbor who began to exert a positive influence on him.
i=3	**I (∅)** Peter found a new flat because he wanted to live alone.	**A (+)** By mutual consent with his parents he left their home.	**O (-)** While moving to the new flat he slipped and broke his leg.

As in our case, the IAO scheme is a square matrix, the values in the first line can be written symbolically: $i_1 c_1 = I (+)$, $i_1 c_2 = A (-)$, $i_1 c_3 = O (\varnothing)$ and at the same time they can be checked by reading them verbally in the first row of the chart. The square matrix enables us to read the same story plot variably, i.e., according to an arbitrary configuration of boxes in the lines, without losing the inner logic (meaning). For example, if we select only positive criteria, then the story plot can be expressed in the following notation: $i_1 c_1 = I (+)$, $i_3 c_2 = A (+)$, $i_2 c_3 = O (+)$. When we read it in the chart in this transcript, we find out that the story plot did not lose its inner coherence. It is possible to symbolically record only the minus or only the null configuration in the same way. However, any random two-element combinations are also possible, e.g., combination of two negative and one positive value. In that case, the notation is as follows: $i_2 c_1 = I (-)$, $i_1 c_2 = A (-)$, $i_2 c_3 = O (+)$.

Based on the above, the IAO schemas make it possible not only to create stories differing in their content, but they also offer the opportunity to modify the already-modeled stories.

In a similar way, we will now give an example of experimental fields EF_{ij} of the schemas KCR shown in the square matrix presented in Table 5.

Table 5. Matrix of the KCR schemas illustrating the elementary experimental fields (EF_{ij}) of moral evaluation in ethical stories.

columns / lines	*c*=1	*c*=2	*c*=3
	**K (+)**<br>Igor knew that cruelty to	**C (-)**<br>He was not opposed to ill-	**R (∅)**<br>However, he has never

i=1	animals is immoral.	treatment of animals.	even been in a situation where he watched cruelty inflicted on animals by others.
i=2	**K (-)** Andrew did not know that cruelty to animals is immoral.	**C (∅)** In view of the abuse of animals, he was unclear and of a contradictory position.	**R (+)** Yesterday, when his colleagues mistreated a dog, he protected it.
i=3	**K (∅)** Emil was not sure if cruelty to animals is moral or immoral.	**C (+)** But personally he did not agree with the abuse of animals.	**R (-)** However, yesterday, when colleagues mistreated a dog, he was also cruel to it.

From Table 5 we can see that KCR schemas, similar to IAO schemas, also have a variable structure of stimuli constituted by the content of combinatorial sequences of elementary experimental fields (EF_{ij}) at a level equal to IAO. These experimental fields are indicators of morality of each individual ethical story presented in both the IAO and KCR schemas. The experimental control of independent variables in this experiment deals with combinatorial control of the particular scheme in terms of the formal values (+), (-) and ∅ in IAO and KCR, respectively. Moreover, in order to avoid contradictions between the concrete schemas, on the one hand, and to maintain individual and personal coherence of the ethical

story content, on the other, the subjects were the heroes of each of the 27 IAO schemas and 27 KCR schemas, where each schema actor was assigned a different name in the modeled ethical stories.

Experimental design

In our experiment the stimuli are the individual schemas, which consist of three indicators of morality ordered in the two following sequences: IAO and KCR, where each of the indicators takes only one of three values in its matrix of sequences (see Table 4 and Table 5). The valuated indicators of morality constitute a concrete cognitive schema as a complex sequential stimulus which consists of three particular experimental fields as the objects of ethical individual story modeling in the first phase of the experiment.

Thus, each individual IAO schema can be defined as a well-ordered set of three:

$$Si(\mathrm{IAO}) \stackrel{\mathrm{def}}{=} \langle I, A, O \rangle \qquad (13),$$

where: S_i(IAO) - any of the *n* (*n*=27 in our experiment) IAO schemas; I={(+),(-),∅}**;** A={(+),(-),∅}; O={(+),(-),∅} **–** denote the sets of values which can take particular indicators of morality in the IAO schemas. This indicates that the order of the indicators constituting the IAO schema should not be changed.

In a similar way, each individual KCR schema can be defined as a well-ordered set of three:

$$Si(KCR) \stackrel{\mathrm{def}}{=} \langle K, C, R \rangle \qquad (14),$$

where: S_i(KCR) - any of n (n=27 in our experiment) KCR schemas; K{(+),(-),∅}; C={(+),(-),∅}; R={(+),(-),∅} – denote the sets of values which can take the particular indicators of morality of the KCR schemas.

The methodological analysis conducted allows us to formulate an experimental design to present cognitive schemas for ethical story modeling by educational psychology students. These ethical stories involve moral issues from the students' own community. This design is included in Table 6 as a matrix of formal schemas numbered from 1 to 54 and presented in parallel columns in such a way that numbers for schemas IAO and KCR are located on either side of the formal valuation structures of the indicators of morality.

Table 6. The experimental design: the structure of valuation of indicators in particular IAO and KCR cognitive schemas for experimental modeling of ethical stories by educational psychology students.

IAO schemas	Modeling formal patterns	KCR schemas	IAO schemas	Modeling formal patterns	KCR schemas	IAO schemas	Modeling formal patterns	KCR schemas
1	(+ + +)	28	10	(∅ - -)	37	19	(- ∅ +)	46
2	(+ - +)	29	11	(- - ∅)	38	20	(+ - ∅)	47
3	(- + +)	30	12	(∅∅∅)	39	21	(+ ∅ -)	48
4	(+ + -)	31	13	(- - +)	40	22	(∅ - ∅)	49
5	(+ ∅ +)	32	14	(- + -)	41	23	(∅∅ -)	50
6	(∅ + +)	33	15	(+ - -)	42	24	(- ∅∅)	51
7	(+ + ∅)	34	16	(∅ + -)	43	25	(∅∅ +)	52
8	(- - -)	35	17	(+ - ∅)	44	26	(∅ + ∅)	53
9	(- ∅ -)	36	18	(∅ - +)	45	27	(+ ∅∅)	54

The order number of formal valuations of the IAO schema is always on the left side of the appropriate column for modeling formal patterns in Table 6 and the order number for the KCR schema is on the right side of this column, respectively. The signs of moral evaluation should be read from left to right both for IAO and KCR schemas.

From Table 6 we learn that both cognitive schema enable the planned experiment on moral thinking to control for variability of stimuli structure via evaluated indicators of morality of human performance modeled in experimental conditions during the students' classes.

Subjects, experiment – (a) modeling instructions and procedure, and (b) overall moral evaluation of ethical stories

Subject Group 1

Subject Group 1 were 134 third-year educational psychology students of a modal age of 20 years, who participated in both parts of our experiment, i.e., in the modeling of ethical stories, and in the overall evaluating of these stories modeled by themselves. Their participation in this experiment came as part of a regular course on educational psychology at the Faculty of Philosophy of Trnava University in Trnava (Slovakia) over the years 1998-2000. 80% of the subjects were female students and 20% were males. After participating in theoretical lectures on cognitive schemas of ethical stories and evaluation of related indicators of morality, the students of the seminar on educational psychology were presented with the following task in accordance with these short instructions:

Experiment instructions and procedure for Subject Group 1

As you have learned today from the lecture on education psychology, each schema of an ethical story has its own order number in our experimental matrix and a concrete valuation of its three indicators of morality. Now you will receive data about two IAO schemas and two KCR schemas. Your task will be to creatively model the thinking and performance of a 17-year-old individual, different in each of the projected stories, in accordance with the given values of the individual indicators of morality according to the patterns of potential schemas, the order numbers of which will be generated by random number generator.

After this instruction each individual subject was given the *Ethical Story Modeling Experiment Report Form* (see the form with this protocol in the Appendix) containing four order numbers associated with indicator values for potential ethical stories generated from Table 6 by a random number generator. The subject was instructed about where and what the modeling schema of the concrete story should be in accordance to the experimental design, and that the hero of each story should be an individual person known by his or her name.

Once the modeling phase of the experiment was completed, in the next phase the experimenter asked the same subject to conduct an overall evaluation of each of the constructed ethical stories in his or her protocol as a whole by assigning a global symbol in square brackets [ ] to denote their overall assessment of moral values of human performance as: morally good [+], morally bad [-] or morally neutral [∅] which are now called the concluding symbols.

The experimental modeling procedure and overall moral evaluation of ethical stories took place in a seminar room where the subjects could model the educational stories in

quiet and comfortable conditions. They conducted their experimental modeling and overall moral evaluation individually, sitting separately at seminar tables in small groups. The average time for modeling was about 45 minutes. After completing the story modeling and conducting the overall evaluation of each of the constructed ethical stories, the students gave the completed *Protocol for Experimental Modeling of Ethical Stories* and their completed *Moral Evaluation* back to the experimenter (see: APPENDIX 1).

Subject Group 2

Taking into account that the studied group of students (Subject Group 1) participated in the entire experiment, i.e. in modeling and then in evaluating the entire ethical story, we can state that they had a fairly high level of ethical knowledge, particularly on overall evaluation of ethical stories, gained mainly from lectures on the psychology of morality. This, however, completely rules out the possibility of using the research results from the second part of the experiment as representative for the overall evaluation of the ethical stories constructed in our experiment by the young students.

Therefore, the authors of this study decided to select two other group of young people applying to study psychology for the second part of the experiment, i.e. for overall evaluation of the ethical stories (modeled in the first part of the experiment) as the subjects who are just after their maturation and participate in the entrance exams for psychology at the University of Trnava. These subjects are called Subject Group 2. Their number is 450 (N=450).

RESULTS

The research findings from this moral reasoning experiment are presented according to the two phases of this experiment: (1) the results of modeling ethical stories, and (2) the results of the overall moral evaluation of the modeled ethical stories (see: the example of the *Protocol of Experimental Modeling of Ethical Stories* and its completed *Moral Evaluation* back to the experimenter (see: APPENDIX 2).

The modeling phase

The total number of protocols received with completed modeling was 536. This means that the main result of our findings from the modeling phase of our experiment on moral reasoning is a systematized empirical data base of ethical stories, which is a kind of empirical description of characteristics of denotative meaning of the indicators of morality understood in their positive, negative, or neutral meaning in a context of cognitive schemas of ethical stories. This base is systematized in accordance with the order numbers of the 54 IAO and KCR cognitive schemas, sequenced and presented as a matrix in Table 6. One way of presenting this data base to list the modal examples of modeling created on the base of this matrix. The full list of modal models can be found in the original Slovakian version of the report by Grác (2008, pp 119-125) and the Polish version by Grác (2015, pp.140-148).

This data base can be a source for qualitative analysis on the structuring of moral reasoning using indicators of morality. One of the opportunities for research using this data base points is to use the schemas for research on overall assessment of the moral value of the ethical stories. This is what was done in the second phase of this research project.

The moral evaluation phase

We will now show what kind of cognitive strategies the subjects developed in their overall moral evaluations, based on analyzing the values of indicators of morality as premises in inferring the conclusions in the IAO and then KCR schemas.

Strategies used to complete overall moral evaluations of the IAO schemas of ethical stories

The students' overall moral evaluations of the IAO ethical stories allowed us to identify three regularities which describe their moral reasoning in analyzing the values of the indicators of morality in these stories. These regularities are also intuitively deducible from the syntactic structure of the indicators of morality contained in the structures of formal ethical story schemas used in our experiment. These syntactic regularities are referred to as the model strategies of overall evaluations of ethical stories.

The moral strategies of overall evaluations of IAO ethical stories

The first regularity can be called the *principle of univocal values of intention and application indicators*. The matrix which indicates the overall moral evaluations which fit this regularity is shown in Table 7.

Table 7. Global moral evaluations of the IAO schemas in accordance with the principle of univocal values of intention and application indicators

Order number of the schema	IAO schema	Overall moral evaluation	Order number of the schema	IAO schema	Overall moral evaluation
1	(+ + +)	[+]	12	(∅∅∅)	[∅]

4	(+ + -)	[+]	13	(- - +)	[-]
7	(+ + ∅)	[+]	23	(∅∅ -)	[∅]
8	(- - -)	[-]	25	(∅∅ +)	[∅]
11	(- - ∅)	[-]			

The analysis of Table 7 allows us to formulate *a principle of univocal values of intention and application indicators* as follows:

$$(I = A) \Rightarrow (ES = I = A) \qquad (15),$$

where: ES denotes the overall moral evaluation of ethical story; I and A denote the appropriate signs of moral values of the indicators in IAO schemas.

The second regularity of the overall evaluation of the IAO schemas of ethical stories is called the *principle of preferring application (A) indicator over intention indicator (I)*. Table 8 shows the matrix indicating the overall moral evaluations which fit this principle.

Table 8. Overall moral evaluations of IAO schemas in accordance with the principle of preferring application (A) indicator over intention indicator (I).

Order number of the schema	IAO schema	Overall moral evaluation	Order number of the schema	IAO schema	Overall moral evaluation
2	(+ - +)	[-]	16	(∅ + -)	[+]
3	(- + +)	[+]	17	(- + ∅)	[+]
6	(∅ + +)	[+]	18	(∅ - +)	[-]

10	(∅ - -)	[-]	20	(+ - ∅)	[-]
14	(- + -)	[+]	22	(∅ - ∅)	[-]
15	(+ - -)	[-]	26	(∅ + ∅)	[+]

From Table 8 we can learn that *the principle of preferring application (A) indicator over intention indicator (I)* may be formally expressed as:

$$(I \neq A) \Rightarrow (ES = A) \qquad (16),$$

where the symbols denote the same as in (15).

The third regularity says that if the intention indicator is non-zero, and the sign of the application indicator is zero, the concluded overall moral evaluation of the ethical story is formally expressed by the sign of the intention indicator, independently of the sign of the outcome indicator. This regularity is called as the *principle of preferring the non-zero intention indicator (I) over the zero application indicator (A)*. The designates of this principle are shown in the matrix presented in Table 9. Might this be related to Kant from earlier?

Table 9. Overall moral evaluations of IAO schemas in accordance with the principle of preferring the non-zero intention indicator (I) over zero application (A) indicator.

Order number of the schema	IAO schema	Concluded moral evaluation	Order number of the schema	IAO schema	Concluded moral evaluation
5	(+∅ +)	[+]	21	(+∅ -)	[+]
9	(-∅ -)	[-]	24	(-∅∅)	[-]

19	(-∅ **+)**	[-]	27	(+∅∅**)**	[+]

Analysis of the patterns of indicator signs allows us to formulate in a formal way the principle of preferring the non-zero intention indicator (I) over the zero application indicator A expressed as:

$$[(I \neq \varnothing) \cap (\mathbf{A} = \varnothing)] \Rightarrow (ES = I) \qquad (17).$$

Representativeness of the syntactic models in overall evaluations of IAO ethical stories

At what level are, the above-presented models of overall evaluations of IAO ethical stories, descriptive for the subjects? Overall evaluations of IAO ethical stories extracted from the empirical data base were carried out, having been obtained in the modeling part of the experiment and prepared in the ZEU-IAO Test for further research. The participants of this part of the experiment (N=450) were high school graduates who took their entrance exams for psychology at the University of Trnava (Slovakia). In 93.75% of the IAO ethical stories schemas, the modal evaluation of the subjects turned out to be consistent with the model strategy of logic-formal syntax. The percentages of these strategies ranged from 28.70% to 98.00%.[2] If it is worth mentioning, then you should make this clear here.

These results were consistent with a study carried out three years later with another group of high school graduates (N = 355) participating in the entrance exams for psychology at the same university. They were tested with the same ZEU-IAO test as the group of their colleagues earlier. Both study groups had no contact with each other in the interim. The second study showed that for 93.75% of the IAO ethical stories schemas, the modal evaluation of the subjects turned out to be consistent with the model strategy of logic-

[2] See: Grác (2008) Chapter VI, pp.135-145, Table 15.

formal syntax indicated in Tables 7-9 and in Formulas (15)-(17), respectively. This means that the strategy of integrating the morality indicators of moral stories into IAO schemes resulted in highly descriptive models for the processes of moral evaluation of these stories. The percent of subjects employing these strategies in the experimental ethical stories ranged from 32.00% to 98.30%.[3]

Strategies used to complete overall moral evaluations of the KCR schemas of ethical stories

The first main finding concluding the moral evaluation of the ethical stories within the KCR schemas is that only concordance (C) and realization (R) indicators of morality played a decisive role in the overall moral evaluations of the stories. Familiarity with the heroes of the stories of moral norms (K) appeared only to play a contextual role in the ethical stories, with no impact on completing the moral evaluation process.

The students' assessment data allowed us to identify the following four strategies in concluding the moral evaluations of the KCR schemas of ethical stories:

1. If the sign of the concordance indicator (C) is univocal with the sign of the realization indicator (R), the sign of the concluded moral evaluation of the ethical story is equal to the signs of both indicators in the premises of this conclusion, which can be formally expressed as:

$$(C = R) \Rightarrow (ES = C = R) \qquad (18),$$

where: ES – denotes the same as in the above formulas, and C, R denote the symbols of the KCR schemas.

[3] See: Grác (2008) Chapter VII, pp.155-178, Table 21.

2. If the sign of the concordance indicator (C) contradicts the sign of realization indicator (R), the overall moral evaluation is expressed with the sign ∅. As a formal expression it is stated as:

$$(I \neq R) \Rightarrow (ES = \varnothing) \qquad (19)$$

3. If the sign of the concordance indicator (C) is neutral (∅),the overall concluded moral evaluation of the ethical story is neutral as well, which one can formulate as:

$$(C = \varnothing) \Rightarrow (ES = \varnothing) \qquad (20)$$

4. If the sign of the realization indicator (R) is neutral (∅),the overall moral evaluation of the ethical story takes its value from the non-zero evaluation of the concordance indicator (C). In a formal way this regularity may be expressed as:

$$(R = \varnothing) \Rightarrow (ES = C) \qquad (21)$$

Representativeness of the syntactic models in overall evaluations of KCR ethical stories

In this section we indicate to what extent the integration stretches of morality indicators in the overall evaluation of ethical stories according to the KCR scheme (formulated in Tables 10-13 and Formulas (18) - (21)) constitute descriptive models for students participating in the experimental evaluation of these stories. To do so, we use the test results of a group of high school graduates (N = 450) who participated simultaneously in the test (both the ZEU-IAO Test and the ZEU-KCR Test). Based on the general data for the degree of compliance between the empirical distribution of moral valuation of the respondents for the tested ethical stories and the indications of the models discussed above, it can be said that in 10 test tasks out of all 16 tasks displayed for the KCR scheme, model strategies turned out to be descriptive for the evaluation of the studied group of high school

graduates. This means that in 62.50% of evaluation tasks, the modal number of respondents in 10 out of 16 groups of tasks, was consistent with the logical-formal syntax model. A more detailed presentation of this research situation is indicated in Table 22.[4]

Table 22. Percentage indicators of modal evaluations of moral stories KCR for the choices of respondents in relation to model indications

The order of the ethical story in ZEU-KCR	Values of morality indicators in the K C R	Overall evaluation of the moral story	Modal percentages of model evaluations*	Modal percentages of non-model evaluations
01	(+) (+) (+)	[+]	**79.4%**	
02	(+) (-) (-)	[-]	**49.2%**	
03	(-) (∅) (-)	[∅]	**64.0%**	
04	(+) (-) (+)	[∅]	36.4%	58.2% [-]
05	(-) (∅) (+)	[∅]	**50.9%**	
06	(-) (∅) (+)	[∅]	**70.2%**	
07	(+) (+) (+)	[+]	**89.0%**	
08	(+) (-) (-)	[-]	**89.2%**	
09	(-) (∅) (-)	[∅]	**68.3%**	
10	(+) (-) (+)	[∅]	27.0%	68.6% [-]
11	(+) (-) (-)	[-]	**70.2%**	

[4] Tabela 22 została sporządzona w oparciu o dane z Rozdziału IX: Grác (2008, pp. 208-2012).

12	(+) (+) (-)	[∅]	15.8%	82.4% [-]
13	(+) (-) (+)	[∅]	44.7%	49.4% [-]
14	(+) (-) (-)	[-]	**86.1%**	
15	(+) (-) (+)	[∅]	36.5%	61.5% [+]
16	(+) (+) (-)	[∅]	19.8%	75.2% [-]

* The bolded percentages in the column fourth of Table 22 denote that these modal evaluations by the subjects fit in the defined ethical stories of the model strategies related to these stories.

The data from Table 22 allow us to more accurately conclude that the modal percentages of model evaluations are quite varied with respect to individual moral stories exposed in the experiment: from 49.2% to 89.2% of the respondents' modal moral evaluations.

CONCLUSION

In this section we will attempt to respond to the question of which mental processes are involved in both the modeling phase of our experiment and the overall evaluation of the ethical story. Grác (2008, 2015) assumes that in modeling ethical stories, semantic operations are involved to create thematic-oriented structures in accordance with the sequences determined by the formal signs of the indicators of morality which constitute the cognitive schemas of potential stories (moralities). We can only add that these semantic mental operations must be focused both on securing particular sequences for the appropriate moral valuation required by the cognitive schemas obtained in the experiment, and, at the same time, on maintaining the thematic coherence of the content of the whole ethical story.

We consider here the role in moral reasoning played by the particular indicators of morality known from the literature, such as motivation and intension (Moore, 2005; Chih-Feng et al., 2016). In the overall evaluation phase of the experiment, moral reasoning required more syntactic and formal logic cognitive operations. Grác distinguishes three processes that enabled the subjects to formulate the regularities in concluding the overall moral evaluation of ethical stories: *selection, differentiation* and *integration*.

These are "cognitive" processes?

The *selection process* deals with highlighting the indicators of morality that do not influence moral evaluation of ethical stories, but only create a situational context in these stories. These indicators appeared to be: the outcome indicator (O) in the IAO schemas, and the knowledge indicator (K) in the KCR schemas. This means that these two indicators are not considered as premises in completing overall moral evaluation of ethical stories. The result of the *differentiation process* is in distinguishing among the indicators of morality which play a dominant role in competing the overall moral evaluation of moral stories, and those which are only connected with this evaluation. The dominant positions were taken by: the application indicator (A) in the IAO schemas and the concordance indicator (C) in the KCR schemas.

So, people are natural Kantians?

The main mental process related to conducting an overall moral evaluation is *integration*, which allows a generalizing value to be assigned for each group of three indicators in the IAO and KCR schemes. As a result of this process, seven regularities were identified that describe the generalized patterns of indicator sign in the premises and the inferred overall conclusion for each group of ethical stories. The relationship discovered between each of the particular groups of indicator signs as premises and the overall moral evaluations of the moralities constitutes a psychological connection for moral inference in

our experiment on moral evaluations of ethical stories. Formally, this relationship can be expressed in the formal logic syllogism *modus ponendo ponens* as follows:

$$[(p \Rightarrow q) \cap p] \Rightarrow q \qquad (22),$$

where the symbols of inference connection (p⇒q) denote the particular regularities formulated in (17)-(21) as the first premise for conducting the moral evaluation, where *p* denotes the conditional sentence concerning the particular group of indicators signs of moral valuation and *q* denotes the result of the overall moral evaluation of ethical story.

The above discussion statement corresponds with A. Biela's (1986) commentaries on R.J. Sternberg's (1984) triarchic theory of human intelligence and his positive response to these comments. Starting from the statement that higher levels of moral development are related to higher levels of cognitive development, Biela suggested that it is possible to include moral/axiological level to a theory of human intelligence without any decrease of its empirical testability (cf. Williams and Gantt, 2012). The presented study on moral reasoning evidently supports this suggestion.

Typically it is not advised to introduce new material in the concluding paragraph. Maybe this sort of background could be useful in setting up the purpose of the work, here?

Final Remarks

In conclusion, we have described the novelty of the two phase experiment conducted on moral reasoning, which included a moral story modeling phase and an overall moral evaluation phase developed by educational psychology students.

The moral story modeling phase developed by the students gave them the chance to learn what moral reasoning about youth behavior is in an attractive way, and what role the indicators of morality of their experimentally modeled ethical stories play in overall moral assessment of human performance. The modeling phase of the experiment enabled the subjects to take a large step towards the process of internalization of moral norms regulating

human behavior in social situations. In addition, this process was of a multidimensional nature and a structured character, leading to the overall moral evaluation of human action.

In this experiment, students were able to create models of complex matter, using the indicators of moral evaluation of their own youth social performance. Thus, we may say that this experiment took place in closer proximity to real-life thinking processes than any other research method previously used in experimental psychology.

Financial Support / Funding Statement: This research received no specific grant from any funding agency, commercial or not-for-profit sectors.

Conflicts of Interest: None

References

Awad, E., Bonnefon, J., Shariff, A., & Rahwan, I. (2019). The Thorny Challenge of Making Moral Machines: Ethical Dilemmas with Self-Driving Cars, NIM Marketing Intelligence Review , 11(2), 42-47. doi: https://doi.org/10.2478/nimmir-2019-0015

Alfano, M. (2019). Moral reasoning is the process of asking moral questions and answering them. Behavioral and Brain Sciences, 42, E147. doi:10.1017/S0140525X18002534

Biela, A. (1986). Toward a synthetic approach to intelligence. Behavioral and Brain Sciences, 9(3), 578-579. doi:10.1017/S0140525X00047270

Biela A. (2015). Przedmowa do wydania polskiego. In J. Grac, Psychologiczne analizy procesów interioryzacji norm moralnych. Lublin: Towarzystwo Naukowe KUL.

Bucciarelli, M., Khemlani, S., & Johnson-Laird, P. N. (2008). The psychology of moral reasoning. *Judgment and Decision Making*, 3(2), 121–139.

Calmeiro, L., Stoll, S. K., & Davis, P. (2018). Moral Reasoning in Sport: Validation of the Portuguese Version of the RSBH Value-Judgement Inventory in Adolescents. Sport Science Review, 24(5–6), 285–304. https://doi.org/10.1515/ssr-2015-0020

Candee, D. (1976). Structure and choice in moral reasoning. Journal of Personality and Social Psychology, 34(6), 1293–1301. https://doi.org/10.1037/0022-3514.34.6.1293

Chih-Feng Chien, Ching-Jung Liao, Brent G. Walters, & Ching-Yieh Lee. (2016). Measuring the Moral Reasoning Competencies of Service-Learning e-Tutors. Journal of Educational Technology & Society, 19(3), 269-281.

Choi, Y. J., Han, H., Dawson, K. J., Thoma, S. J., & Glenn, A. L. (2019). Measuring moral

reasoning using moral dilemmas: evaluating reliability, validity, and differential item functioning of the behavioural defining issues test (bDIT). European Journal of Developmental Psychology, 16(5), 622–631. https://doi.org/10.1080/17405629.2019.1614907

Crockett, M. J. (2013). Models of morality. Trends in Cognitive Sciences, 17(8), 363-366. https://doi.org/10.1016/j.tics.2013.06.005

Cushman, F., Young, L., & Hauser, M. (2006). The role of conscious reasoning and intuition in moral judgment: Testing three principles of harm. Psychological Science, 17(12), 1082–1089. DOI: 10.1111/j.1467-9280.2006.01834.x

Czyżowska D., Niemczyński A. (2006) Universality of Socio-moral Development: a cross-sectional study in Poland, Journal of Moral Education, 25:4, 441-453. DOI: 10.1080/0305724960250405

Dewey J. (1910). How We Think. Boston: D. C. Heath & Co., Publishers.

Ellemers, N., van der Toorn, ., Paunov, Y. & van Leeuwen, T. (2019). The Psychology of Morality: A Review and Analysis of Empirical Studies Published from 1940 through 2017, Personality and Social Psychology Review, 23 (4), 332-366. https://doi.org/10.1177/1088868318811759

Fang, Z., Jung, W. H., Korczykowski, M., Luo,L., Kristin Prehn,K., Xu, S., Detre,J., A., Kable, J. W., Robertson, D. C., & Rao, H. (2017). Post-conventional moral reasoning is associated with increased ventral striatal activity at rest and during task. Scientific Reports, 7 (1). DOI: 10.1038/s41598-017-07115-w

Forte, A. Business Ethics: A Study of the Moral Reasoning of Selected Business Managers and the Influence of Organizational Ethical Climate. Journal of Business Ethics 51, 167–173 (2004). https://doi.org/10.1023/B:BUSI.0000033610.35181.ef

Gehrlein, J., Crede, A. K., & Adrian, N. (2020). The impact of markets on moral reasoning: Evidence from an online experiment. Journal of Behavioral and Experimental Economics , 87, 101577. https://doi.org/10.1016/j.socec.2020.101577

Gibbs, J. C. (2006). Should Kohlberg's cognitive developmental approach to morality be replaced with a more pragmatic approach? Comment on Krebs and Denton. Psychological Review, Vol. 113(3), July, 666-671. DOI: 10.1037/0033-295X.113.3.666

Grac J. (2008). Psychologia mravnosti v teoretickych a empirickych analizach. Trnava: Filozoficka fakulta Trnavskej univerzity.

Grac J. (2015). Psychologiczne analizy procesów interioryzacji norm moralnych. Lublin: Towarzystwo Naukowe KUL.

Greene, J. D., Cushman, F. A., Stewart, L. E., Lowenberg, K., Nystrom, L. E., & Cohen, J. D. (2009). Pushing moral buttons: The interaction between personal force and intention in moral judgment. Cognition, 111(3), 364–371. DOI: 10.1016/j.cognition.2009.02.001

Kamali, F., Yousefy, A., & Yamani, N. (2019). Design and psychometric properties of an instrument to assess metacognition in moral reasoning in medicine. Nursing Open, 6(4), 1331–1345. https://doi.org/10.1002/nop2.331

Kohlberg, L. (1969). Stage and Sequence: The Cognitive Developmental Approach to Socialization. In D. Goslin, (Ed.), Handbook of Socialization Theory and Research (347-480). Chicago, IL: Rand McNally.

Kohlberg, Lawrence (1971). From Is to Ought: How to Commit the Naturalistic Fallacy and Get Away with It in the Study of Moral Development. New York: Academic Press.

Kohlberg, L. (1976). Moral stages and moralization: The cognitive developmental approach. In T. Lickona (Ed.),Moral development and behavior: Theory, research and social issues (31-53). New York, NY: Holt Rinehart and Winston.

Kohlberg, L., Candee, D. (1984). The relationship of moral judgment to moral action. In L. Kohlberg (Ed.), Essays in moral development: Vol. 2. The psychology of moral development (pp. 498 –581). New York: Harper & Row.

Hanna, L. A., Gillen, J., & Hall, M. (2017). An investigation of final year pharmacy students' moral reasoning ability, and their views on professionalism and fitness to practice panel determinations: A questionnaire study. Currents in Pharmacy Teaching and Learning, 9(4), 652–659. DOI: 10.1016/j.cptl.2017.03.016

Millar, J. (2014). The Tunnel Problem, *Practical Ethics*, http://blog.practicalethics.ox.ac.uk/contact/

Moll, J., & de Oliveira-Souza, R. (2007). Moral judgments, emotions and the utilitarian brain. Trends in cognitive sciences, 11(8), 319–321. https://doi.org/10.1016/j.tics.2007.06.001

Moore G. E. (2005). Ethics. Oxford, Oxford University Press.

Nikolaicev, B. O. (1976). Osoznavaemoe i neosoznavaemoe v nravstvennom povedenii licnosti. Moskva: Izdavatelstvo Moskovskogo universiteta.

Paciello, M., Fida, R., Tramontano, C., Cole, E., & Cerniglia, L. (2013). Moral dilemma in adolescence: The role of values, prosocial moral reasoning and moral disengagement in helping decision making. European Journal of Developmental Psychology, 10(2), 190-205. https://doi.org/10.1080/17405629.2012.759099

Patil, I., Zucchelli, M. M., Kool, W., Campbell, S., Fornasier, F., Calò, M., … Cushman, F. (2020). Reasoning Supports Utilitarian Resolutions to Moral Dilemmas Across Diverse Measures. Journal of Personality and Social Psychology, (January). https://doi.org/10.1037/pspp0000281

Piaget, J. (1932). The Moral Judgment of the Child. The Free Press, New York.

Piaget J (1957). The construction of reality in the child. New York: Basic Books.

Piaget J. (1976) Piaget's Theory. In B. Inhelder, H. H. Chipman, C. Zwingmann (Eds.), Piaget and His School. A Reader in Developmental Psychology (11-23). New York, Berlin, Heidelberg: Springer-Verlag New York Inc.

Rest J. R. (1973). The hierarchical nature of stages of moral judgment. Journal of Personality, 41 (1), 86-109. https://doi.org/10.1111/j.1467-6494.1973.tb00662.x

Rest J. R. (1975). Recent Research on an Objective Test of Moral Judgment: How the Important Issues of a Moral Dilemma Are Defined. In D. J. DePalma, M. Foley (Eds.), Moral Development. Current Theory and Research (75-93). Hillsdale, New Jersey: Lawrence Erlbaum Associates, Publishers.

Rhim, J., Lee, G. bbeum, & Lee, J. H. (2020). Human moral reasoning types in autonomous vehicle moral dilemma: A cross-cultural comparison of Korea and Canada. Computers in Human Behavior, 102(August 2019), 39–56. https://doi.org/10.1016/j.chb.2019.08.010

Roche, C., Thoma S. (2017). Insights From the Defining Issues Test on Moral Reasoning Competencies Development in Community Pharmacists. American Journal of Pharmaceutical Education; 81 (8) Article 5913, 21-32. DOI: https://doi.org/10.5688/ajpe5913

Shou, Y., & Song, F. (2017). Decisions in moral dilemmas: The influence of subjective beliefs in outcome probabilities. Judgment and Decision Making. 12(5), 481–490.

Sternberg, R.J. (1984).Toward a triarchic theory of human intelligence, Behavioral and Brain Sciences, 7(2), 269-289. doi:10.1017/S0140525X00044629

Sternberg, R.J. (1986). Alternatives to the triarchic theory of intelligence, Behavioral and Brain Sciences, 9(3), 581-583. doi:10.1017/S0140525X00047300

Is this discussed anywhere but in passing in the concluding para?

Sudić, M., Valerjev, P., & Ćirić, J. (2019). Deontic moral reasoning task: Is moral reasoning special? Psihologijske Teme, 28(3), 483–506. https://doi.org/10.31820/pt.28.3.2

von Grundherr, M., Geisler, A., Manuel Stoiber, M. & Schäfer, M. (2017). School Bullying and Moral Reasoning Competence, Social Development 26 (2), 278-294. https://doi.org/10.1111/sode.12199

Williams R. N., Gantt E. E. (2012). Felt moral obligation and the moral judgment–moral action gap: toward a phenomenology of moral life. Journal of Moral Education Vol. 41, No. 4, December, 417–435. https://doi.org/10.1080/03057240.2012.665587

Wilmoth G. H., McFarlang S. G. (1977). A Comparison of Four Measures of Moral Reasoning. Journal of Personality Assessment, 41, 4, 396-401. https://doi.org/10.1207/s15327752jpa4104_11

Figure Click here to access/download;Figure;Figure.docx

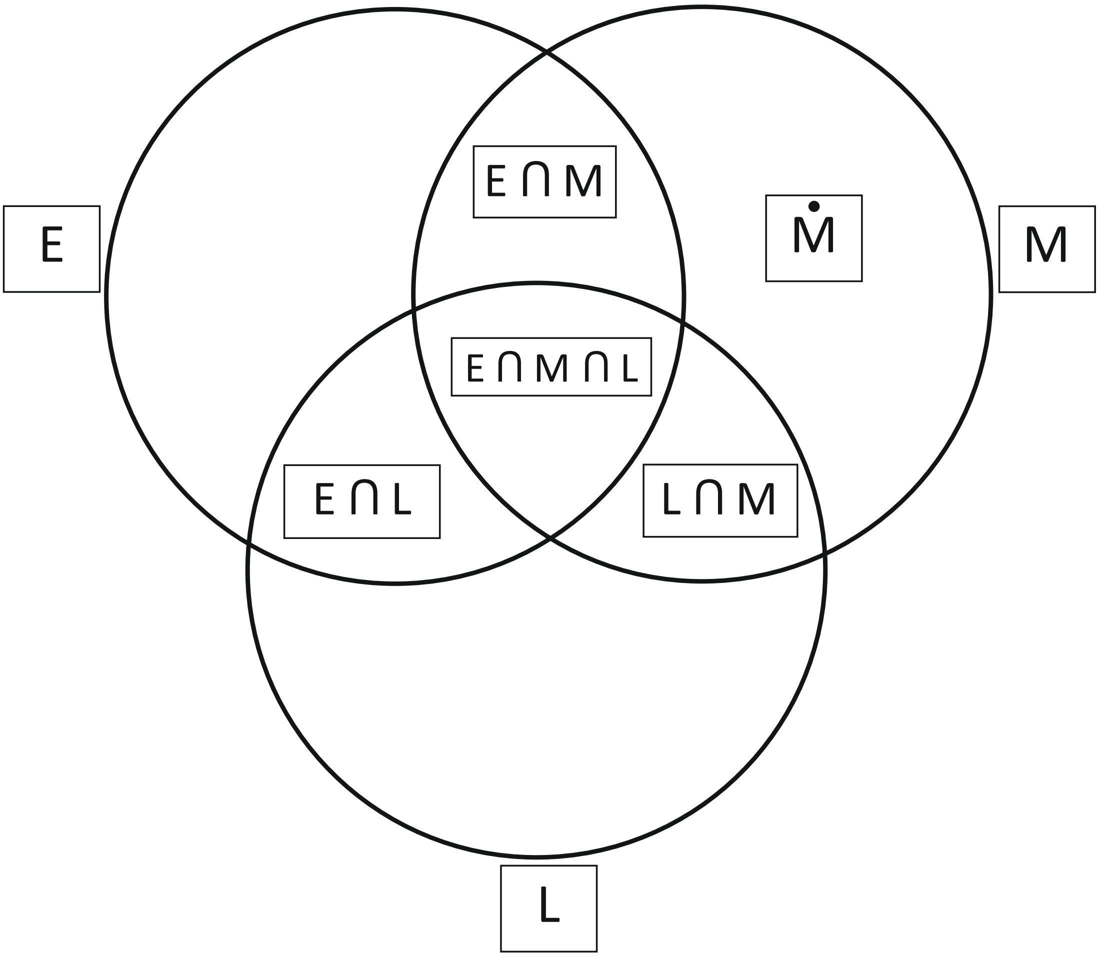

Figure 1. The internormative extensional relations between the executive, moral and legislative norms.

Click here to access/download
Supporting Information
APPENDIX 1.docx

Can moral reasoning be modeled in an experiment?

Ján Grác

Department of Psychology

Trnava University

Hornopotočná 23, 918 43 Trnava, Slovakia

e-mail: grac.j.z.2@pobox.sk

Adam Biela

Department of Social Psychology

Maria Sklodowska-Curie University

ul Głęboka 44, 20-612 Lublin, Poland

e-mail: adam@biela.pl

ORCID ID: 0000-0001-5730-7693

Piotr Mamcarz - Corresponding author

Department of Emotion and Motivation Psychology

The John Paul II Catholic University of Lublin

Al. Racławickie 14, 20-950 Lublin, Poland

e-mail: pmamcarz@kul.pl

Phone. +48 509 630 598 Fax +48 81 445 33 40

ORCID ID: 0000-0001-6307-6326

Dorota Kornas-Biela

Department of Psychopedagogy

The John Paul II Catholic University of Lublin

Al. Racławickie 14, 20-950 Lublin, Poland

e-mail: dorota@biela.pl

ORCID ID: 0000-0001-6002-1901

Can moral reasoning be modeled in an experiment?

Abstract

A review of the literature on moral issues indicates that none of the empirical approaches to moral reasoning proposes an experimental approach which controls for such object-related experimental variables as: motivation, acceptance of moral norms and consequences of human behavior in moral situations in a single research procedure.

A unique element of the proposed experimental method is a multi-stage model determining morality indicators. In the two-phase design experiment, 147 educational psychology students were asked to create model ethical stories and then conduct an overall assessment of each of these stories.

As a result, a base of ethical stories was created with empirical moral indicators (positive, negative, neutral). The patterns in the moral evaluation of ethical stories were determined by identifying three processes (selection, differentiation and integration). The final result is a confirmed design of the experiment and a set of formulas that can be used in education and research on morality reasoning.

Key words: moral reasoning; decision making; experiment; moral indicators~~cognitive schemas of ethical-dilemma-stories; modeling of ethical stories; moral reasoning experiment; morality indicators; overall moral evaluation of ethical stories; synthetic approach to intelligence.~~

INTRODUCTION

In our study, we present an experimental approach to the study of moral reasoning which allows for the control of some indicators of morality as independent variables. A research proposal for this new approach was first presented by the Slovak educational psychologist Jan Grác (2008). This proposal will be presented first in the context of the classic approaches to moral reasoning, and then in the latest contemporary research, with particular emphasis on the opportunities for experimental exploration of this research field.

Empirical studies on moral reasoning are well known in the psychological and educational literature (Rest, 1975; Candee, 1976; Czyżowska, Niemczyński, 2006; Joyce, 2006; Williams & Gantt, 2012; Bucciarelli, Khemlani, & Johnson-Laird, 2008; Alfano, 2019; Ellemers et al., 2019). Most of them are inspired by the two early concepts on structure and choice in moral reasoning offered by Piaget (1932, 1957) and Kohlberg (1969, 1971). More recent studies on moral thinking are related with the concept of *moral intelligence* and its developments (see: Kornas-Biela, Schwarz, Biela-Wołońciej, 2018; Kornas-Biela, 2019).

Classic approaches to moral reasoning

Piaget (1932, 1957) began his research on moral development using the clinical-experimental approach. This approach was based on short dialogues with individual small children in the context of a moral issue situation. The dialogue with a child, called an *indagation*, aimed to ascertain his or her individual understanding of moral concepts. Piaget proposed a model of stages of moral reasoning which states as one of its premises that

qualitative changes in the structure cause the appearance of a new stage at specific points in time. It could be said that he began a methodological revolution, which shifted psychologists' orientation from analyzing an individual`s behavior or choice to the underlying form and structure of moral reasoning. Piaget's research approach was a kind of transposition of the maieutic method of Socratic dialogues into the situation of a conversation with a young child on moral issues. He created a non-quantitative but qualitative and creative method of scientific exploration of moral thinking in young children. The creative value of Piaget's clinical experiment was used by Grác (2008, 2015) to create a phase of the experiment called modeling ethical stories by students.

Another approach by Kohlberg (1969, 1971) is based on hypothetically constructed moral dilemmas; it aims to test moral decision-making in youth and adults. Kohlberg traced the development of moral structures through a series of six stages which form an invariant and culturally universal sequence (Gibbs, 2006). Moral structures are measured by requesting individuals to reason about cases in which two or more conventional values are in conflict. The subject reacts to a moral dilemma, indicating what ought to be done and justifying this course of action. The interviewer tries to elicit and probe the subject`s views without interjecting or suggesting any thinking different from the subject`s own spontaneous thinking. The subjects' behavior is interpreted in terms of competence levels of moral reasoning (Roche and Thoma, 2017).

Recently, new authors in psychological investigations of moral reasoning have frequently employed moral dilemmas (e.g. Shou & Song, 2017; Crockett, 2013; Paciello et al., 2013; Greene et al. 2009) ; Cushman, Young & Hauser, 2006;; Greene, Morelli, Lowenberg, Nystrom & Cohen, 2008; Haidt, 2007; Moll & de Oliveira-Souza, 2007).

Restrictions experienced in the measurement of the structure of moral reasoning has inspired researchers to search for some standardized, objectively scorable tools of moral judgment. A significant contribution in this regard was made by Rest (1973), who Studying how people choose important issues in moral dilemmas, he and his colleagues invented a procedure called the Defining Issues Test (DIT) which requires a subject to read a hypothetical moral dilemma and then select those statements that are the most important issues in making a decision about the case from among 12 statements. The research he conducted, however, confirmed that difficulties still exist in the assessment of moral judgment with an objective test (Rest, 1975; Wilmoth and McFarlang, 1977). That is why Wilmoth and McFarlang (1977) carried out a comparative analysis of psychometric values of the psychometric tools which measure moral reasoning.

Insights from the *Defining Issues Test* on moral reasoning competencies was taken into some applied fields (e.g. in service-learning e-tutors by Chien et al., 2016; educational intervention in community pharmacists by Roche & Thoma, 2017). HoweverThus, the question remains as to whether it is sufficient and appropriate to study moral reasoning using only a standardized test with structured responses (Fang, 2017).

The problem of ethical valuation of moral behavior

Some theories of moral development (cf. Kohlberg, 1971; Gibbs, 2003) focused on moral reasoning describe the way in which a person understands and interprets social situations and directs themselves when it comes to solving moral dilemmas. The relationship between moral reasoning and moral action is complex. Kohlberg represented the position

that moral judgment is a necessary but not always sufficient condition for moral action (Kohlberg & Candee, 1984). More recent studies show that competence of moral reasoning, understood in terms of the Kohlberg's theory as a measure of moral development, correlates negatively with bullying at school in youngsters (von Grundherr et al., 2017). In turn, Fang et al. (2017) found that the highest level of moral reasoning is associated with increased ventral striatal activity at rest and during task in students.

Analyzing the logical foundations of Kohlberg's research paradigm (e.g.,Candee, 1976; Kohlberg & Candee, 1984; von Grundherr et al., 2017) on hypothetical moral dilemmas, one can draw the conclusion that research on moral evaluation is itself dilemmatic, primarily because of the one-dimensionality in which the dilemmas in moral judgement literature continue to be formulated. In his research approach, Kohlberg could not apply the experimental procedure in a strict manner so that the moral norms present in the tests of his dilemmas would be controlled as independent variables having a hypothetical influence on the moral reasoning contained in the psychometric tests. In his research on moral reasoning, he chose to expose the subjects to standardized tests of moral dilemmas, where the stimuli and test conditions had to remain constant by definition (*ex defintione*). In our experimental study based on Grác's methodology (2008, 2015), ethical stories will be presented as a multidimensional reality, which allows us to take a new research approach to moral reasoning. In this study, the indicators of morality as an important context of moral evaluation will be treated as independent variables, the influence of which are strictly controlled by the experimenter.

A motivating contribution to understanding moral reasoning was introduced by British analytical philosophy, developed by the fertile minds of analytical philosophers such as G.E. Moore, A. N. Whitehead and B. Russell. They incorporated a multidimensional

approach in the analysis of moral assessment. For example, Moore (2005) claims that a human action (act) can be evaluated as morally good or bad only in the context of a given situation and the effect of the person's free choice.

Moore's analysis of psychological factors, which defines the intentions contained in the motives of the act and the feelings associated with it (i.e. experienced feelings), is a prerequisite for understanding moral judgment. In a review and synthesis of his analysis, Moore states that nothing can be inherently good or bad if does not contain some emotion. That is why Moore's ethical system is named *emotivism*.

According to Moore, a substantial value in the process of moral evaluation is the motivation of the act. A general psychological predisposition to right proceedings corresponds to good motives, although right proceedings are not always an effect of these motives. Before acting, a subject may have good intentions, but he or she is not able to predict the results of their decision. This is due to both the cognitive limitation of the human being and the risky nature of the decisive situation in which the individual is placed, where hypothetical states of reality constitute the set of uncertain events, for which we can only determine degrees of probability.

We now proceed to review the results of meta-analysis of the literature on moral reasoning, with particular emphasis on the experimental methodology of these approaches. Ellemers et al. (2019) presented a monumental meta-analysis of empirical studies on the psychology of morality published from 1940 through 2017.

Analogically to Moore's analysis, these authors distinguished the moral studies from the non-moral ones according to the criterion of considering in the research the problem of

~~what is right (morally good) vs. wrong (morally bad) way to behave. In the first stage of their metaanalysis they distinguished five relatively independent areas of morality research: moral reasoning, moral behavior, moral judgment, moral self-view, moral emotions. In the next stage, relationships between these areas were shown and types of measurement scales, which were used by the authors of the analyzed studies. This metaanalysis enabled to draw the two conclusions: (1)~~

~~different life experiences and social roles can have a long-term impact on the way people reason about morality and the moral principles they prioritize; (2) more immediate situational cues also affect moral reasoning and moral decisions. These conclusions on moral reasoning complement philosophical analyzes as well as evolutionary approaches, highlighting the objective value of survival morality by following specific principles or guidelines (Shou and Song, 2017).~~

Meta-analysis outcomes on moral reasoning research approaches

What are the methodological conclusions from this meta-analysis regarding experimental research on morality? The authors (see: Ellemers et al., 2019) clearly indicate that studies on morality have largely neglected to examine how moral reasoning relates to actions in experimental contexts, such as moral norms and intentions.

Our more recent meta-analysis of moral reasoning studies has allowed us to state that prior research has focused on the following three points: (1) operationalization of moral reasoning abilities by constructing the psychometric tests and measuring their validity as well as reliability (Choi et al., 2019; Patil et al., 2020); (2) inventing experimental approaches on moral reasoning: laboratory experiment (Sudić & Ćirić, 2019), on-line experiment (Adrian & Gehrlein, 2020), and moral reasoning thought experiment (Rhim, Lee G. & Lee, J., 2020); and (3) applied research on moral reasoning in some particular professional domains where

morality and ethics is considered to be an important part of professional competencies, such as health care professionals (pharmacists, e.g. Hanna, Gillen & Hall, 2017; Göttgens-Jansen et al., 2019; nurses, e.g., Kamali, Yousefy, & Yamani, 2019), businessmen (Forte, 2006); sportsmen (Calmeiro, Stoll, & Davis, 2015), and computer scientists (Rhim, Lee G. & Lee, J., 2020).

Only one point of our meta-analysis outcomes, i.e. (2), signals an interest in experimental research on moral reasoning, where all the above-mentioned authors present the results of research that include the paradigm of moral dilemmas. Thus, Sudić & Ćirić (2019) designed the laboratory experiment of 3x2x2 within groups, where the independent variables were: a) content of norm (moral, conventional, or abstract), b) rule type (obligation, or permission), and c) induced dilemma (punishment dilemma, or reward dilemma). Three dependent variables were measured in the experimental task: response time, accuracy and final confidence of performance. One of the main experimental findings is that moral rules are easier to process than conventional ones, and conventional rules are easier than abstract ones. However, the authors indicate in their concluding remarks that they are unsure as to what degree conventional and abstract rules contaminated the moral ones. Moreover, this approach is limited because the 'correct' response defined by this experiment is only logical consistency, and it does not take into account real life moral reasoning. In conclusion, this experiment can be treated as a good initial exploration of the problematic multidimensional field of moral reasoning, which is difficult to study using the method of a laboratory experiment.

Authors of a second study (Adrian & Gehrlein, 2020) presented an online experiment investigating the influence of the market environment on moral reasoning. A control group of respondents was asked to make a choice regarding moral dilemmas, while the study

participants were exposed to either a market or non-market environment, and after that were asked to solve moral dilemma experimental tasks. While in principle online experiments are not objectionable, our main objection to this research is the fact that it does not consider morality indices as independent variables at all.

The third experimental approach to moral reasoning presented in our meta-analysis is a very intriguing thought experiment by Rhim, Lee & Lee (2020), who studied human moral reasoning types in an autonomous vehicle moral dilemma, in a cross-cultural comparison of Korean and Canadian subjects. These authors enrich our knowledge about the possibilities of simulation studies in the field of artificial moral intelligence regarding descriptive models of drivers' moral reasoning in problematic road traffic situations (e.g. Tunnel Problem (Millar, 2014); MIT Moral Machines (e.g., Awad et al., 2019).

~~2016)).~~ The study of moral reasoning in road traffic situations is not exhausted by dilemmas, however. It would be useful to consider the behavior of real drivers to a greater extent, to take into account moral indicators as factors influencing complex moral thinking processes. It is these more complex processes subject to manipulation in Grác’s experiments, which are featured in our study.

Summarizing the above research findings one can state the following outcomes, among others: (a) moral reasoning is a multi-faceted phenomenon where its mutually linked factors constitute the moral existence of the individual human person; (b) there is a need to distinguish and differentiate the following components of moral reasoning processes for both theoretical and practical purposes: cognitive, volitional, emotional, motivational (intentional) and behavioral processes – which the literature refers to as indicators of morality; (c) psychology is still far from determining the external validity of its psychometric

and experimental instruments for studying moral reasoning in natural experiment circumstances with a higher ecological validity.

In the context of the above statements, one can pose the question: Is it possible to design an experiment on moral reasoning in circumstances of higher ecological validity, and if so, in what way?

J. Grác's theoretical and methodological statements on moral reasoning

Unfortunately, none of the above approaches to moral reasoning propose any form of research which controls for object-related experimental variables, such as those related to motivation, acceptance of moral norms, and consequences of human behavior in moral situations – in a single research procedure. Grác states that this is possible, and we will demonstrate this in our ~~natural~~ experiment conditions, where subjects had as a task to define their real-life moral issue as a situation characterized by moral norms involved with the social-moral environment of their professional or educational endeavor. Such arrangements guarantee appropriate circumstances for significantly higher ecological validity of the experimental procedure.

Moreover, Grác (2008) assumes that moral reasoning is a process where the subject conducts a moral evaluation of a human event based upon some premises. The premises for him or her are the indicators of morality which are analyzed in the particular situations, and then synthetized as an overall moral assessment of their activity (or non-activity) under the given social environment. Thus, a quite intriguing research question is: What kind of strategies do people use in the process of integrating indicators of morality in a particular moral situation into an overall moral evaluation of the human activity that is assessed?

This novel approach to the evaluation of moral reasoning processes is proposed by J. Grác (2008, 2015), who has made a conceptual contribution towards the psychological analysis of the interiorization processes of moral norms. It is worthy of note that Grác in his original Slovak work (2008) distinguishes between the terms: *mravnost'*, and *moralnost'*. In the *Introduction to the Polish edition* of Grác's book, Biela (2015) emphasizes that these two terms only exist in discriminative usage in two contemporary languages, i.e., Czech and Slovak, and the term *mravnost'* is no longer used in Slavic languages other than Czech and Slovak. This certainly means that psychologically, the users of these two languages operate with more developed cognitive and behavioral structures for the moral domain than the users of other European languages. What differentiates the meanings of these two terms?

Grác (2015, p 71) states that the term "mravnost'" denotes a morally good performance as the highest ethical level of evaluation. It can be said that this is so because this term indicates the connection of morally good behavior with the accompanying positive psychological experiences, whereby the moral norm regulating both compounds of the conjunctive relationship: morally good behavior and the accompanying morally good experience – indicate a deep interiorization of the moral norm constituting the psychological basis of this conjunction. In turn, the term "moralnost'" according to Grác means the externally observed manifestations of morally good behavior without accompanying psychological experiences. Hence, not all performances observed as morally good are simultaneously "mravne" (i.e., moral), but only those where good performance is associated with morally good psychological experiences. Therefore, "mravnost'" is interpreted by Grác as an immanent psychological phenomenon.

What is the psychological novelty of Grác's conception of morality, and what is its methodological significance for an experimental approach to moral reasoning processes? Its

greatest novelty is in extending the conditions of assessment of human performance in the defined situation as morally good or bad. In order to define the criteria enabling such an evaluation, Grác distinguished two parallel analytical dimensions of human performance: (1) moral behavior, i.e. the external revealing of moral decision making (external dimension); (2) the internal experience related to the moral behavior, i.e. intentional focus on the moral norm, motivation (internal dimension). In this way, the psychological understanding of moral norms as factors regulating human performance is enhanced by experiential and behavioral structures, creating a basis for systematizing norms as moral, executive, and legal. Executive and legal norms regulate only externally revealed human performance. The characteristic of moral norms is that they regulate both human behavior associated with internal experiences focused on a particular moral norm as well as these internal experiences themselves (i.e. internal experience not tied with revealed behavior (Grác, 2008).

In this context, we can evaluate an individuals' ethical conduct in a specific situation as being a morally good action or a morally bad one. However, *morally good* vs. *morally bad* designates human behavior in a concrete situation, independent of whether the specific moral norm, e.g., hard work, courage, honesty, respect, trustworthiness, dedication, loyalty, has been actualized or not. Grác (2008) analysis posits that a person who accepts a particular moral norm but does not meet it could not be evaluated as performing a morally good action. And, similarly, a person could not be evaluated as having performed a morally good action if that person behaved in a manner consistent with the specific moral norm yet he or she did not accept this norm. Only the conjunction of the positive internal experience of the moral norm with the positive fulfillment of this norm in the situation provides the necessary and sufficient conditions for the positive ethical evaluation of a person acting in a given situation.

The mental processes involved in ethical evaluation of moral performance in everyday life situations and, particularly, in extreme situations are not passive ones on the part of the human subject; they involve active interaction with the social and natural environment.

These processes are truly creative in their nature, as they transform the human life environment into a valuable system of values, called an axiological system. These processes do not present a simple or easy reality for psychological research, or for the experimental approach in particular (Cushman et al., 2006).

Grác (2015) states that moral reasoning implies two sets of mental processes related to moral norms. The first set is related to the mental interiorization of moral norms, and the second set deals with the regulative function of moral norms in an individual's personal life, social interactions, and the natural environment. These mental processes create real-life moral situations, where moral norms interact with executive and legislative norms. Grác (2008, 2015) presents the internormative extensional relations between executive, moral, and legislative norms using Venn diagrams (Venn, 1884), what can be seen in Figure 1. As a justification of the purposefulness of using Venn diagrams in our analysis we can state that there is the need to prevent inaccuracies resulting from the extensional crossover of the norms under consideration. Such inaccuracies are indicated by Sudić & Ćirić, (2019) who pointed out that they are unsure to what degree conventional and abstract rules contaminated moral rules in their experiment.

Space for Figure 1

Figure 1. The internormative extensional relations between executive, moral and legislative norms.

MATERIALS AND METHODS

The Venn diagram illustrated in Figure 1 demonstrates that if the logical point of view is taken into consideration, we can see the relations between the three universal classes of norms: executive (E), moral (M), and legislative (L); thus, we can dislocate the extensional connections into the seven separate subclasses of norms. Both the executive and legal norms are described in Grác's (2015) classification as beyond moral ones.

What characterizes executive norms? According to Grác (2008) they are unambiguous regulators of the behavior manifested in specific situations, e.g., in contact with nature, with other people, with oneself. They are oriented towards the *hic et nunc* point of view, which is the optimum performance of certain activities. For example, in a sport, a sports record can be quickly replaced by another sports record (ski jumping hill record is marked in green to be visible to the jumpers). Another clear feature of this type of normative regulations is that although they improve human activities and accompany various mental experiences (e.g., attitudes, motives), the content of these experiences themselves is not subject to assessment by these norms. Thus, it can be said that neither the views nor the feelings accompanying the performance of these activities are subject to regulation within the framework of moral norms.

What do legal norms refer to? Grác (2008) indicates that the subject of their regulation is also the psychological dimension of the behavior manifested, which is characterized by strictly defined effects. The specificity of these terms is that from the point of view of an injunction and prohibition, they strictly and unequivocally demarcate not only the lower limit of the effect (which is still allowed), but also the upper limit of this

Formatted: Font: Italic

manifestation (which is already prohibited). The dimension of mental experiences accompanying the behavior, on the basis of a subjective cause, makes it possible to explain the objective effect, which facilitates the legal classification of the committed act as a crime or crime, which allows for the tightening or leniency of the sanction provided for in criminal law.

If we denote the general class of these norm as CN, and the symbol ∩ denotes logical conjunction, the mutual relations presented in Figure 1 can be expressed in Formula (1), respectively:

$$CN = \{E, M, L, (E \cap M), (L \cap M), (E \cap L)\ (E \cap M \cap L)\} \quad (1)$$

As moral norms will be the main point of reference in our experimental approach, we will now pay more attention to the subclasses from Formula (1) which contain only such norms. We will define these kinds of moral norms as pure or context-free. The first such subclass, symbolized as $\dot{M}$, denotes the moral norms which belong only to the universe of moral norms but which do not belong to executive or legislative norms. As such, and where *x* symbolizes any moral norm, and *ε* is a predicator denoting *belonging to*, this subclass may be strictly defined as in Formula (2):

$$\dot{M} = \{x: x \in M \cap \{x \notin E \cap x \notin L\}\} \quad (2)$$

The second two subclasses are the conjunctions of moral norms with legislative norms: M∩L and moral norms with executive norms: E∩M. The appropriate formal explications can be viewed in Formulas (3) and (4), respectively:

$$M \cap L = \{x: x \in M \cap L\} \quad (3)$$

$$M \cap E = \{x: x \in M \cap E\} \quad (4)$$

There is also a subclass of moral norms in Formula (1) which is a conjunction of the three norms, executive, moral, and legislative: $E \cap M \cap L$, which is expressed in Formula (5) as:

$$E \cap M \cap L = \{x : x \in E \cap M \cap L\} \quad (5)$$

The moral norms presented above will be employed in our experiment in the context of moral situation referred to as *ethical stories* or briefly, as *moralities.* This method has already been employed in psychological research on moral issues, e.g., by Piaget (1976) and Kohlberg (1976) to diagnose the moral development of children and youth.

It is worth reviewing the strong and weak aspects of using *ethical stories* in psychological research. We propose conducting a short SWOT analysis of *moralities* in empirical research to confront this method with personal experience or observations of real-life moral situations. The results of this analysis are collected in Table 1.

Table 1. The outcome of SWOT comparative analysis outcomes of direct observation of real-life moral situation and ethical stories (moralities).

Methods / Evaluation	Personal experience or direct observation of a moral situation	Ethical stories or moralities
Strong sides of the method	1. Acknowledges the rich context of a moral situation.<br>2. Enables employing an idiographic analysis of ethical evaluations of human behavior.	1. Enables grasping *hic et nunc* the ethical essence of the moral situation.<br>2. Enables employing nomothetic oriented research in ethical

		evaluation of human behavior.
Weak sides of the method	In many moral situations the moral motives and intentions are hidden to the external observer.	Abstracts from the entire context of the moral situation.

From the outcomes of SWOT analysis, we learn that ethical stories are more easily employed in nomothetic empirical analysis on moral situations. It must be stressed clearly, however, that the subject of our research will be the cognitive aspect of ethical evaluation related to the concept of *morally good performance in moralities* analysis. Cognitive schemas of ethical stories offer the keys to understanding the concept of internalized behavior.

Cognitive schemas of ethical stories

Let us review the main concepts related to ethical stories. Indicators of moral context relate to analysis of cognitive schemas of ethical stories. One indicator is a *moral requirement* or *demand* to do something in a defined situation, which is usually expressed by employing the functor of assertive judgement *should* that was systematically elaborated by Immanuel Kant as one of his three formulations of a *categorical imperative (kategorischer Imperativ),* which is expressed as: "Act only in accordance with that maxim through which you can at the same time will that it become a universal law" (Kant, 1966, 4:421).[1] Without this indicator, none of the moral norms defined in Formulas (1)-(5) could exist at all. If we state that the symbols *CI* denote Categorical Imperative, we can conclude that the norm regulating one's behavior is moral. This moral condition can be expressed as an alternative

[1] According with the Kantian works, the above quoted citation is: (G 4:421), i.e. E. Kant (1966). Groundwork of the Metaphysics of Morals, in I. Kant, Practical Philosophy, translated by Mary Gregor, Cambridge; Cambridge University Press, Chapter 4, page 421.

connection of the particular subsets of moral norms defined in the appropriate Formulas (2)-(5):

$$x \in CI \Rightarrow \{[(x \in M) \cap (x \notin E) \cap (x \notin L)] U [(x \in M \cap L)] U [(x \in M \cap E)] U [(x \in E \cap M \cap L)]\} \quad (6)$$

Another indicator of morality is here the object of moral regulation, which is understood as a content or domain regulated by the particular moral norms indicated in (6). Morally good performance assumes a specific object within which a particular moral norm has been actualized. Good examples could be the ethical codes of various professions. It is precisely stated in such codes that particular content-oriented professional activities are extracted from the remaining professional activities to be regulated by moral norms.

Also belonging to the indicators of morality are what causes a moral norm to become the given norm of the content of a the concrete ethical story, that is, what specifically distinguishes the given norm (specified in Formula (6)) from any other outside normative moral activity of a human being (as regulated by legislative – L, or executive – E norm, as seen in Figure 1 and in Formula (1)).

~~Such particular indicators could be, e.g. *intention* (motivation, plane, thought, idea, design), *concordance* (acceptation, assent, agreement), *application* (adoption, appliance, adhibition), *outcome* (result, consequence, effect, coherence, consistency), *knowledge* (cognition, learning, recognition, study), realization (implementation, accomplishment, fruition, completion), etc.~~

~~In our analysis we will denote the particular indicators of morality by the first letters of their names, as follows: intention – I; concordance – C; application – A; outcome – O; knowledge – K; realization – R. The above indicators, as we shall later see, have unequal regulative strength, which means that in a given ethical story one indicator could be exchanged by another one or, in some cases, might not even be taken into consideration at~~

all. Grác (2008) gives the examples of indicators of weaker regulative status could be: Knowledge (K) or Outcome (O).

After reviewing the literature on indicators of morality (Moore, 2005; Nikolaičeva, 1976; Piaget, 1976; Kohlberg, 1976), Grác (2008, 2015) decided to apply the following six indicators for the experimental design: intention – I; concordance – C; application – A; outcome – E; knowledge – K; and realization – R. Realizing that moral reasoning related to ethical evaluation of human behavior in real-life situations is definitely a complicated mental process, we have decided to restructure the above indicators into complementary experimental units, in the two cognitive schemes expressed formally as:

$$IAO = I \cap A \cap O \qquad (7)$$

$$KCR = K \cap C \cap R \qquad (8)$$

The issue is: what values may the indicators in the Formulas (7) and (8) take? It is not a problem to apply the mathematical symbols (+) and (-) to denote, by analogy, an evaluation of human behavior in the ethical story as morally good or bad, respectively. However, these two bipolar values are not sufficient for moral evaluation of ethical stories, because in these stories we can meet with human behavior which, for various reasons, is beyond moral evaluation. That is why we have introduced a third neutral value that may be assigned to each indicator employed in our experiment. The symbol Ø taken from Boolean algebra is applied here. This value will denote that in terms of the specific indicator, the analyzed human performance is outside or beyond the specific aspect of moral evaluation. Moreover, it has to be emphasized here that in the cognitive schemas constructed in this experiment, the denotation of each indicator of morality is precisely defined not only by marking it as (+), (-) or Ø, but also by giving a particular interpretation to it in respect to the specific moral dimensions in these schemas. These symbols will be called antecedent marks

in our analysis. We term these specific interpretations as *definiens*, which will be precisely defined for the particular schemas (see Table 2 and Table 3). Next, we present the cognitive schemas of IAO and KCR as the matrices for this experiment on moral reasoning.

IAO cognitive schemas

Indicators for the moral assessment and their definiens, i.e., criteria for their evaluation are defined in Table 2.

Table 2. The indicators of morality aspects and their definiens, i.e., criteria for their ethical assessment in the IAO schemas.

Indicators of morality aspects	Criteria for their ethical assessment		
**I**NTENTION– the behavior intention, incentive and the moral norm are:	(+) in accord	(-) in discord	(∅) neither
**A**PPLICATION – the form applied in this particular case in relation to the moral norm:	(+) corresponds	(-) does not correspond	(∅) neither
**O**UTCOME **–** consequence accompanying, possible outcome is:	(+) positive	(-) negative	(∅) neither

These indicators are specific conditions under which any moral norm works in a certain life situation. The criteria are definientia (sing. *definiens*) of the moral norm effect,

expressing the fact that each of the perpendicularly identical symbols (+ - Ø) has a different notional content – depending on its combination with the indicators.

The first indicator of the IAO schema is Intention – I. It is understood as a motive or intent of the individual's ethical behavior. As an individual cannot be forced to be moral, the axiological principle in ethics assumes his or her deliberation and free decision-making in the moral norm interactions. For this reason, this indicator is regarded as exceptionally important. But the indicator of the person's motive or intention cannot be absolutized since the individual's conscious relationship to a moral norm is an inevitable but not sufficient precondition for an ethics-based assessment of the person's behavior. How the concrete behavior will be evaluated from the perspective of intention and motivation depends on the criterion definientia belonging to this indicator. If, for example, the motive or intention of the behavioral event is in accord with the moral norm, then the result of the partial evaluation of behavior is positive (+). If it is in discord, the result is negative (-). The symbol Ø (*nullus*) signals that neither of the previous assessments are valid. Usually, this refers to activities which, due to their content, are regarded as beyond moral, but also to those where the motive of behavior is regulated by a moral norm, but the person is not aware of it.

Application – A, the second indicator of the IAR scheme, answers the question of whether the intention which is moral is or is not obtained by moral means. If the means of the intent's actualization corresponds with the moral norm, the result is positive (+). If the person tried to achieve the intended good by unethical means, their behavior is evaluated negatively (-). This means that the application indicator always assumes re-activation of the original intent and thus there is also the possibility of a greater or smaller change in the application process; consequently, this resultant motive may, but also may not, be in accordance with the original motive. The fact that every application implies not only

motivation but also its actualization gives this indicator, according to the IAO scheme, a crucial position in the evaluation of the individual's ethical behavior.

The third indicator of the IAO scheme is Outcome – O, as a possible or an accompanying outcome. The indicator in question is related to the assessed behavioral event. The concept of relation itself can be understood as causal or non-causal. It could be causal in the case where the intention indicator was the cause of the actualized intention and the consequence indicator was the impact of it. Since the function of the actualized intention was already adopted in the IAO scheme by the intentional indicator (as mentioned above), it is not possible – in the framework of the same scheme – to assign it simultaneously to the third indicator. Therefore, the Outcome indicator can be understood in the given scheme exclusively as non-causal, i.e. as a consequential circumstance which is related to cause and effect, but which cannot be identified with it. From the above-mentioned analysis, it follows that the assessment of the person's behavior by means of three signs (+ - Ø) applies only to the evaluation of the accompanying consequence *per se* and does not influence the overall evaluation of the individual's behavior in the given moral situation.

For the above reasons, the ethical evaluation processes in a moral situation within the cognitive scheme of IAO could be formulated as follows (9):

$$\prod_{I,A,O} \sum_{\{x:x\in\{(+),(-),\emptyset\}\}} = \{[I=(+) \cup I=(-) \cup I=\emptyset] \cap [A=(+) \cup A(-) \cup A=\emptyset)] \cap [O=(+) \cup O=(-) \cup O=\emptyset]\}$$

KCR cognitive schemas

Indicators of moral assessment related to the cognitive schemas KCR and the criteria for their evaluation are presented (so called *definientia*) in Table 3.

Table 3. The indicators of morality aspects and their definiens, i.e. criteria for their ethical assessment in KCR schemas.

Indicators of morality aspects	Criteria for their ethical assessment		
**K**nowledge – as a cognitive component to the specific moral norm	(+) exists	(-) doesn't exist	(∅) neither
**C**oncordance – acceptance of the particular moral norm	(+) is accepted	(-) is not accepted	(∅) neither
**R**ealizing **–** the moral norm has actually been implemented	(+) fulfilled	(-) not fulfilled	(∅) neither

The first indicator of morality in the KCR schema is Knowledge about moral norms, which regulates human behavior in a given situation. This is really moral knowledge about the specific norm, which can exist as a socially accepted norm within public opinion, regulating interpersonal relations. However, the acting person may or may not have knowledge about this opinion. Thus, the cognition of the moral norm can be very differentiated. The norm can exist as a socially accepted ethical obligation which a concrete individual might not know about. There could also be cases where an individual has a positive attitude toward the defined requirement and accepts it as his or her own moral norm, independently of whether such norm objectively exists as a social standard or not. The symbol Ø can be used to value this indicator, if is really unclear as to whether to assign a value (+) or (-).

The second indicator in KCR is Concordance (C) of the person interacting in a given moral situation. Concordance means the actor's personal attitude to the particular moral

norm regulating human performance in this situation. According to Grác (2008, 2015), using the signs: (+) or (-) in valuing the indicator C is relatively univocal, but some problems concerning understanding may require the sign Ø. This sign should be employed if the evaluating person has neither a positive nor negative attitude towards the given moral norm or when this person does not have his or her own understanding of the moral requirements related to the norm in question.

The last indicator Realizing – R refers to whether and how the requirements of the moral norm regulating human behavior are fulfilled. A positive (+) value of this indicator signifies that implementation of the moral norm is fulfilled. This indicator receives a negative value if implementation of the moral norm is not fulfilled in a situation where it is supposed to be. Ethical evaluation faces greater difficulty when considering whether to assign the value Ø. This case is particularly difficult to assign due to the fact that there is no analogy between a neural attitude towards a moral norm (as in the case of indicator C above) and the neutrality of acting in a concrete situation, when we are dealing with human performance.

In concluding this stage of our analysis, we can state that ethical evaluation processes in a moral-content-situation within the cognitive scheme of KCR can be formulated as follows (10):

$$\prod_{K,R,C} \sum_{\{x:x\in\{(+),(-),\emptyset\}\}} = \{[K=(+) \cup K=(-) \cup K=\emptyset] \cap [R=(+) \cup R(-) \cup R=\emptyset)] \cap [C=(+) \cup C=(-) \cup C=\emptyset]\}$$

Control of independent variables in evaluating cognitive schemas of ethical story experiments

The main methodological requirement for any experimental condition is to control the independent variables which make an impact on the dependent variable. In our experiment on moral thinking, the main dependent variable to be explained is the ethical evaluation of human behavior in terms of moral values. It is assumed that moral reasoning is a structured mental process like any other human thinking structured towards problem solving. Since John Dewey's *How we think* (Dewey, 1910), psychologists working on problem solving have distinguished the phases of thinking which lead human thinking to reach the goal of solving a problem. Similar thinking takes place in a real-life moral situation where a human being considers whether and which action should be taken in order to behave in a morally acceptable way, in accordance with his or her conscience – based on a moral norm.

Our experiment will be based on the evaluation of ethical stories (moralities), using the defined structured indicators of morality, called cognitive schemas of the ethical-dilemma-stories, described above as IAO and KCR.

The first novelty of this experimental approach is the two-phase design: (1) modeling of ethic-dilemma stories based on logically structured cognitive schemas, and (2) overall ethical evaluation of the modeled morality schemas.

Assessing the effect of any moral norm in conditions of ~~natural~~ an experiment situation, given by the schemas IAO and KCR, is one methodological challenge; another is grasping these conditions in a person's actual behavior. This can be done by means of modeling written stories which present real-life moral situations. These stories enable the observer to examine intrinsically experienced behavior, e.g., their motivation or intention, which is usually hidden from a second party's retrospection.

We use the term *modeling* for the creation of moral dilemma stories. This involves the assignment of arbitrary content of actually displayed behavior to the respective value

indicators of the schema, so that this allocation may accurately correspond to the criterion definientia of the indicators (I,A,O,K,C,R) of morality. Educational psychology students were the subjects of this ~~quasi-natural~~ experiment. They were asked to create model ethical stories concerning moral situations of young students of their age, according to carefully-prepared instructions informing them how to do this task.

Control of independent variables in the experiment

Three question arose in the definition of *modeling*. The first of these is formulated as follows: how many *independent variables*, that is, different configurations of signs, are offered for story modeling by the schemas IAO and KCR, respectively?

In order to answer this question for IAO schemas we have to strictly consider the three indicators of morality where each of them, formally speaking, may take three possible values: (+), (-), Ø. Thus, to calculate all configurations of each indicator combinatorically, where generally its number is *n,* where the number of values that each of the indicators can take may be generally symbolized by *r*, we could apply the formula for variations with repetition n^r. This means that variations with *n* elements of the rth class are all possible configurations of *n* indicators of morality and the *r* values which each indicator can take. In our case, where the number of indicators *n*=3 in the IAO schemas, and the number of values which each of them can take is r=3, the number of all configurations is simply attained using Formula (11) as follows:

$$\{N(IAO): N(IM) = 3 \ \cap N(VIM) = 3\} \Rightarrow \{N(IAO) = 3^3 = 27\} \qquad (11)$$

where:

N(IAO) – the total number of independent variable configurations related to the indicators of morality and their values in the IAO schemas;

N(IM) – the number of indicators of morality in the IAO schemas;

N(VIM) – the number of values that can be taken by each of the indicators of morality in the IAO schemas.

And, similarly, for the KCR schemas, where the number of indicators is also n=3, and the number of values which each of them can take is r=3 as well, we can reach the number of all configurations simply by using Formula (12) being a replication of Formula (11) as follows:

$$\{N(KCR): N(IM) = 3 \cap N(VIM) = 3\} \Rightarrow \{N(KCR) = 3^3 = 27\} \qquad (12)$$

where:

N(KCR) – the total number of independent variable configurations related with the indicators of morality and their values in the KCR schemas;

N(IM) and N(VIM) – are as in Formula (11).

The elementary experimental fields of moral evaluation: ethical stories

The second question is whether the same story content, which is modeled according to certain specific criteria of evaluation, can be *modified* in accord with further randomly applied evaluation criteria of the IAO or KCR schemas. In an effort to answer this question, we can alternately replace each indicator of morality by one of the criterion values: (+), (-), Ø. If we take as an example the description of three 18-years-old youths who solved the problem of moving to a new place to live in different ways, we obtain the IAO and KCR schema matrices with nine boxes as a part of 27 total number of combinatorial boxes, accounted separately for the IAO and KCR cognitive schemas, as presented in Formulas (11) and (12), respectively. For the schema matrixed in our experiment the total number of rows i=3, and the number of columns is c=3. If any line is denoted as i and any column as j, then

any field in these matrices can be expressed as an experimental field EF_{ij} of partial moral evaluation of human performance in ethical story. An example of such EF_{ij} are shown in Table 4 for the IAO schemas.

Table 4. Matrix of IAO schemas illustrating the elementary experimental fields of moral evaluation in ethical stories.

columns / rows	*c*=1	*c*=2	*c*=3
i=1	**I (+)** John found a new flat to create better conditions for his siblings to study.	**A (-)** He did it regardless of the disapproval of his mother, who felt almost mortally hurt by his decision.	**O (∅)** After having moved to his new flat John found out that the signal for his cell phone was the same quality as in the previous location.
i=2	**I (-)** Paul found a new flat to have easier access to drug dealers.	**A (∅)** While moving to the new flat, he decided to travel by train instead by bus.	**O (+)** At the new location he met a neighbor who began to exert a positive influence on him.
	**I (∅)** Peter found a new flat	**A (+)** By mutual consent with his	**O (-)** While moving to the new

i=3	because he wanted to live alone.	parents he left their home.	flat he slipped and broke his leg.

As in our case, the IAO scheme is a square matrix, the values in the first line can be written symbolically: i_1 c_1 = I (+), i_1 c_2 = A (-), i_1 c_3 = O (∅) and at the same time they can be checked by reading them verbally in the first row of the chart. The square matrix enables us to read the same story plot variably, i.e., according to an arbitrary configuration of boxes in the lines, without losing the inner logic (meaning). For example, if we select only positive criteria, then the story plot can be expressed in the following notation: i_1 c_1 = I (+), i_3 c_2 = A (+), i_2 c_3 = O (+). When we read it in the chart in this transcript, we find out that the story plot did not lose its inner coherence. It is possible to symbolically record only the minus or only the null configuration in the same way. However, any random two-element combinations are also possible, e.g., combination of two negative and one positive value. In that case, the notation is as follows: i_2 c_1 = I (-) , i_1 c_2 = A (-), i_2 c_3 = O (+).

Based on the above, the IAO schemas make it possible not only to create stories differing in their content, but they also offer the opportunity to modify the already-modeled stories.

In a similar way, we will now give an example of experimental fields EF_{ij} of the schemas KCR shown in the square matrix presented in Table 5.

Table 5. Matrix of the KCR schemas illustrating the elementary experimental fields (EF_{ij}) of moral evaluation in ethical stories.

columns	*c*=1	*c*=2	*c*=3

lines			
i=1	**K (+)** Igor knew that cruelty to animals is immoral.	**C (-)** He was not opposed to ill-treatment of animals.	**R (∅)** However, he has never even been in a situation where he watched cruelty inflicted on animals by others.
i=2	**K (-)** Andrew did not know that cruelty to animals is immoral.	**C (∅)** In view of the abuse of animals, he was unclear and of a contradictory position.	**R (+)** Yesterday, when his colleagues mistreated a dog, he protected it.
i=3	**K (∅)** Emil was not sure if cruelty to animals is moral or immoral.	**C (+)** But personally he did not agree with the abuse of animals.	**R (-)** However, yesterday, when colleagues mistreated a dog, he was also cruel to it.

From Table 5 we can see that KCR schemas, similar to IAO schemas, also have a variable structure of stimuli constituted by the content of combinatorial sequences of elementary experimental fields (EF_{ij}) at a level equal to IAO. These experimental fields are indicators of morality of each individual ethical story presented in both the IAO and KCR schemas. The experimental control of independent variables in this experiment deals with

combinatorial control of the particular scheme in terms of the formal values (+), (-) and ∅ in IAO and KCR, respectively. Moreover, in order to avoid contradictions between the concrete schemas, on the one hand, and to maintain individual and personal coherence of the ethical story content, on the other, the subjects were the heroes of each of the 27 IAO schemas and 27 KCR schemas, where each schema actor was assigned a different name in the modeled ethical stories.

Experimental design

In our experiment the stimuli are the individual schemas, which consist of three indicators of morality ordered in the two following sequences: IAO and KCR, where each of the indicators takes only one of three values in its matrix of sequences (see Table 4 and Table 5). The valuated indicators of morality constitute a concrete cognitive schema as a complex sequential stimulus which consists of three particular experimental fields as the objects of ethical individual story modeling in the first phase of the experiment.

Thus, each individual IAO schema can be defined as a well-ordered set of three:

$$Si(\mathrm{IAO}) \stackrel{\mathrm{def}}{=} \langle I, A, O \rangle \qquad (13),$$

where: S_i(IAO) - any of the *n* (*n*=27 in our experiment) IAO schemas; I={(+),(-),∅}**;** A={(+),(-),∅}; O={(+),(-),∅} **–** denote the sets of values which can take particular indicators of morality in the IAO schemas. This indicates that the order of the indicators constituting the IAO schema should not be changed.

In a similar way, each individual KCR schema can be defined as a well-ordered set of three:

$$Si(KCR) \stackrel{\mathrm{def}}{=} \langle K, C, R \rangle \qquad (14),$$

where: S_i(KCR) - any of n (n=27 in our experiment) KCR schemas; K{(+),(-),∅}; C={(+),(-),∅}; R={(+),(-),∅} – denote the sets of values which can take the particular indicators of morality of the KCR schemas.

The methodological analysis conducted allows us to formulate an experimental design to present cognitive schemas for ethical story modeling by educational psychology students. These ethical stories involve moral issues from the students' own community. This design is included in Table 6 as a matrix of formal schemas numbered from 1 to 54 and presented in parallel columns in such a way that numbers for schemas IAO and KCR are located on either side of the formal valuation structures of the indicators of morality.

Table 6. The experimental design: the structure of valuation of indicators in particular IAO and KCR cognitive schemas for experimental modeling of ethical stories by educational psychology students.

IAO schemas	Modeling formal patterns	KCR schemas	IAO schemas	Modeling formal patterns	KCR schemas	IAO schemas	Modeling formal patterns	KCR schemas
1	(+ + +)	28	10	(∅ - **-)**	37	19	(- ∅ **+)**	46
2	(+ - +)	29	11	(- - ∅**)**	38	20	(+ - ∅**)**	47
3	(- + +)	30	12	**(**∅∅∅**)**	39	21	(+ ∅ **-)**	48
4	(+ + -)	31	13	(- - +)	40	22	**(**∅ **-** ∅**)**	49
5	(+ ∅ **+)**	32	14	(- + -)	41	23	**(**∅∅ **-)**	50
6	**(**∅ **+ +)**	33	15	(+ - -)	42	24	(- ∅∅**)**	51

7	(+ + ∅)	34	16	(∅ + -)	43	25	(∅∅ +)	52
8	(- - -)	35	17	(+ - ∅)	44	26	(∅ + ∅)	53
9	(- ∅ -)	36	18	(∅ - +)	45	27	(+ ∅∅)	54

The order number of formal valuations of the IAO schema is always on the left side of the appropriate column for modeling formal patterns in Table 6 and the order number for the KCR schema is on the right side of this column, respectively. The signs of moral evaluation should be read from left to right both for IAO and KCR schemas.

From Table 6 we learn that both cognitive schema enable the planned experiment on moral thinking to control for variability of stimuli structure via evaluated indicators of morality of human performance modeled in ~~quasi-natural~~ experimental conditions during the students' classes.

Subjects, experiment – (a) modeling instructions and procedure, and (b) overall moral evaluation of ethical stories

Subject Group 1

Subject Group 1 were 134 third-year educational psychology students of a modal age of 20 years, who participated in both parts of our experiment, i.e., in the modeling of ethical stories, and in the overall evaluating of these stories modeled by themselves. Their participation in this experiment came as part of a regular course on educational psychology at the Faculty of Philosophy of Trnava University in Trnava (Slovakia) over the years 1998-2000. 80% of the subjects were female students and 20% were males. After participating in theoretical lectures on cognitive schemas of ethical stories and evaluation of related indicators of morality, the students of the seminar on educational psychology were presented with the following task in accordance with these short instructions:

Experiment instructions and procedure for Subject Group 1

As you have learned today from the lecture on education psychology, each schema of an ethical story has its own order number in our experimental matrix and a concrete valuation of its three indicators of morality. Now you will receive data about two IAO schemas and two KCR schemas. Your task will be to creatively model the thinking and performance of a 17-year-old individual, different in each of the projected stories, in accordance with the given values of the individual indicators of morality according to the

patterns of potential schemas, the order numbers of which will be generated by random number generator.

After this instruction each individual subject was given the *Ethical Story Modeling Experiment Report Form* (see the form with this protocol in the Appendix) containing four order numbers associated with indicator values for potential ethical stories generated from Table 6 by a random number generator. The subject was instructed about where and what the modeling schema of the concrete story should be in accordance to the experimental design, and that the hero of each story should be an individual person known by his or her name.

Once the modeling phase of the experiment was completed, in the next phase the experimenter asked the same subject to conduct an overall evaluation of each of the constructed ethical stories in his or her protocol as a whole by assigning a global symbol in square brackets [ ] to denote their overall assessment of moral values of human performance as: morally good [+], morally bad [-] or morally neutral [∅] which are now called the concluding symbols.

The experimental modeling procedure and overall moral evaluation of ethical stories took place in a seminar room where the subjects could model the educational stories in quiet and comfortable conditions. They conducted their experimental modeling and overall moral evaluation individually, sitting separately at seminar tables in small groups. The average time for modeling was about 45 minutes. After completing the story modeling and conducting the overall evaluation of each of the constructed ethical stories, the students gave the completed *Protocol for Experimental Modeling of Ethical Stories* and their completed *Moral Evaluation* back to the experimenter (see: APPENDIX 1).

Subject Group 2

Taking into account that the studied group of students (Subject Group 1) participated in the entire experiment, i.e. in modeling and then in evaluating the entire ethical story, we can state that they had a fairly high level of ethical knowledge, particularly on overall evaluation of ethical stories, gained mainly from lectures on the psychology of morality. This, however, completely rules out the possibility of using the research results from the second part of the experiment as representative for the overall evaluation of the ethical stories constructed in our experiment by the young students.

Therefore, the authors of this study decided to select two other group of young people applying to study psychology for the second part of the experiment, i.e. for overall evaluation of the ethical stories (modeled in the first part of the experiment) as the subjects who are just after their maturation and participate in the entrance exams for psychology at the University of Trnava. These subjects are called Subject Group 2. Their number is 450 (N=450).

RESULTS

The research findings from this moral reasoning experiment are presented according to the two phases of this experiment: (1) the results of modeling ethical stories, and (2) the results of the overall moral evaluation of the modeled ethical stories (see: the example of the *Protocol of Experimental Modeling of Ethical Stories* and its completed *Moral Evaluation* back to the experimenter (see: APPENDIX 2).

The modeling phase

The total number of protocols received with completed modeling was 536. This means that the main result of our findings from the modeling phase of our experiment on moral reasoning is a systematized empirical data base of ethical stories, which is a kind of

empirical description of characteristics of denotative meaning of the indicators of morality understood in their positive, negative, or neutral meaning in a context of cognitive schemas of ethical stories. This base is systematized in accordance with the order numbers of the 54 IAO and KCR cognitive schemas, sequenced and presented as a matrix in Table 6. One way of presenting this data base to list the modal examples of modeling created on the base of this matrix. The full list of modal models can be found in the original Slovakian version of the report by Grác (2008, pp 119-125) and the Polish version by Grác (2015, pp.140-148).

This data base can be a source for qualitative analysis on the structuring of moral reasoning using indicators of morality. One of the opportunities for research using this data base points is to use the schemas for research on overall assessment of the moral value of the ethical stories. This is what was done in the second phase of this research project.

The moral evaluation phase

We will now show what kind of cognitive strategies the subjects developed in their overall moral evaluations, based on analyzing the values of indicators of morality as premises in inferring the conclusions in the IAO and then KCR schemas.

Strategies used to complete overall moral evaluations of the IAO schemas of ethical stories

~~The first main finding concerning the strategies used by students to conduct overall moral evaluations of the IAO schemas of ethical stories is that the indicator of outcomes (O) of ethical stories did not have any impact on the overall evaluation of these stories. This means that for the subjects of the study, this indicator did not determine their moral evaluation of ethical stories, but only offered a situational context for these stories.~~

The students' overall moral evaluations of the IAO ethical stories allowed us to identify three regularities which describe their moral reasoning in analyzing the values of the indicators of morality in these stories. These regularities are also intuitively deducible from the syntactic structure of the indicators of morality contained in the structures of formal ethical story schemas used in our experiment. These syntactic regularities are referred to as the model strategies of overall evaluations of ethical stories.

The moral strategies of overall evaluations of IAO ethical stories

The first regularity can be called the *principle of univocal values of intention and application indicators*. The matrix which indicates the overall moral evaluations which fit this regularity is shown in Table 7.

Table 7. Global moral evaluations of the IAO schemas in accordance with the principle of univocal values of intention and application indicators

Order number of the schema	IAO schema	Overall moral evaluation	Order number of the schema	IAO schema	Overall moral evaluation
1	(+ + +)	[+]	12	(∅∅∅)	[∅]
4	(+ + -)	[+]	13	(- - +)	[-]
7	(+ + ∅)	[+]	23	(∅∅ -)	[∅]
8	(- - -)	[-]	25	(∅∅ +)	[∅]
11	(- - ∅)	[-]			

The analysis of Table 7 allows us to formulate *a principle of univocal values of intention and application indicators* as follows:

$$(I = A) \Rightarrow (ES = I = A) \quad (15),$$

where: ES denotes the overall moral evaluation of ethical story; I and A denote the appropriate signs of moral values of the indicators in IAO schemas.

The second regularity of the overall evaluation of the IAO schemas of ethical stories is called the *principle of preferring application (A) indicator over intention indicator (I)*. Table 8 shows the matrix indicating the overall moral evaluations which fit this principle.

Table 8. Overall moral evaluations of IAO schemas in accordance with the principle of preferring application (A) indicator over intention indicator (I).

Order number of the schema	IAO schema	Overall moral evaluation	Order number of the schema	IAO schema	Overall moral evaluation
2	(+ - +)	[-]	16	(∅ + -)	[+]
3	(- + +)	[+]	17	(- + ∅)	[+]
6	(∅ + +)	[+]	18	(∅ - +)	[-]
10	(∅ - -)	[-]	20	(+ - ∅)	[-]
14	(- + -)	[+]	22	(∅ - ∅)	[-]
15	(+ - -)	[-]	26	(∅ + ∅)	[+]

From Table 8 we can learn that *the principle of preferring application (A) indicator over intention indicator (I)* may be formally expressed as:

$$(I \neq A) \Rightarrow (ES = A) \quad (16),$$

where the symbols denote the same as in (15).

Better to try and fail than to never have tried?

The third regularity says that if the intention indicator is non-zero, and the sign of the application indicator is zero, the concluded overall moral evaluation of the ethical story is formally expressed by the sign of the intention indicator, independently of the sign of the outcome indicator. This regularity is called as the *principle of preferring the non-zero intention indicator (I) over the zero application indicator (A)*. The designates of this principle are shown in the matrix presented in Table 9.

Table 9. Overall moral evaluations of IAO schemas in accordance with the principle of preferring the non-zero intention indicator (I) over zero application (A) indicator.

Order number of the schema	IAO schema	Concluded moral evaluation	Order number of the schema	IAO schema	Concluded moral evaluation
5	(+∅ +)	[+]	21	(+∅ **-**)	[+]
9	(-∅ **-**)	[-]	24	(-∅∅**)**	[-]
19	(-∅ **+)**	[-]	27	(+∅∅**)**	[+]

Analysis of the patterns of indicator signs allows us to formulate in a formal way the principle of preferring the non-zero intention indicator (I) over the zero application indicator A expressed as:

$$[(I \neq \varnothing) \cap (\mathbf{A} = \varnothing)] \Rightarrow (ES = I) \qquad (17).$$

Representativeness of the syntactic models in overall evaluations of IAO ethical stories

At what level are, the above-presented models of overall evaluations of IAO ethical stories, descriptive for the subjects? Overall evaluations of IAO ethical stories extracted from the empirical data base were carried out, having been obtained in the modeling part of the experiment and prepared in the ZEU-IAO Test for further research. The participants of this part of the experiment (N=450) were high school graduates who took their entrance exams for psychology at the University of Trnava (Slovakia). In 93.75% of the IAO ethical stories schemas, the modal evaluation of the subjects turned out to be consistent with the model strategy of logic-formal syntax. The percentages of these strategies ranged from 28.70% to 98.00%.[2]

These results were consistent with a study carried out three years later with another group of high school graduates (N = 355) participating in the entrance exams for psychology at the same university. They were tested with the same ZEU-IAO test as the group of their colleagues earlier. Both study groups had no contact with each other in the interim. The second study showed that for 93.75% of the IAO ethical stories schemas, the modal evaluation of the subjects turned out to be consistent with the model strategy of logic-formal syntax indicated in Tables 7-9 and in Formulas (15)-(17), respectively. This means that the strategy of integrating the morality indicators of moral stories into IAO schemes resulted in highly descriptive models for the processes of moral evaluation of these stories. The percent of subjects employing these strategies in the experimental ethical stories ranged from 32.00% to 98.30%.[3]

Strategies used to complete overall moral evaluations of the KCR schemas of ethical stories

[2] See: Grác (2008) Chapter VI, pp.135-145, Table 15.
[3] See: Grác (2008) Chapter VII, pp.155-178, Table 21.

The first main finding concluding the moral evaluation of the ethical stories within the KCR schemas is that only concordance (C) and realization (R) indicators of morality played a decisive role in the overall moral evaluations of the stories. Familiarity with the heroes of the stories of moral norms (K) appeared only to play a contextual role in the ethical stories, with no impact on completing the moral evaluation process.

The students' assessment data allowed us to identify the following four strategies in concluding the moral evaluations of the KCR schemas of ethical stories:

1. If the sign of the concordance indicator (C) is univocal with the sign of the realization indicator (R), the sign of the concluded moral evaluation of the ethical story is equal to the signs of both indicators in the premises of this conclusion, which can be formally expressed as:

$$(C = R) \Rightarrow (ES = C = R) \qquad (18),$$

where: ES – denotes the same as in the above formulas, and C, R denote the symbols of the KCR schemas.

2. If the sign of the concordance indicator (C) contradicts the sign of realization indicator (R), the overall moral evaluation is expressed with the sign $\varnothing$**.** As a formal expression it is stated as:

$$(I \neq R) \Rightarrow (ES = \varnothing) \qquad (19)$$

3. If the sign of the concordance indicator (C) is neutral ($\varnothing$),the overall concluded moral evaluation of the ethical story is neutral as well, which one can formulate as:

$$(C = \varnothing) \Rightarrow (ES = \varnothing) \qquad (20)$$

4. If the sign of the realization indicator (R) is neutral (∅),the overall moral evaluation of the ethical story takes its value from the non-zero evaluation of the concordance indicator (C). In a formal way this regularity may be expressed as:

$$(R = \varnothing) \Rightarrow (ES = C) \qquad (21)$$

Representativeness of the syntactic models in overall evaluations of KCR ethical stories

In this section we indicate to what extent the integration stretches of morality indicators in the overall evaluation of ethical stories according to the KCR scheme (formulated in Tables 10-13 and Formulas (18) - (21)) constitute descriptive models for students participating in the experimental evaluation of these stories. To do so, we use the test results of a group of high school graduates (N = 450) who participated simultaneously in the test (both the ZEU-IAO Test and the ZEU-KCR Test). Based on the general data for the degree of compliance between the empirical distribution of moral valuation of the respondents for the tested ethical stories and the indications of the models discussed above, it can be said that in 10 test tasks out of all 16 tasks displayed for the KCR scheme, model strategies turned out to be descriptive for the evaluation of the studied group of high school graduates. This means that in 62.50% of evaluation tasks, the modal number of respondents in 10 out of 16 groups of tasks, was consistent with the logical-formal syntax model. A more detailed presentation of this research situation is indicated in Table 22.[4]

Table 22. Percentage indicators of modal evaluations of moral stories KCR for the choices of respondents in relation to model indications

The order of	Values of	Overall	Modal	Modal percentages

[4] Tabela 22 została sporządzona w oparciu o dane z Rozdziału IX: Grác (2008, pp. 208-2012).

the ethical story in ZEU-KCR	morality indicators in the K C R	evaluation of the moral story	percentages of model evaluations*	of non-model evaluations
01	(+) (+) (+)	[+]	**79.4%**	
02	(+) (-) (-)	[-]	**49.2%**	
03	(-) (∅) (-)	[∅]	**64.0%**	
04	(+) (-) (+)	[∅]	36.4%	58.2% [-]
05	(-) (∅) (+)	[∅]	**50.9%**	
06	(-) (∅) (+)	[∅]	**70.2%**	
07	(+) (+) (+)	[+]	**89.0%**	
08	(+) (-) (-)	[-]	**89.2%**	
09	(-) (∅) (-)	[∅]	**68.3%**	
10	(+) (-) (+)	[∅]	27.0%	68.6% [-]
11	(+) (-) (-)	[-]	**70.2%**	
12	(+) (+) (-)	[∅]	15.8%	82.4% [-]
13	(+) (-) (+)	[∅]	44.7%	49.4% [-]
14	(+) (-) (-)	[-]	**86.1%**	
15	(+) (-) (+)	[∅]	36.5%	61.5% [+]
16	(+) (+) (-)	[∅]	19.8%	75.2% [-]

* The bolded percentages in the column fourth of Table 22 denote that these modal evaluations by the subjects fit in the defined ethical stories of the model strategies related to these stories.

The data from Table 22 allow us to more accurately conclude that the modal percentages of model evaluations are quite varied with respect to individual moral stories exposed in the experiment: from 49.2% to 89.2% of the respondents' modal moral evaluations.

~~DISCUSSION~~

CONCLUSION

In this section we will attempt to respond to the question of which mental processes are involved in both the modeling phase of our experiment and the overall evaluation of the ethical story. Grác (2008, 2015) assumes that in modeling ethical stories, semantic operations are involved to create thematic-oriented structures in accordance with the sequences determined by the formal signs of the indicators of morality which constitute the cognitive schemas of potential stories (moralities). We can only add that these semantic mental operations must be focused both on securing particular sequences for the appropriate moral valuation required by the cognitive schemas obtained in the experiment, and, at the same time, on maintaining the thematic coherence of the content of the whole ethical story.

We consider here the role in moral reasoning played by the particular indicators of morality known from the literature, such as motivation and intension (Moore, 2005; Chih-Feng et al., 2016). In the overall evaluation phase of the experiment, moral reasoning required more syntactic and formal logic cognitive operations. Grác distinguishes three processes that enabled the subjects to formulate the regularities in concluding the overall moral evaluation of ethical stories: *selection, differentiation* and *integration*.

The *selection process* deals with highlighting the indicators of morality that do not influence moral evaluation of ethical stories, but only create a situational context in these stories. These indicators appeared to be: the outcome indicator (O) in the IAO schemas, and

the knowledge indicator (K) in the KCR schemas. This means that these two indicators are not considered as premises in completing overall moral evaluation of ethical stories. The result of the *differentiation process* is in distinguishing among the indicators of morality which play a dominant role in competing the overall moral evaluation of moral stories, and those which are only connected with this evaluation. The dominant positions were taken by: the application indicator (A) in the IAO schemas and the concordance indicator (C) in the KCR schemas.

The main mental process related to conducting an overall moral evaluation is *integration*, which allows a generalizing value to be assigned for each group of three indicators in the IAO and KCR schemes. As a result of this process, seven regularities were identified that describe the generalized patterns of indicator sign in the premises and the inferred overall conclusion for each group of ethical stories. The relationship discovered between each of the particular groups of indicator signs as premises and the overall moral evaluations of the moralities constitutes a psychological connection for moral inference in our experiment on moral evaluations of ethical stories. Formally, this relationship can be expressed in the formal logic syllogism *modus ponendo ponens* as follows:

$$[(p \Rightarrow q) \cap p] \Rightarrow q \qquad (22),$$

where the symbols of inference connection ($p \Rightarrow q$) denote the particular regularities formulated in (17)-(21) as the first premise for conducting the moral evaluation, where *p* denotes the conditional sentence concerning the particular group of indicators signs of moral valuation and *q* denotes the result of the overall moral evaluation of ethical story.

The above discussion statement corresponds with A. Biela's (1986) commentaries on R.J. Sternberg's (1984) triarchic theory of human intelligence and his positive response to these comments (Sternberg, 1986). Starting from the statement that higher levels of moral

why introduce these observations here?

development are related to higher levels of cognitive development, Biela suggested that it is possible to include moral/axiological level to a theory of human intelligence without any decrease of its empirical testability (cf. Williams and Gantt, 2012). The presented study on moral reasoning evidently supports this suggestion.

Maybe this could be developed as part of the intro for the experiments?

Final Remarks

In conclusion, we have described the novelty of the two phase experiment conducted on moral reasoning, which included a moral story modeling phase and an overall moral evaluation phase developed by ~~the same subjects,~~ educational psychology students.

The moral story modeling phase developed by the students gave them the chance to learn what moral reasoning about youth behavior is in an attractive way, and what role the indicators of morality of their experimentally modeled ethical stories play in overall moral assessment of human performance. The modeling phase of the experiment enabled the subjects to take a large step towards the process of internalization of moral norms regulating human behavior in social situations. In addition, this process was of a multidimensional nature and a structured character, leading to the overall moral evaluation of human action.

In this experiment, students were able to create models of complex matter, using the indicators of moral evaluation of their own youth social performance. Thus, we may say that this experiment took place in closer proximity to real-life thinking processes than any other research method previously used in experimental psychology.

Financial Support / Funding Statement: This research received no specific grant from any funding agency, commercial or not-for-profit sectors.

Conflicts of Interest: None

References

Awad, E., Bonnefon, J., Shariff, A., & Rahwan, I. (2019). The Thorny Challenge of Making Moral Machines: Ethical Dilemmas with Self-Driving Cars, NIM Marketing Intelligence Review , 11(2), 42-47. doi: https://doi.org/10.2478/nimmir-2019-0015

Alfano, M. (2019). Moral reasoning is the process of asking moral questions and answering them. Behavioral and Brain Sciences, 42, E147. doi:10.1017/S0140525X18002534

Biela, A. (1986). Toward a synthetic approach to intelligence. Behavioral and Brain Sciences, 9(3), 578-579. doi:10.1017/S0140525X00047270

Biela A. (2015). Przedmowa do wydania polskiego. In J. Grac, Psychologiczne analizy procesów interioryzacji norm moralnych. Lublin: Towarzystwo Naukowe KUL.

Bucciarelli, M., Khemlani, S., & Johnson-Laird, P. N. (2008). The psychology of moral reasoning. *Judgment and Decision Making*, 3(2), 121–139.

Calmeiro, L., Stoll, S. K., & Davis, P. (2018). Moral Reasoning in Sport: Validation of the Portuguese Version of the RSBH Value-Judgement Inventory in Adolescents. Sport Science Review, 24(5–6), 285–304. https://doi.org/10.1515/ssr-2015-0020

Candee, D. (1976). Structure and choice in moral reasoning. Journal of Personality and Social Psychology, 34(6), 1293–1301. https://doi.org/10.1037/0022-3514.34.6.1293

Chih-Feng Chien, Ching-Jung Liao, Brent G. Walters, & Ching-Yieh Lee. (2016). Measuring the Moral Reasoning Competencies of Service-Learning e-Tutors. Journal of Educational Technology & Society, 19(3), 269-281.

Choi, Y. J., Han, H., Dawson, K. J., Thoma, S. J., & Glenn, A. L. (2019). Measuring moral

reasoning using moral dilemmas: evaluating reliability, validity, and differential item functioning of the behavioural defining issues test (bDIT). European Journal of Developmental Psychology, 16(5), 622–631. https://doi.org/10.1080/17405629.2019.1614907

Crockett, M. J. (2013). Models of morality. Trends in Cognitive Sciences, 17(8), 363-366. https://doi.org/10.1016/j.tics.2013.06.005

Cushman, F., Young, L., & Hauser, M. (2006). The role of conscious reasoning and intuition in moral judgment: Testing three principles of harm. Psychological Science, 17(12), 1082–1089. DOI: 10.1111/j.1467-9280.2006.01834.x

Czyżowska D., Niemczyński A. (2006) Universality of Socio-moral Development: a cross-sectional study in Poland, Journal of Moral Education, 25:4, 441-453. DOI: 10.1080/0305724960250405

Dewey J. (1910). How We Think. Boston: D. C. Heath & Co., Publishers.

Ellemers, N., van der Toorn, ., Paunov, Y. & van Leeuwen, T. (2019). The Psychology of Morality: A Review and Analysis of Empirical Studies Published from 1940 through 2017, Personality and Social Psychology Review, 23 (4), 332-366. https://doi.org/10.1177/1088868318811759

Fang, Z., Jung, W. H., Korczykowski, M., Luo,L., Kristin Prehn,K., Xu, S., Detre,J., A., Kable, J. W., Robertson, D. C., & Rao, H. (2017). Post-conventional moral reasoning is associated with increased ventral striatal activity at rest and during task. Scientific Reports, 7 (1). DOI: 10.1038/s41598-017-07115-w

Forte, A. Business Ethics: A Study of the Moral Reasoning of Selected Business Managers and the Influence of Organizational Ethical Climate. Journal of Business Ethics 51, 167–173 (2004). https://doi.org/10.1023/B:BUSI.0000033610.35181.ef

Gehrlein, J., Crede, A. K., & Adrian, N. (2020). The impact of markets on moral reasoning: Evidence from an online experiment. Journal of Behavioral and Experimental Economics , 87, 101577. https://doi.org/10.1016/j.socec.2020.101577

Gibbs, J. C. (2006). Should Kohlberg's cognitive developmental approach to morality be replaced with a more pragmatic approach? Comment on Krebs and Denton. Psychological Review, Vol. 113(3), July, 666-671. DOI: 10.1037/0033-295X.113.3.666

Grac J. (2008). Psychologia mravnosti v teoretickych a empirickych analizach. Trnava: Filozoficka fakulta Trnavskej univerzity.

Grac J. (2015). Psychologiczne analizy procesów interioryzacji norm moralnych. Lublin: Towarzystwo Naukowe KUL.

Greene, J. D., Cushman, F. A., Stewart, L. E., Lowenberg, K., Nystrom, L. E., & Cohen, J. D. (2009). Pushing moral buttons: The interaction between personal force and intention in moral judgment. Cognition, 111(3), 364–371. DOI: 10.1016/j.cognition.2009.02.001

Kamali, F., Yousefy, A., & Yamani, N. (2019). Design and psychometric properties of an instrument to assess metacognition in moral reasoning in medicine. Nursing Open, 6(4), 1331–1345. https://doi.org/10.1002/nop2.331

Kohlberg, L. (1969). Stage and Sequence: The Cognitive Developmental Approach to Socialization. In D. Goslin, (Ed.), Handbook of Socialization Theory and Research (347-480). Chicago, IL: Rand McNally.

Kohlberg, Lawrence (1971). From Is to Ought: How to Commit the Naturalistic Fallacy and Get Away with It in the Study of Moral Development. New York: Academic Press.

Kohlberg, L. (1976). Moral stages and moralization: The cognitive developmental approach. In T. Lickona (Ed.),Moral development and behavior: Theory, research and social issues (31-53). New York, NY: Holt Rinehart and Winston.

Kohlberg, L., Candee, D. (1984). The relationship of moral judgment to moral action. In L. Kohlberg (Ed.), Essays in moral development: Vol. 2. The psychology of moral development (pp. 498 –581). New York: Harper & Row.

Hanna, L. A., Gillen, J., & Hall, M. (2017). An investigation of final year pharmacy students' moral reasoning ability, and their views on professionalism and fitness to practice panel determinations: A questionnaire study. Currents in Pharmacy Teaching and Learning, 9(4), 652–659. DOI: 10.1016/j.cptl.2017.03.016

Millar, J. (2014). The Tunnel Problem, *Practical Ethics*, http://blog.practicalethics.ox.ac.uk/contact/

Moll, J., & de Oliveira-Souza, R. (2007). Moral judgments, emotions and the utilitarian brain. Trends in cognitive sciences, 11(8), 319–321. https://doi.org/10.1016/j.tics.2007.06.001

Moore G. E. (2005). Ethics. Oxford, Oxford University Press.

Nikolaicev, B. O. (1976). Osoznavaemoe i neosoznavaemoe v nravstvennom povedenii licnosti. Moskva: Izdavatelstvo Moskovskogo universiteta.

Paciello, M., Fida, R., Tramontano, C., Cole, E., & Cerniglia, L. (2013). Moral dilemma in adolescence: The role of values, prosocial moral reasoning and moral disengagement in helping decision making. European Journal of Developmental Psychology, 10(2), 190-205. https://doi.org/10.1080/17405629.2012.759099

Patil, I., Zucchelli, M. M., Kool, W., Campbell, S., Fornasier, F., Calò, M., … Cushman, F. (2020). Reasoning Supports Utilitarian Resolutions to Moral Dilemmas Across Diverse Measures. Journal of Personality and Social Psychology, (January). https://doi.org/10.1037/pspp0000281

Piaget, J. (1932). The Moral Judgment of the Child. The Free Press, New York.

Piaget J (1957). The construction of reality in the child. New York: Basic Books.

Piaget J. (1976) Piaget's Theory. In B. Inhelder, H. H. Chipman, C. Zwingmann (Eds.), Piaget and His School. A Reader in Developmental Psychology (11-23). New York, Berlin, Heidelberg: Springer-Verlag New York Inc.

Rest J. R. (1973). The hierarchical nature of stages of moral judgment. Journal of Personality, 41 (1), 86-109. https://doi.org/10.1111/j.1467-6494.1973.tb00662.x

Rest J. R. (1975). Recent Research on an Objective Test of Moral Judgment: How the Important Issues of a Moral Dilemma Are Defined. In D. J. DePalma, M. Foley (Eds.), Moral Development. Current Theory and Research (75-93). Hillsdale, New Jersey: Lawrence Erlbaum Associates, Publishers.

Rhim, J., Lee, G. bbeum, & Lee, J. H. (2020). Human moral reasoning types in autonomous vehicle moral dilemma: A cross-cultural comparison of Korea and Canada. Computers in Human Behavior, 102(August 2019), 39–56. https://doi.org/10.1016/j.chb.2019.08.010

Roche, C., Thoma S. (2017). Insights From the Defining Issues Test on Moral Reasoning Competencies Development in Community Pharmacists. American Journal of Pharmaceutical Education; 81 (8) Article 5913, 21-32. DOI: https://doi.org/10.5688/ajpe5913

Shou, Y., & Song, F. (2017). Decisions in moral dilemmas: The influence of subjective beliefs in outcome probabilities. Judgment and Decision Making. 12(5), 481–490.

Sternberg, R.J. (1984).Toward a triarchic theory of human intelligence, Behavioral and Brain Sciences, 7(2), 269-289. doi:10.1017/S0140525X00044629

Sternberg, R.J. (1986). Alternatives to the triarchic theory of intelligence, Behavioral and Brain Sciences, 9(3), 581-583. doi:10.1017/S0140525X00047300

Sudić, M., Valerjev, P., & Ćirić, J. (2019). Deontic moral reasoning task: Is moral reasoning special? Psihologijske Teme, 28(3), 483–506. https://doi.org/10.31820/pt.28.3.2

von Grundherr, M., Geisler, A., Manuel Stoiber, M. & Schäfer, M. (2017). School Bullying and Moral Reasoning Competence, Social Development 26 (2), 278-294. https://doi.org/10.1111/sode.12199

Williams R. N., Gantt E. E. (2012). Felt moral obligation and the moral judgment–moral action gap: toward a phenomenology of moral life. Journal of Moral Education Vol. 41, No. 4, December, 417–435. https://doi.org/10.1080/03057240.2012.665587

Wilmoth G. H., McFarlang S. G. (1977). A Comparison of Four Measures of Moral Reasoning. Journal of Personality Assessment, 41, 4, 396-401. https://doi.org/10.1207/s15327752jpa4104_11

ANSWERS FOR THE REVIEWERS

For Reviewer #1

Your comments were, indeed, a well-chosen remedy for the first part of the article. I treated them as good indicators for improving the internal consistency of this part of the study in terms of its substantive coherence. Hence, at your suggestion, Part One has generally been reorganized as follows:

1. The main goal of the paper is stated at the very beginning, i.e. the presentation of Jan Grác's (2008) experimental approach to the study of moral reasoning with the possibility of controlling some indicators of morality as independent variables. This methodological approach is presented first in the context of the classic approaches to moral reasoning, and then in the latest contemporary research, with particular emphasis on the possibility of experimental exploration of this research field.
2. After pointing to some contemporary authors known in the literature on the subject, I turned my attention to Piaget and Kohlberg as the classic authors of research on moral reasoning. At the same time, I indicated the relationship between their concepts and methodologies and Grác's proposed methodology.
3. At the same time, a significant number of paragraphs have been completely removed from the text of the article as being inconsistent with the main goal of our analysis.
4. I consider the meta-analysis to be an essential part of the introduction. However, I have limited myself (in accordance with your recommendation) to relations with those items which directly concern experimental research on moral reasoning.
5. After these preliminary analyses, J. Grác's theoretical and methodological statements on moral reasoning are presented in more detail (pp.10-14 of the reedited manuscript).
6. Regarding your comments on Kant's CI, I have decided to refer to his fundamental work in this area for his own definition (E. Kant (1966). Groundwork of the Metaphysics of Morals, in I. Kant, Practical Philosophy, translated by Mary Gregor, Cambridge; Cambridge University Press, Chapter 4, page 421).
7. Regarding the "no systematic exposure of indicators," the corresponding paragraph in the text has been reformulated.

8. The use of the Venn diagram in the analysis of the range relations between three different names of standards (moral, legal, executive) is justified by the necessity to strictly distinguish them in research practice. In this spirit, an appropriate paragraph explaining this issue was added to the text.

For Reviewer #2

1. As the first remarks of Reviewer # 2 are in harmony with the remarks of Reviewer # 1, we respond that the introductory part has been reworked towards making it more coherent and shorter.
2. Thanks for your attention regarding mixing the use of symbols in formalization formulas. In the end, I decided to standardize the symbolism in accordance with the notation of set theory, as these formalizations basically relate to the range relations between set names designates (e.g. moral, legal, executive norms in a Venn diagram, or in moral evaluation, which is also based on set operations).
3. You are right about the ambiguity of the experimental procedure presented in the first version of our manuscript and of the tested persons. Your objections on this matter are of vital importance. Hence, our work required clarification of such issues as: 1) the studied groups of students; 2) specification of which part of the experiment they participated in; and 3) presentation of the results of research on the strategies used by the respondents to evaluate ethical stories in the second part of the experiment. For this reason, the authors of this study decided to select two other groups of young people applying to study psychology for the second part of the experiment, i.e. for overall evaluation of the ethical stories (modeled in the first part of the experiment) as subjects. They had just completed secondary school and were taking their entrance exams for psychology at the University of Trnava. These subjects are called Subject Group 2.Their number is - 450 (N=450). The relevant fragments entered in the manuscript are marked in red.
4. Your comments related to the Formula (1). I decided to precise the notion of this formula as follows:

$$CN = \{E, M, L, (E \cap M), (L \cap M), (E \cap L) (E \cap M \cap L)\}$$

I am operating with the symbol $\dot{M}$ (and also with M) which denotes the subclass of moral norms that do not belong conjunctively either to executive norms or to

legislative ones. This symbol refers to the moral norms which I have called pure or context-free moral norms. I have marked these items in red. The symbol M in our analysis denotes the universal class of moral norms.

5. Thank you very much for the very insightful remarks on formal defects in the records of some symbols (as in Formula 7 and formulations concerning the third regularity of general moral evaluation according to the third rule of the IAO scheme). Appropriate adjustments have been made.

For Reviewer # 3

1. Your first critical remark concerns whether the IAO / KCR schemes used in the experiment are a kind of limiting template imposed on ethical stories. You also gave interesting and inspiring comparisons of our experiments with other paradigms of research on moral reasoning. An example may be the classic "trolley problem" with references to indicators of morality in moral schemes of the IAO / KCR. Thank you for this suggestion, as the classic "trolley problem" could be positioned within the moral framework of our experiment in future research.

   It is true that in every experiment the research scheme, often called a paradigm, and in our experiment a cognitive schema (IAO and KCR), limits the freedom of the experimenter and the subjects. This is the downside to all experiments by definition. However, this limitation ensures in our experiment both the possibility for the experimenter to control the independent variables (IAO morality indicators in one scheme and KCR indicators in the other scheme) as well as the precision of this control by significantly reducing the contaminating influence of uncontrolled factors on the dependent variable – which is the general moral evaluation of the ethical story. As you noticed in your reporting part of the review, the design of the experiment provides for precise control within one cognitive schema, combinatorically defined 27 values of three indicators of morality. Due to the bounded rationality of the human mind, more indicators of morality cannot be included in one framework. The selection of only two morality indicators for experimental control gives the possibility of precise control of 9 values of these variables (+, -, O), but the two-indicator moral schemas seemed to have too little

context. Therefore, it was decided to choose Aristotle's "golden mean" in our experiment.

2. We have simply avoided the claim that our experiment is “natural.” Your arguments convince us.
3. What characterizes executive norms? They are unambiguous regulators of the behavior manifested in specific situations, e.g. in contact with nature, with other people, with oneself. They are oriented towards the *hic et nunc* point of view, which is the optimum performance of certain activities. For example, in a sport, a sports record can be quickly replaced by another sports record (ski jumping hill record marked in green to be visible to the jumpers). Another clear feature of this type of normative regulation is that although they improve human activities and accompany various mental experiences (e.g. attitudes, motives), the content of these experiences themselves is not subject to assessment by these norms. Thus, it can be said that neither the views nor the feelings accompanying the performance of these activities are subject to regulation within the framework of moral norms.
4. Your critical remark concerns the use of the term "moral norm" in two different senses: (a) as a virtue (e.g., courage) in the sense of Aristotle's list of virtues; and (b) as a normative rule or moral principle. We would like to clarify that in the first sense we are referring to moral content by basically discussing some items from the subject literature in the introductory part of the article. In its revised version, many of these items have been abandoned and therefore are not included in the text. However, we cannot take advantage of your suggestion to replace the meaning (b) of the term with the meaning (a), because it is fundamental to Grác’s concept and necessary in the conceptual-methodological system of our entire experiment.
5. We agree with the comments on G.E. Moor, and therefore we have excluded these text fragments from the revised manuscript as unrelated to the merit of this article.
6. In our revised version of the article you can find the appropriate formulation of Categorical Imperative based on Kant’s (E. Kant (1966) Groundwork of the Metaphysics of Morals, in I. Kant, Practical Philosophy, translated by Mary Gregor, Cambridge; Cambridge University Press, Chapter 4, page 421).
7. As far as the concepts “mravnost” and “moralnost” are concerned, a special short paragraph has been prepared which synthetically explores the novelty of these concepts.
