# Supplementary material for: Can moral reasoning be modeled in an experiment?
Source: PLoS One. 2021 Jun 10;16(6):e0252721. doi: 10.1371/journal.pone.0252721 (PMC8191952; doi:10.1371/journal.pone.0252721)
Supplement: Attachment — Submitted filename: Response to Reviewers.docx [file pone.0252721.s004.docx]

ANSWERS FOR THE REVIEWERS

For Reviewer #1

Your comments were, indeed, a well-chosen remedy for the first part of the article. I treated them as good indicators for improving the internal consistency of this part of the study in terms of its substantive coherence. Hence, at your suggestion, Part One has generally been reorganized as follows:

1. The main goal of the paper is stated at the very beginning, i.e. the presentation of Jan Grác’s (2008) experimental approach to the study of moral reasoning with the possibility of controlling some indicators of morality as independent variables. This methodological approach is presented first in the context of the classic approaches to moral reasoning, and then in the latest contemporary research, with particular emphasis on the possibility of experimental exploration of this research field.
2. After pointing to some contemporary authors known in the literature on the subject, I turned my attention to Piaget and Kohlberg as the classic authors of research on moral reasoning. At the same time, I indicated the relationship between their concepts and methodologies and Grác’s proposed methodology.
3. At the same time, a significant number of paragraphs have been completely removed from the text of the article as being inconsistent with the main goal of our analysis.
4. I consider the meta-analysis to be an essential part of the introduction. However, I have limited myself (in accordance with your recommendation) to relations with those items which directly concern experimental research on moral reasoning.
5. After these preliminary analyses, J. Grác's theoretical and methodological statements on moral reasoning are presented in more detail (pp.10-14 of the reedited manuscript).
6. Regarding your comments on Kant's CI, I have decided to refer to his fundamental work in this area for his own definition (E. Kant (1966). Groundwork of the Metaphysics of Morals, in I. Kant, Practical Philosophy, translated by Mary Gregor, Cambridge; Cambridge University Press, Chapter 4, page 421).
7. Regarding the “no systematic exposure of indicators,” the corresponding paragraph in the text has been reformulated.
8. The use of the Venn diagram in the analysis of the range relations between three different names of standards (moral, legal, executive) is justified by the necessity to strictly distinguish them in research practice. In this spirit, an appropriate paragraph explaining this issue was added to the text.

For Reviewer #2

1. As the first remarks of Reviewer # 2 are in harmony with the remarks of Reviewer # 1, we respond that the introductory part has been reworked towards making it more coherent and shorter.
2. Thanks for your attention regarding mixing the use of symbols in formalization formulas. In the end, I decided to standardize the symbolism in accordance with the notation of set theory, as these formalizations basically relate to the range relations between set names designates (e.g. moral, legal, executive norms in a Venn diagram, or in moral evaluation, which is also based on set operations).
3. You are right about the ambiguity of the experimental procedure presented in the first version of our manuscript and of the tested persons. Your objections on this matter are of vital importance. Hence, our work required clarification of such issues as: 1) the studied groups of students; 2) specification of which part of the experiment they participated in; and 3) presentation of the results of research on the strategies used by the respondents to evaluate ethical stories in the second part of the experiment. For this reason, the authors of this study decided to select two other groups of young people applying to study psychology for the second part of the experiment, i.e. for overall evaluation of the ethical stories (modeled in the first part of the experiment) as subjects. They had just completed secondary school and were taking their entrance exams for psychology at the University of Trnava. These subjects are called Subject Group 2.Their number is - 450 (N=450). The relevant fragments entered in the manuscript are marked in red.
4. Your comments related to the Formula (1). I decided to precise the notion of this formula as follows:

CN = {E, M, L, (E ⋂ M), (L ⋂ M), (E ⋂ L) (E ⋂ M ⋂ L)}

I am operating with the symbol Ṁ ( and also with M) which denotes the subclass of moral norms that do not belong conjunctively either to executive norms or to legislative ones. This symbol refers to the moral norms which I have called pure or context-free moral norms. I have marked these items in red. The symbol M in our analysis denotes the universal class of moral norms.

5. Thank you very much for the very insightful remarks on formal defects in the records of some symbols (as in Formula 7 and formulations concerning the third regularity of general moral evaluation according to the third rule of the IAO scheme). Appropriate adjustments have been made.

For Reviewer # 3

1. Your first critical remark concerns whether the IAO / KCR schemes used in the experiment are a kind of limiting template imposed on ethical stories. You also gave interesting and inspiring comparisons of our experiments with other paradigms of research on moral reasoning. An example may be the classic "trolley problem" with references to indicators of morality in moral schemes of the IAO / KCR. Thank you for this suggestion, as the classic "trolley problem" could be positioned within the moral framework of our experiment in future research.

It is true that in every experiment the research scheme, often called a paradigm, and in our experiment a cognitive schema (IAO and KCR), limits the freedom of the experimenter and the subjects. This is the downside to all experiments by definition. However, this limitation ensures in our experiment both the possibility for the experimenter to control the independent variables (IAO morality indicators in one scheme and KCR indicators in the other scheme) as well as the precision of this control by significantly reducing the contaminating influence of uncontrolled factors on the dependent variable – which is the general moral evaluation of the ethical story. As you noticed in your reporting part of the review, the design of the experiment provides for precise control within one cognitive schema, combinatorically defined 27 values of three indicators of morality. Due to the bounded rationality of the human mind, more indicators of morality cannot be included in one framework. The selection of only two morality indicators for experimental control gives the possibility of precise control of 9 values of these variables (+, -, O), but the two-indicator moral schemas seemed to have too little context. Therefore, it was decided to choose Aristotle's "golden mean" in our experiment.

2. We have simply avoided the claim that our experiment is “natural.” Your arguments convince us.
3. What characterizes executive norms? They are unambiguous regulators of the behavior manifested in specific situations, e.g. in contact with nature, with other people, with oneself. They are oriented towards the *hic et nunc* point of view, which is the optimum performance of certain activities. For example, in a sport, a sports record can be quickly replaced by another sports record (ski jumping hill record marked in green to be visible to the jumpers). Another clear feature of this type of normative regulation is that although they improve human activities and accompany various mental experiences (e.g. attitudes, motives), the content of these experiences themselves is not subject to assessment by these norms. Thus, it can be said that neither the views nor the feelings accompanying the performance of these activities are subject to regulation within the framework of moral norms.
4. Your critical remark concerns the use of the term "moral norm" in two different senses: (a) as a virtue (e.g., courage) in the sense of Aristotle's list of virtues; and (b) as a normative rule or moral principle. We would like to clarify that in the first sense we are referring to moral content by basically discussing some items from the subject literature in the introductory part of the article. In its revised version, many of these items have been abandoned and therefore are not included in the text. However, we cannot take advantage of your suggestion to replace the meaning (b) of the term with the meaning (a), because it is fundamental to Grác’s concept and necessary in the conceptual-methodological system of our entire experiment.
5. We agree with the comments on G.E. Moor, and therefore we have excluded these text fragments from the revised manuscript as unrelated to the merit of this article.
6. In our revised version of the article you can find the appropriate formulation of Categorical Imperative based on Kant’s (E. Kant (1966) Groundwork of the Metaphysics of Morals, in I. Kant, Practical Philosophy, translated by Mary Gregor, Cambridge; Cambridge University Press, Chapter 4, page 421).
7. As far as the concepts “mravnost” and “moralnost” are concerned, a special short paragraph has been prepared which synthetically explores the novelty of these concepts.
