# Supplementary material for: Can moral reasoning be modeled in an experiment?
Source: PLoS One. 2021 Jun 10;16(6):e0252721. doi: 10.1371/journal.pone.0252721 (PMC8191952; doi:10.1371/journal.pone.0252721)
Supplement: Attachment — Submitted filename: Response to Reviewers 2.docx [file pone.0252721.s006.docx]

Answers for Reviewers

For Reviewer #1

1. Thanks for your immense work on our manuscript, which is evident both in formal review comments and particularly in your red color comments on the PDF manuscript version. What did we do? We decided to make our manuscript more coherent and took your points of criticism concerning the presence of J. Dewey and I. Kant in our paper more seriously.
2. You can see from the actual version of the manuscript that J. Dewey and I. Kant are now discussed in our paper after your inspirative remarks.

For Reviewer #3

1. We are also grateful to Reviewer 3 for a creative discussion of the concepts of mravnost and morality and for making his own suggestion as to the distinction between the two. We have taken the liberty of securing these comments in an appropriate footnote in our article.
2. After considering your points about the evaluation sentence regarding Slovak and Czech language users, this sentence has been removed from the text of the article.
