# Supplementary material for: Can moral reasoning be modeled in an experiment?
Source: PLoS One. 2021 Jun 10;16(6):e0252721. doi: 10.1371/journal.pone.0252721 (PMC8191952; doi:10.1371/journal.pone.0252721)
Supplement: Attachment — Submitted filename: Response to Reviewers 3.docx [file pone.0252721.s007.docx]

ANSWER TO REVIEWER#1

1. The authors express their gratitude to Reviewer # 1 for inspiration regarding the interpretation of the obtained results of empirical research in terms of *the natural Kantian society.* This has been expressed in footnote # 5.
2. In the last part of our paper, we have made the appropriate changes in the manuscript in accordance with your suggestions.
3. However, we cannot agree with your argumentation where you are claiming that our assertion concerning “any” is of a hyperbolic kind:” The assertion distinguishing the present research from "any other research method previously used in experimental psychology" is not established by sufficient review of extant psychological literature, at least not in this paper.”
4. We think that our statement can be seen as quite well documented as we considered in our paper, first of all, a monumental meta-analysis by Ellemers et all, 2019 who analyzed positions on the morality of 1940-2020 from where such conclusion can de drown directly. Moreover, our own analysis of articles from the last three years ( e.g. experiments made by Sudić & Ćirić, 2019; by Awad, 2019; and by Rhim, Lee & Lee, 2020) are also supporting the statement on the novelty of the presented in our paper research method.
